# A Universal Approach to Anchoring Chromophores onto Magnetic Scaffold for Achieving Easily Recyclable Heterogeneous Photocatalytic Systems

**DOI:** 10.1002/advs.202502342

**Published:** 2025-03-26

**Authors:** Xuan Zhan, Yikun Wang, Chenyang Sun, Yuchen Fang, Lihua Huang, Zakir Ullah, Qiang Chen, Xuejing Wang, Zheng Xing, Gangfeng Ouyang

**Affiliations:** ^1^ School of Chemical Engineering and Technology Sun Yat‐sen University Zhuhai 519082 China; ^2^ Institut de Ciència de Materials de Barcelona (ICMAB–CSIC) Consejo Superior de Investigaciones Científicas Campus Universitari de Bellaterra Cerdanyola del Vallès 08193 Spain; ^3^ Macao Institute of Materials Science and Engineering (MIMSE) Faculty of Innovation Engineering Macau University of Science and Technology Taipa Macao 999078 China

**Keywords:** chromophore, heterogeneous photocatalysis, magnetic beads, recyclable catalyst

## Abstract

Photocatalysis based on chromophores such as porphyrin, coumarin, anthraquinone, and pyrene is a promising technology to achieve green synthesis of various high‐value chemicals, but the robust and non‐covalent immobilization of chromophores onto light‐inert scaffolds for industrialization‐oriented heterogeneous photocatalysis remains challenging. In this work, a simple and universal strategy is presented for preparing highly efficient and recyclable heterogeneous photocatalysts from chromophores, which is achieved via biotinylation of chromophore molecules and subsequent supramolecular binding of chromophore‐biotin dyads to streptavidin‐decorated magnetic beads. As an example, commercial magnetic beads modified by 5,10,15,20‐tetrakis(4‐aminophenyl) porphyrin not only possessed remarkable photocatalytic activities for the oxidative coupling of benzylamine derivatives and the oxidation of thioanisole derivatives with highest product yields of beyond 95% and turnover numbers approaching 10000, driven by photogenerated reactive oxygen species but also demonstrated impressive chemical stability and efficient recyclability via simple magnetic separation during 10 successive test cycles. The findings revealed in this work pave the way for advancing green synthesis of valuable organic compounds in the pharmaceutical industry, agricultural sector, etc., with rationally designed heterogeneous photocatalytic systems.

## Graphical abstract

The practical application of chromophores such as porphyrins, coumarins, anthraquinones, and pyrene derivatives in photocatalysis is severely limited by catalyst stability and reusability. In this work, a simple and universal strategy is presented for preparing highly efficient and recyclable heterogeneous photocatalysts for the oxidative coupling of benzylamines and the oxidation of thioanisoles through immobilizing biotinylated chromophores to streptavidin‐decorated magnetic beads.

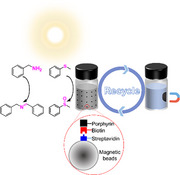

## 1 Introduction

Photocatalysis is emerging as a promising solution to various global energy‐ and environment‐related challenges by harnessing solar energy for hydrogen fuel production from water splitting, carbon dioxide reduction, pollutant degradation, synthesis of important chemicals, etc.^[^ ^1^ ^]^ For instance, chromophores such as porphyrin,^[^ ^2^ ^]^ coumarin,^[^ ^3^ ^]^ pyrene,^[^ ^4^ ^]^ anthraquinone^[^ ^5^ ^]^ and their derivatives have been extensively studied for versatile photocatalytic valued‐added organic transformations due to their excellent visible light absorption, robust generation of reactive excited states or oxygen species and the ease in tuning their electronic structures, redox properties, and photophysical characteristics.^[^ ^6^, ^7^, ^8^, ^9^ ^]^ However, while the homogeneous photocatalytic systems based on those chromophores favor the interaction between catalysts and substrate molecules, leading to relatively high product yields,^[^ ^10^ ^]^ the practical application is still severely hindered by the stability and recycling of catalyst molecules.^[^ ^11^ ^]^ A potential solution to this issue is to assemble the chromophore molecules into metal‐organic frameworks (MOFs) or covalent‐organic frameworks (COFs) with large surface areas and porosities, forming heterogeneous systems.^[^ ^12^, ^13^, ^14^, ^15^ ^]^ However, the preparation of these nanoporous materials often requires toxic catalysts and stringent conditions, which can limit their practical applicability.^[^ ^16^ ^]^ What's worse, the recovery of the catalyst material via centrifugation is inefficient and the aggregation of chromophore units due to π‐π interactions may jeopardize the catalytic activities in various solvents.^[^ ^17^, ^18^, ^19^, ^20^ ^]^

To overcome these limitations, a viable strategy involves the immobilization of chromophores onto light‐inert solid supports via non‐covalent interactions to minimize the excited‐state quenching induced by energy or charge transfer.^[^ ^21^ ^]^ However, achieving stable, non‐covalent immobilization remains challenging. Considering the simple biotinylation of small chromophores^[^ ^22^, ^23^ ^]^ and the high‐affinity supramolecular interaction between biotin and streptavidin with an exceptionally low dissociation constant (≈10^−15^ m),^[^ ^24^ ^]^ to anchor chromophore‐biotin dyads onto streptavidin‐containing magnetic scaffolds offers an ideal pathway to immobilize chromophores in a robust and non‐covalent manner. Such chromophore‐based heterogeneous photocatalytic systems present several key advantages: i) the preparation protocols are straightforward and green since the biotinylation of chromophores and the coupling of biotinylated chromophores with streptavidin occur easily under ambient conditions; ii) much more simple and yet efficient catalyst recycling than centrifugation can be achieved via magnetic separation; iii) the aggregation of chromophores can be minimized via precisely controlling the molar ratio of chromophore‐biotin dyads to streptavidin, to mitigate catalyst deactivation.

**Scheme 1:**
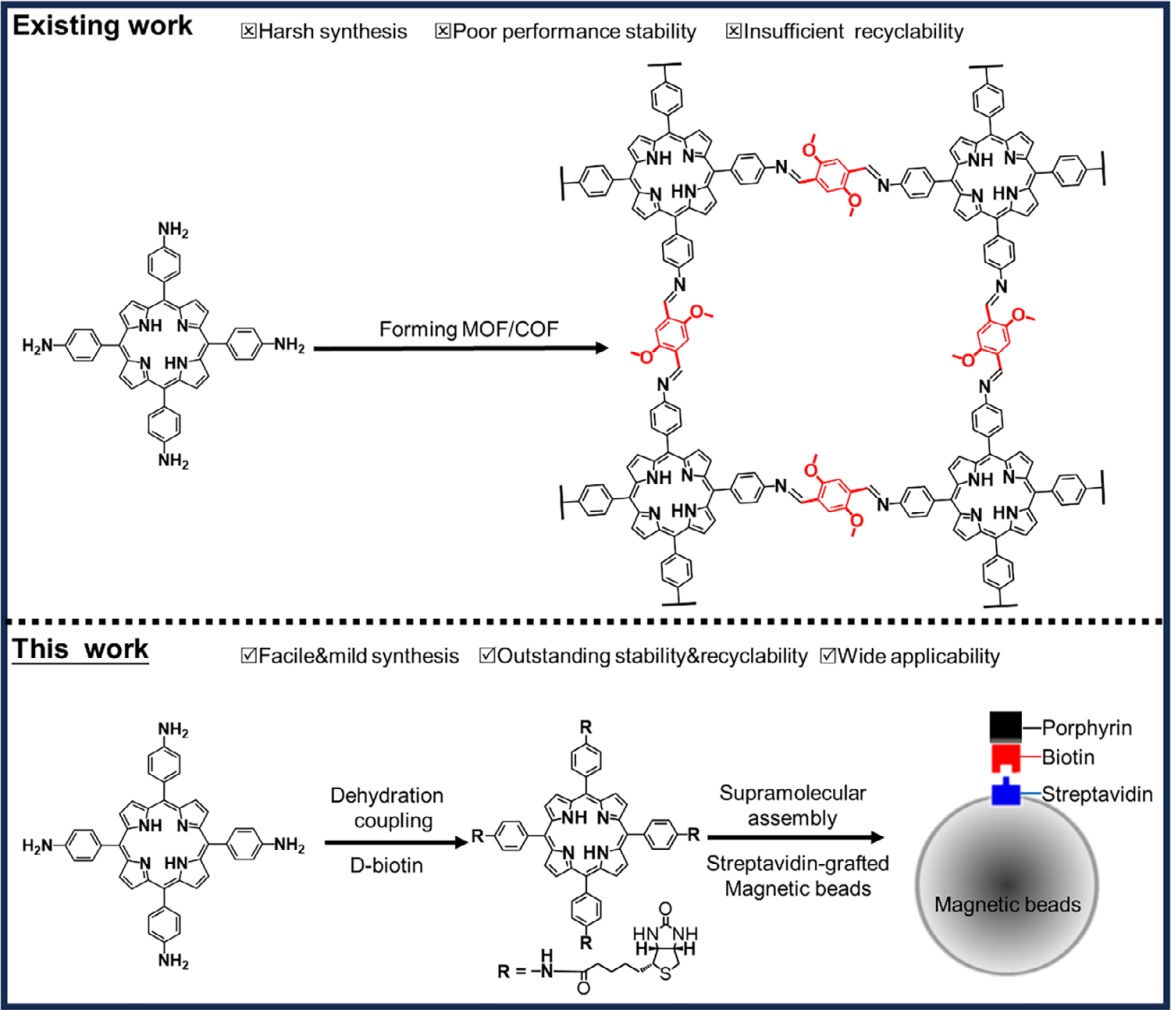
Comparison between existing strategies to construct heterogeneous photocatalytic systems from chromophores and this work, exemplified by TAPP here.

In this study, we demonstrated a universal protocol to immobilize photosensitizing molecules onto streptavidin‐decorated magnetic beads and to construct highly active and recyclable heterogeneous photocatalytic systems for the oxidative coupling of benzylamine derivatives and the oxidation of thioanisole derivatives. The synthesis of heterogeneous photocatalysts involved the biotinylation of chromophores and the subsequent supramolecular interaction between the biotin moiety and the streptavidin on the surface of magnetic beads (**Scheme** **1**). A model photosensitizer (PS), the dyad between 5,10,15,20‐tetrakis(4‐aminophenyl) porphyrin (TAPP) and biotin, was anchored onto commercially available streptavidin magnetic beads, which exhibited outstanding photocatalytic oxidative conversion performance of a number of benzylamine/thioanisole derivatives, mediated by photogenerated reactive oxygen species (ROS). More importantly, the as‐constructed heterogeneous photocatalytic system demonstrated impressive reusability unseen in MOFs/COFs assembled from chromophores, with extremely robust chemical stability and efficient catalyst recovery via magnetic separation. This strategy offers a viable route to develop highly recyclable heterogeneous photocatalysts for a wide range of applications.

## 2 Results and Discussion

### 2.1 Biotinylation of Chromophores

**Figure 1:**
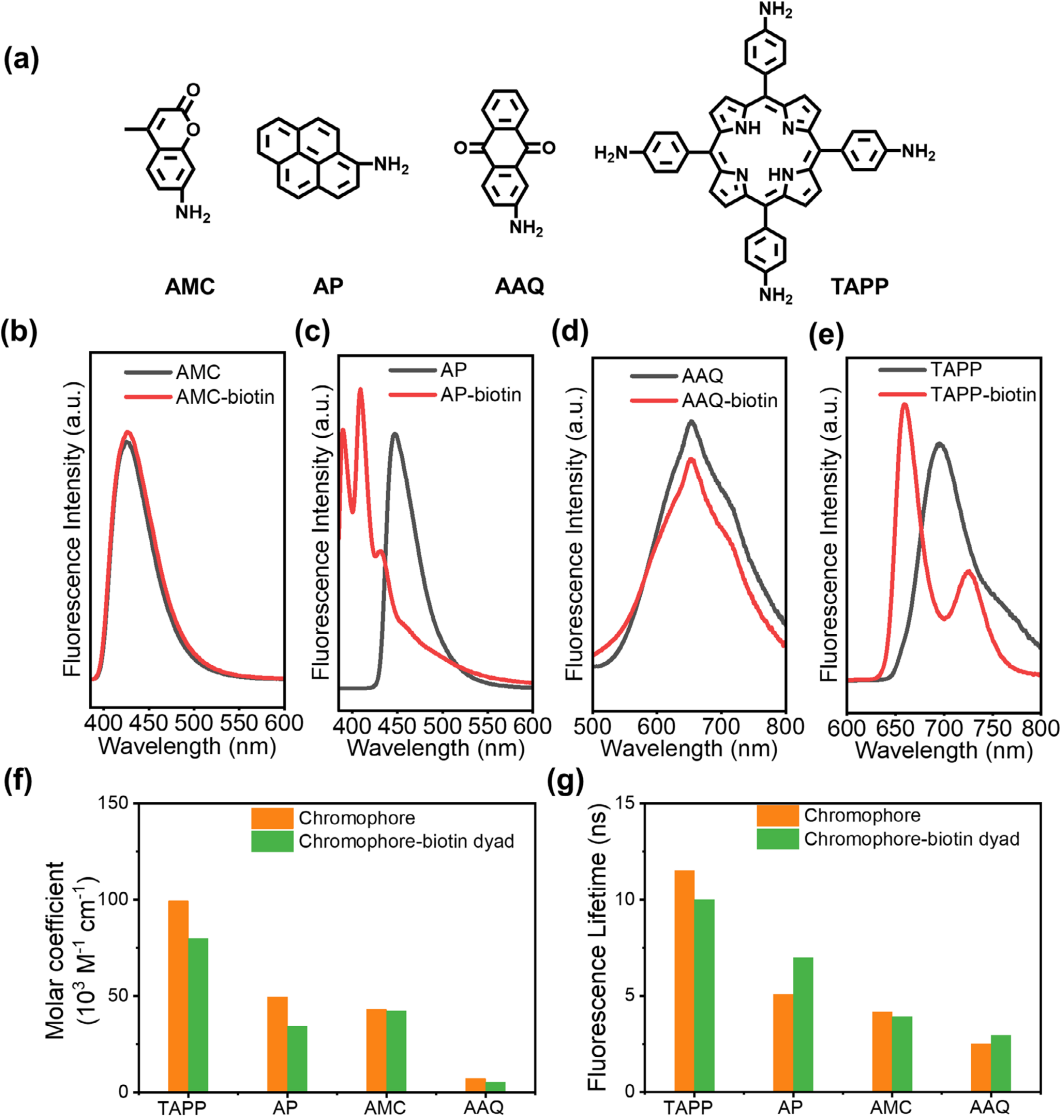
a) Chemical structures of AMC, AP, AAQ, and TAPP; the fluorescence emission spectra of b) AMC, c) AP, d) AAQ, and e) TAPP, before and after biotinylation; f) Measured molar coefficients at the absorption peak and g) Measured fluorescence lifetimes of all four chromophores and their corresponding dyads.

Four representative chromophores (**Figure** **1**a), that is, TAPP and 2‐aminoanthraquinone (AAQ), which exhibited great versatility for photocatalytic reactions,^[^ ^5^, ^25^ ^]^ and 1‐aminopyrene (AP) and 7‐amino‐4‐methylcoumarin (AMC), well‐known for their high fluorescence quantum yields,^[^ ^26^, ^27^ ^]^ were selected to form chromophore‐biotin dyads through coupling with biotin in the presence of 6 equivalents of benzotriazolyloxy‐tris(pyrrolidino)‐phosphonium hexafluorophosphate (PyBOP) and diisopropylethylamine (DIPEA), following a classical COOH‐NH_2_ dehydration approach (Scheme S1, Supporting Information).^[^ ^28^ ^]^ As shown in the proton nuclear magnetic resonance (^1^H‐NMR) spectra, the signals ranging from 5 to 9.5 ppm were mainly assigned to the protons on the chromophore moiety of the four dyads, while the resonances at less than 5 ppm mainly originated from the protons on the biotin moiety, except for the singlet signal at −2.89 ppm assigned to the inner ─NH of TAPP moiety in the case of TAPP‐biotin dyad, indicating the coupling of chromophores with biotin (Figures S1,S3–S5, Supporting Information). Moreover, the m/z value of [TAPP‐biotin+Na] was determined to be 1601.5883 (Figure S2, Supporting Information) from the corresponding matrix‐assisted laser desorption/ionization time‐of‐flight mass spectrum (MALDI‐TOF‐MS), matching the calculated value of 1601.7898. Similarly, the electrospray ionization mass spectra (ESI‐MS) of AAQ‐biotin, AP‐biotin, and AMC‐biotin dyads revealed m/z values of 448.13, 444.17, and 402.14, respectively, perfectly in line with the theoretical values of 449.14, 443.17 and 401.14. Thus, all the NMR and MS results verified the successful biotinylation of TAPP, AAQ, AP, and AMC.

To investigate the impact of biotinylation on the photophysical properties of the chromophores, the emission and optical absorption behaviors of the biotinylated dyads were analyzed. As shown in Figure 1b–e, TAPP, AAQ, AP, and AMC displayed emission peaks at 695, 653, 447, and 425 nm, respectively. Biotinylation of AAQ and AMC exerted a trivial effect on the emission properties, and only induced slight blue shifts of 37 and 35 nm of the emission peaks to AP and TAPP, respectively (Figure 1c,e and Table S1, Supporting Information), likely due to the weak electron‐donating effect of biotin moiety.^[^ ^29^ ^]^ In addition, the fluorescence lifetimes of the biotinylated dyads only changed marginally compared to their non‐biotinylated counterparts (Figure 1g). The absence of considerable fluorescence quenching and lifetime change clearly evidenced the fact that no energy or electron transfer occurred in any of the chromophore‐biotin dyads. TAPP, AAQ, AP, and AMC displayed absorption peaks at 435, 457, 389, and 370 nm (Figures S6–S9, Supporting Information), respectively. Similar to the observations from the fluorescence emission spectra, the biotinylation of AAQ and AMC induced negligible influence on their UV–vis absorption peaks at 457 and 370 nm, while the AP‐biotin and TAPP‐biotin dyads underwent blue shifts of absorbance peaks by 41 and 10 nm, respectively, as compared to the pristine counterparts (Figures S6–S9, Table S1, Supporting Information), respectively. Furthermore, as seen in Figure 1f, the biotinylation of all four chromophores resulted in insignificant decreases in the molar coefficient at the absorption peaks. In summary, biotinylation of the four representative chromophores induced only minor changes in their photophysical properties owing to the non‐aromatic nature of the biotin moiety.

### 2.2 The Immobilization of TAPP‐Biotin Dyad onto Streptavidin‐Decorated Magnetic Beads

**Figure 2:**
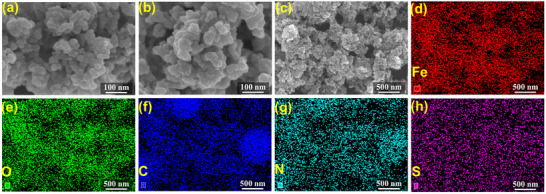
SEM images of a) commercial streptavidin‐decorated magnetic beads and b, c) the as‐prepared TMSMB; the EDS elemental maps of d) Fe, e) O, f) C, g) N, and h) S corresponding to the area of (c).

With a significantly longer average fluorescence lifetime of 10 ns and higher molar absorption coefficient (≈100 000 L mol^−1^ cm^−1^) than the other dyads, the TAPP‐biotin dyad was selected as a model photosensitizer (PS) to construct easily recyclable heterogeneous photocatalyst through immobilizing onto streptavidin‐decorated magnetic beads. As illustrated in Figure S10 (Supporting Information), the TAPP‐biotin dyad can be efficiently grafted onto streptavidin‐coated magnetic beads through the well‐established strong biotin‐streptavidin interaction.^[^ ^30^ ^]^ The unbound TAPP‐biotin was removed via thorough washing with dimethyl sulfoxide (DMSO) until the absorbance of the supernatant matched that of blank DMSO. The scanning electron microscopy (SEM) images of commercial streptavidin magnetic beads and the as‐prepared TAPP‐biotin‐streptavidin‐decorated magnetic beads (TBSMB) (**Figure** **2**a–c; Figures S11,S12, Supporting Information) revealed similar morphologies of aggregated nanospheres. The energy dispersive X‐ray spectroscopy (EDS) elemental maps of TBSMB (Figure 2d–h) confirmed the presence of uniformly distributed Fe, O, C, N, and S elements: the Fe signals originated from the Fe_3_O_4_ core of the magnetic beads; the O signal derived from the Fe_3_O_4_ core and bound TAPP‐biotin dyad; C and N stemmed from streptavidin and the TAPP dyad; S was primarily attributed to biotin, as no S was detected in the commercial magnetic beads (Figures S13,S14, Supporting Information). These results confirmed the successful immobilization of the TAPP‐biotin dyad onto streptavidin‐decorated magnetic beads. More importantly, both the TAPP‐biotin dyads alone and TBSMB only exhibited a narrow UV–vis absorption peak at ≈420 nm when dispersed in DMSO (Figure S15, Supporting Information), indicating no detectable aggregation, in stark contrast to chromophore‐based MOFs or COFs.

### 2.3 Photocatalytic Activities for the Oxidative Coupling of Benzylamine Derivatives and the Oxidation of Thioanisole Derivatives

To demonstrate the potential of the as‐prepared TBSMB as a viable photocatalyst, two classes of ROS‐driven organic reactions, that is, the oxidative coupling of benzylamine derivatives and the oxidation of thioanisole derivatives, were assessed. The imine products generated from the former coupling reactions are valuable intermediates or precursors for bioactive nitrogen‐containing compounds,^[^ ^31^ ^]^ and the sulfoxides formed from the latter reactions are valuable intermediates in organic synthesis with significant applications in pharmaceuticals and antiseptics.^[^ ^32^ ^]^

**Figure 3:**
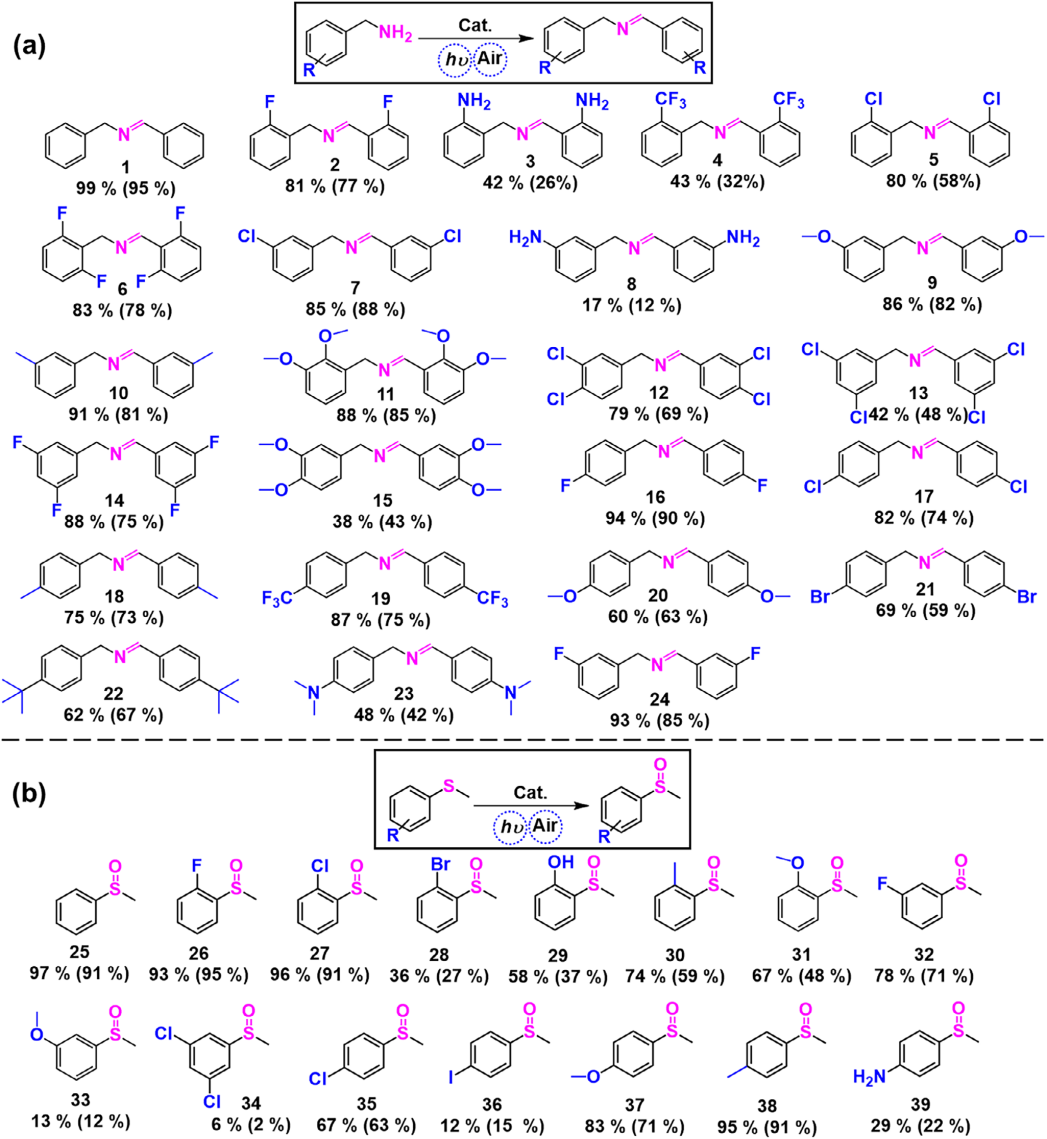
Substrate scope for a) the oxidative coupling reaction of benzylamine derivatives, and b) the oxidation of thioanisole derivatives, resulting in imine products and sulfoxide products, respectively. The yields of converting different substrates with TBSMB are listed below the corresponding chemical structures (the yields with TAPP alone are listed in the brackets) General reaction conditions: 20 µL of TBSMB (containing ≈10^−9^ mol TAPP‐biotin) in 1 mL DMSO with 0.01 m substrate, irradiated by Xe lamp for 3 h. Yields were analyzed by GC‐MS; All the related GC‐MS traces were included in the Supporting Information Section.

DMSO was selected as the solvent in all experiments to prevent catalyst aggregation. While neither streptavidin‐decorated magnetic beads nor biotin alone exhibited any catalytic activity (Table S2, Supporting Information), the as‐prepared TBSMB was capable of converting 24 benzylamine derivatives into their corresponding imine products, with yields ranging from 17% to 99% (**Figure** **3**a). In particular, the conversion percentages of substrates 1, 2, 5–7, 9–11, 14, 16–17, 19, and 24 reached above 80%, with the product yield and turnover numbers (TON, equals to moles of product per mole of catalyst) of substrate 1 transformation being as high as 99% and 10 000, respectively. It is noted that for the mono‐substituted benzylamine derivatives, TBSMB demonstrated better catalytic performance in transforming substrates substituted with ─F, ─Cl, ─CH_3,_ and ─OCH_3_ than those with ─NH_2_, ─CF_3_, ─C(CH_3_)_3_, and ─N(CH_3_)_2_, which can be attributed to several factors: i) the steric hindrance from ─C(CH_3_)_3_, ─CF_3_ and ─N(CH_3_)_2_ groups likely slowed the reaction;^[^ ^33^ ^]^ ii) the electron‐donating nature of ─CH_3_ and ─OCH_3_ helped stabilize the potential positively‐charged carbonation intermediates;^[^ ^34^ ^]^ iii) despite being electron‐withdrawing groups, ─F and ─Cl can stabilize the potentially formed positively‐charged intermediates through p‐π conjugation, donating electron density via their lone pairs to a certain extent;^[^ ^35^ ^]^ iv) the combined electron‐withdrawing and steric effects of the ─CF_3_ group destabilized the potential positively‐charged carbonation intermediates. Similarly, bis‐substituted species with ─F (14), ─OCH_3_ (11), and ─Cl (12) showed good yields (79%–88%). Surprisingly, for the oxidative coupling of the majority of benzylamine derivatives, TBSMB outperformed TAPP alone. For example, the yields of substrates 3 and 4 were 26% and 75%, respectively, for TAPP alone, and 42% and 88%, respectively, for TBSMB.

Similar to the oxidation coupling with benzylamine, neither biotin nor streptavidin magnetic beads alone produced any products from the photocatalytic oxidation of thioanisole (Table S2, Supporting Information), and TBSMB demonstrated outstanding production of corresponding sulfoxides from the conversion of a total of 15 thioanisole derivatives (Figure 3b). Nearly half of the substrates, including 2‐F/Cl substituted substrates (26 and 27), as well as 4‐OCH_3_/CH_3_ substituted substrates (37 and 38), can be oxidized by ca. 70% or higher percentages, with the highest yield of 97% obtained for thioanisole (TON ≈10 000). The electron‐withdrawing groups (─F and ─Cl) at the 2‐position can stabilize the potential intermediate sulfide cations through p‐π conjugation effects,^[^ ^35^ ^]^ while the electron‐donating groups (─OCH_3_ and ─CH_3_) at the 4‐position increased the electron density on S and made S easier to oxidize,^[^ ^36^ ^]^ which explains the high conversion percentages of such substrates. The GC‐MS analysis (Figures S56–S70, Supporting Information) revealed that trivial di‐sulfoxide by‐products (<2%) were produced in the oxidation processes of substrates 25, 26, 27, 32, 35, 37 and 38, respectively, while no by‐products were detected for the other substrates. In addition, TBSMB generally exhibited superior photocatalytic sulfoxides generation as compared to TAPP alone. All results established the broad potential of the as‐prepared TBSMB for sustainable synthesis of valuable organic compounds.

To evaluate the wavelength dependence of the photocatalytic activities of the as‐prepared TBSMB, the oxidation efficiencies of benzylamine and thioanisole were examined under fixed‐wavelength irradiation at 365, 405, 455, 505, 565, and 625 nm (Figure S16, Supporting Information). The reaction activities exhibited a strong correlation with the UV–vis absorption profile of TBSMB, demonstrating optimal photocatalytic performance at 405 nm for both reactions. Further, time‐dependent conversion studies (Figure S17, Supporting Information) were conducted which revealed that the oxidation of benzylamine achieved a maximum yield of 97% within 2 h of irradiation, while thioanisole oxidation required 2.5 h to reach a maximum yield of 95%, respectively.

### 2.4 Reaction Mechanism Studies

**Figure 4:**
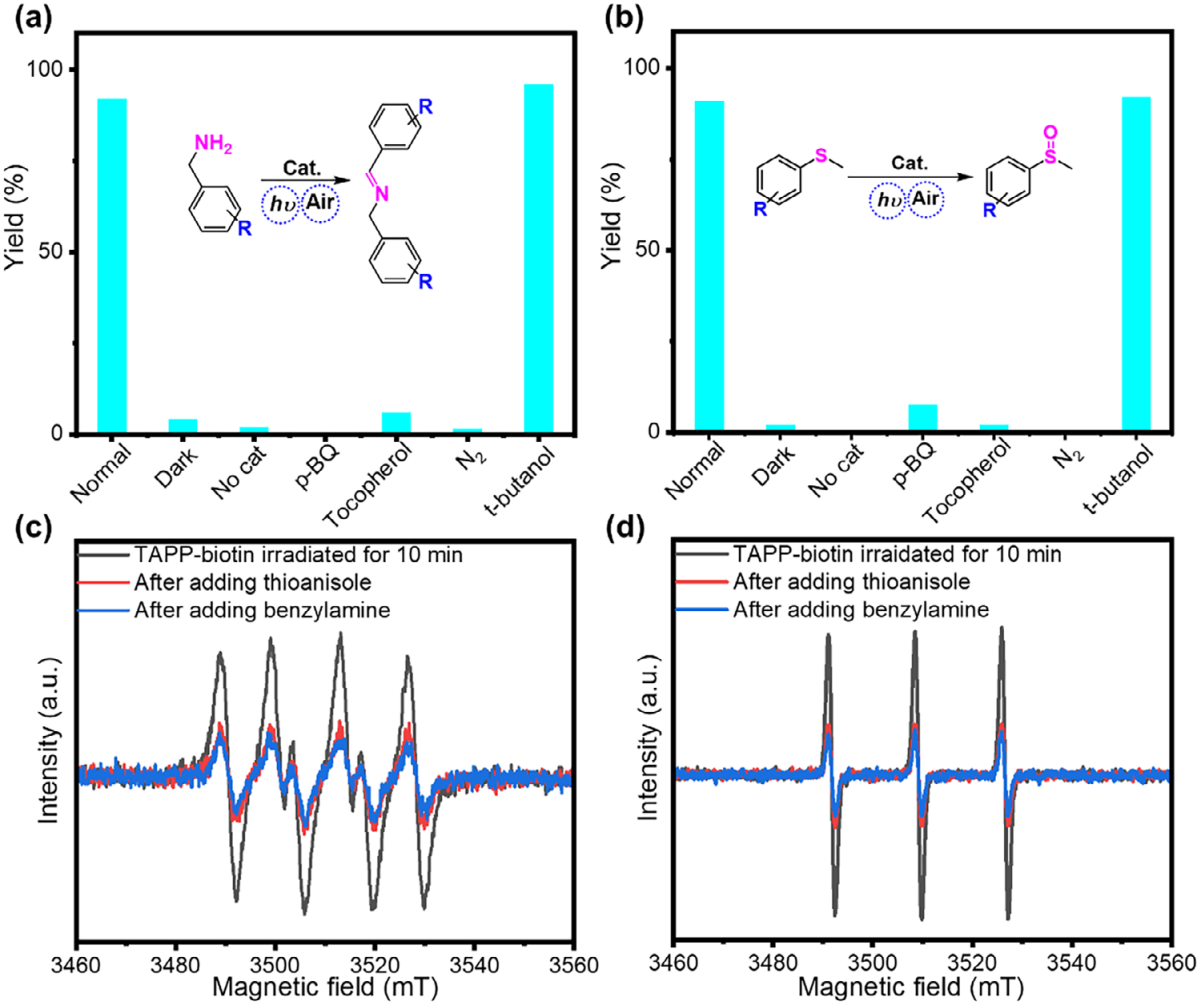
The yields of a) oxidative coupling of benzylamine and b) thioanisole oxidation obtained with TBSMB under different conditions (lighting, atmosphere, scavengers, etc.). Normal procedure: 100 µL TBSMB (5 × 10^−9 ^mol TAPP‐biotin) in 1 mL DMSO with 0.1 m benzylamine or thioanisole added; set the concentration of scavengers (p‐benzoquinone, tocopherol, butanol) at 0.2 m; irradiated with Xe lamp for 3 h. Yields were analyzed by GC‐MS. The EPR spectra of the TAPP‐biotin dyad in the presence of (c) DMPO and d) TEMP, and the corresponding change upon the addition of benzylamine or thioanisole. The concentration of added benzylamine or thioanisole in the EPR assays was 0.2 m.

The product yields of both photocatalytic oxidative coupling of benzylamine and oxidation of thioanisole were significantly suppressed to minimal values under the N_2_ atmosphere (**Figure** **4**a,b), which suggested that the reaction mechanisms involved O_2_. To gain insights into the actual ROS that drove both reactions, control experiments with the addition of a series of scavengers were conducted. P‐benzoquinone (p‐BQ), tocopherol, and butanol were used to scavenge superoxide radicals (•O_2_⁻), singlet oxygen (^1^O_2_), and OH radicals, respectively.^[^ ^37^ ^]^ The introduction of either p‐BQ or tocopherol led to a sharp decline in the conversion percentage of benzylamine from 92% (to 0.5% and 6%, for p‐BQ and tocopherol, respectively, Figure 4a), while the addition of butanol caused minimal change. Thus, both •O_2_⁻ and ^1^O_2_ played key roles in the imine formation process, with •O_2_⁻ being slightly more influential. Furthermore, the electron paramagnetic resonance (EPR) spectra of TBSMB in the presence of 5,5‐dimethyl‐1‐pyrroline N‐oxide (DMPO) and 2,2,6,6‐tetramethyl‐1‐piperidine (TEMP) under irradiation displayed strong signals of DMPO‐•O_2_⁻ and TEMP‐^1^O_2_ spin adducts, respectively (Figure 4c,d),^[^ ^38^ ^]^ both of which decreased markedly in intensities when benzylamine was added, indicative of strong reaction between benzylamine and the two ROS. Likewise, the presence of p‐BQ and tocopherol significantly reduced the sulfoxide formation (Figure 4b), with the yield dropping from 91% to 7.5% and 2%, respectively, hinting at the importance of both •O_2_⁻and ^1^O_2_ (slightly higher). The corresponding EPR assays for •O_2_⁻ and ^1^O_2_ also weakened substantially upon the addition of thioanisole (Figure 4c,d), indicating the two photogenerated ROS being consumed by thioanisole. The above‐mentioned experimental results clearly suggested two distinct pathways for the formation of imines and sulfoxides mediated by •O_2_⁻ and ^1^O_2_, which were generated through electron‐transfer and energy‐transfer processes, respectively, upon the photo‐excitation of the TAPP‐biotin dyad.

**Figure 5:**
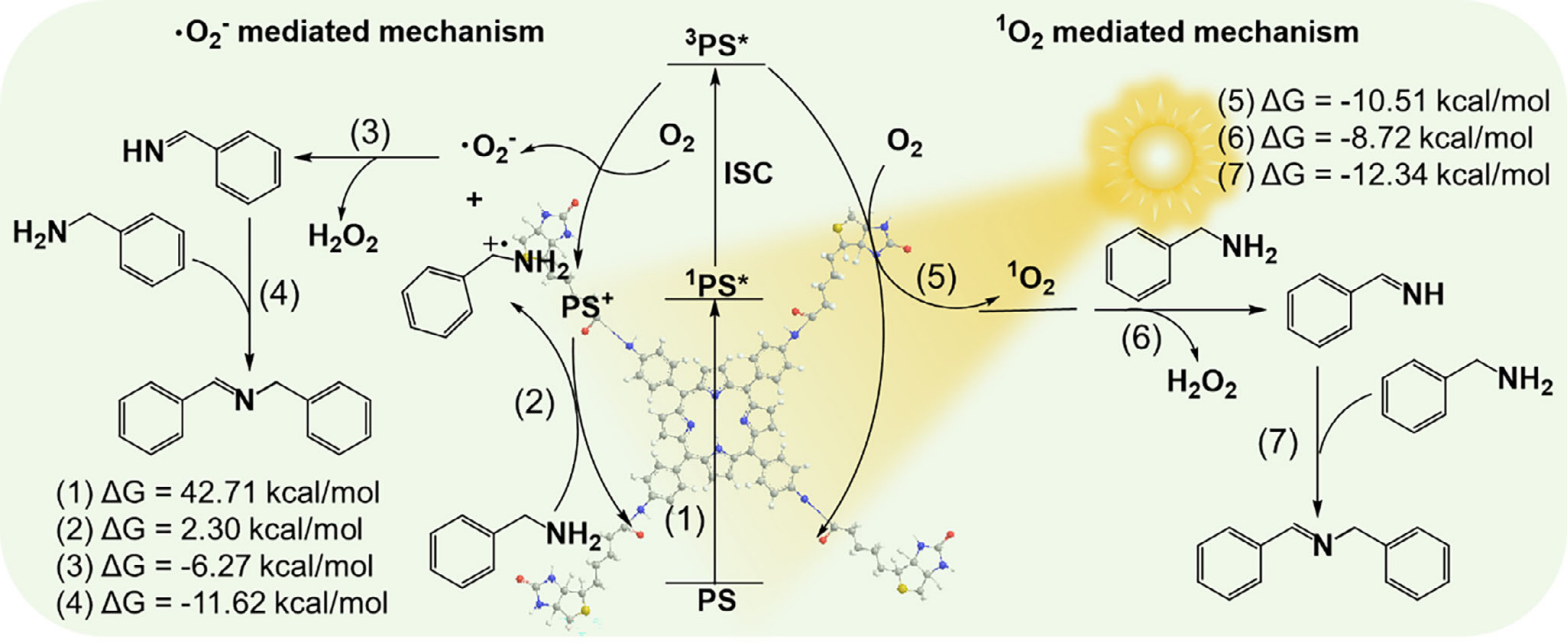
Proposed reaction mechanism for the oxidative coupling reaction of benzylamine, with the corresponding free energy (kcal mol^−1^) calculations labeled. Calculation details can be found in SI.

Density functional theory (DFT) calculations were performed to further reveal the underlying mechanisms of the photogeneration of ROS and the subsequent ROS‐driven catalytic reactions. As depicted in **Figure** **5**, upon irradiation, the TAPP‐biotin PS absorbed light and transitioned to an excited singlet state (¹PS^*^), which then underwent intersystem crossing (ISC) to a more stable triplet state (^3^PS^*^). A ^3^PS^*^ could donate an electron to an O_2_ molecule, forming a •O_2_⁻ and a PS^+^, followed by the capture of an electron from a benzylamine molecule by the PS^+^, which generated a PhCH₂NH₂⁺ radical and a ground‐state PS (with a Gibbs free energy change (ΔG) of +2.3 kcal mol^−1^). Alternatively, the energy transfer between ^3^PS^*^ and O₂ generated ¹O₂, with a ΔG of −10.5 kcal mol^−1^. For the oxidative coupling of benzylamine mediated by •O_2_⁻, a PhCH₂NH₂⁺ radical reacted with a •O_2_⁻ to produce a hydrogen‐abstracted benzylamine radical, followed by the formation of phenylmethanimine (ΔG = −6.3 kcal mol^−1^), which then reacted with another benzylamine molecule to form the imine product (ΔG = −11.6 kcal mol^−1^).^[^ ^34^ ^]^ For the formation of imine mediated by ^1^O_2_, benzylamine molecules reacted with ^1^O_2_, forming phenylmethanimine intermediates and H_2_O_2_ (ΔG = −8.7 kcal mol^−1^), and then the reaction between phenylmethanimine intermediates and benzylamine molecules yielded the imine product (ΔG = −12.3 kcal mol^−1^).^[^ ^36^ ^]^ Similar to the oxidative coupling of benzylamine, the oxidation of thioanisole to sulfoxides proceeded via two pathways (Figure S18, Supporting Information): i) PhSCH₃⁺ radicals that were formed from the oxidation of thioanisole by PS^+^ reacted with •O_2_⁻ to form peroxide intermediates (PhSO₂CH₃•), which then reacted with thioanisole to produce sulfoxide; ii) peroxide intermediate was first generated from the reaction between ^1^O₂ and thioanisole, which then reacted with thioanisole to produce sulfoxide.^[^ ^36^ ^]^

**Figure 6:**
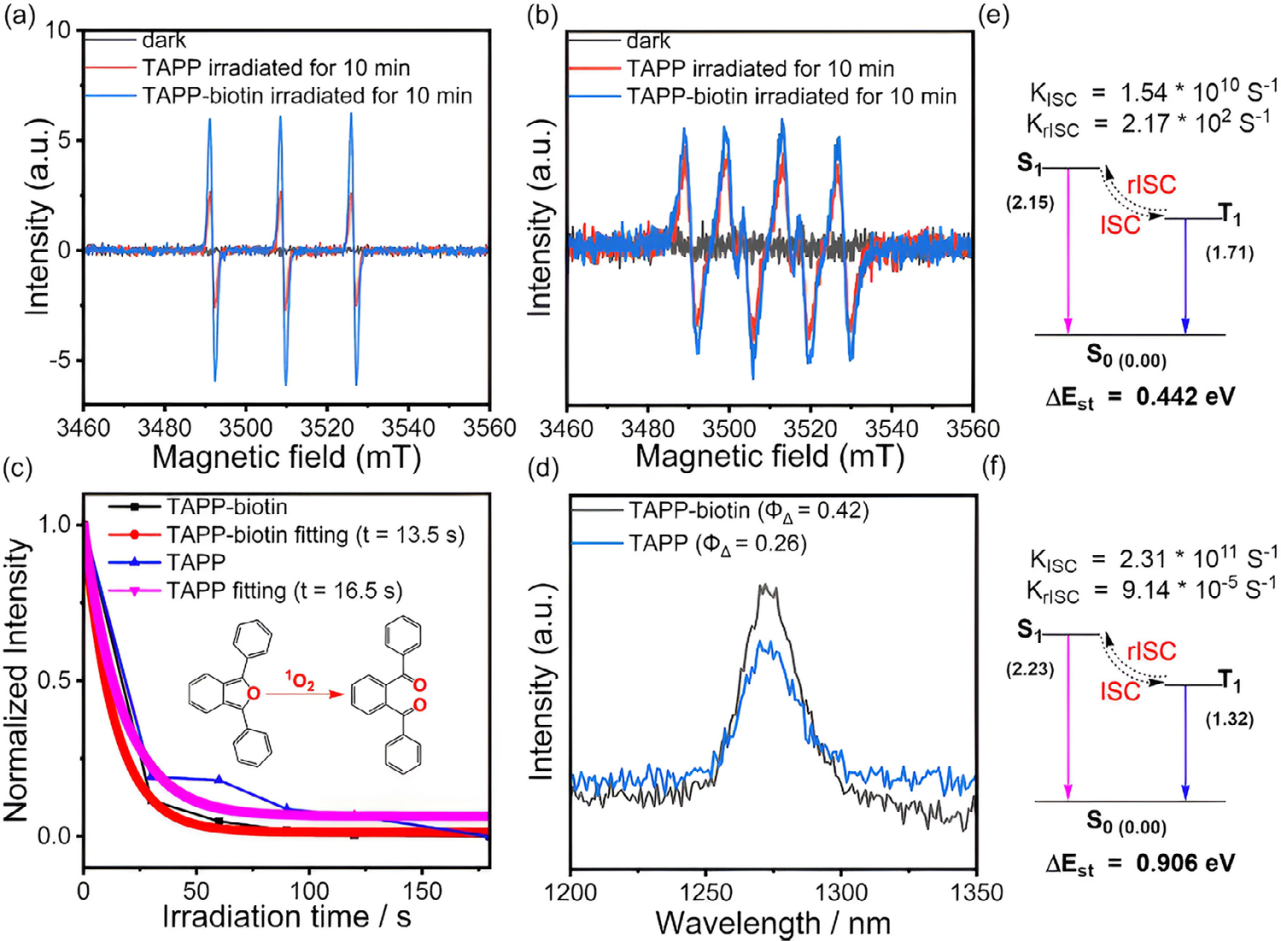
a) EPR detection of ^1^O_2_ trapped by TEMP after irradiation for 10 min under Xe lamp; b) EPR detection of •O_2_⁻ trapped by DMPO after irradiation for 10 min under Xe Lamp; c) Absorbance changes at 434 nm for TAPP and 425 nm for the TAPP‐biotin dyad monitored under Xe lamp irradiation over 180 s, with single exponential decay fitting; d) The ^1^O_2_ emission spectra (centered at ≈1275 nm) of TAPP and the TAPP‐biotin dyad in DMF; the concentration of TAPP or TAPP‐biotin was 50 µm for the ^1^O_2_ emission measurements; with their ^1^O_2_ quantum yields determined shown in the Supporting Information Section; e) Calculated energy (electron volts) emission diagram of TAPP; f) Calculated energy (electron volts) emission diagram of the TAPP‐biotin dyad. The calculation details were included in the Supporting Information Section.

To understand why TBSMB exhibited slightly better catalytic performance than TAPP alone, the capabilities of generating ROS upon photoexcitation of TAPP alone and the TAPP‐biotin dyad were compared (the TAPP‐biotin dyad was the actual catalytic unit of TBSMB). EPR measurements revealed that as compared to TAPP alone, the TAPP‐biotin dyad generated drastically intensified ^1^O_2_ signals (**Figure** **6**a) and slightly stronger signals of •O_2_⁻ radicals (Figure 6b) under Xe lamp irradiation (50 W for 10 min). In addition, the changes in the optical absorption spectra of 1,3‐diphenylisobenzofuran (DPBF) and nitro blue tetrazolium (NBT) that are scavengers for ^1^O_2_ and •O_2_⁻, respectively,^[^ ^39^ ^]^ in the presence of either the TAPP‐biotin dyad or TAPP alone were monitored at different irradiation times. While the typical absorption peak at ca. 430 nm of DPBF was quenched upon irradiation for both catalysts, the dyad caused a much more pronounced peak reduction, indicating a higher production rate of ^1^O_2_ (Figure 6c; Figure S19, Supporting Information). This finding was in agreement with the higher steady‐state luminescence emission quantum yield of ^1^O_2_ in DMF (1275 nm) determined for the dyad (Φ_Δ_ = 0.42) than that for TAPP alone (Φ_Δ_ = 0.26) (Figure 6d).^[^ ^40^ ^]^ The typical absorption peak at ca. 260 nm of NBT declined similarly upon illumination for both catalysts (Figure S20, Supporting Information), reflecting that the photogeneration of •O_2_⁻ was not affected by biotinylation.

Since ROS generation is closely related to the intersystem crossing (ISC) process of PSs under irradiation,^[^ ^6^ ^]^ the ISC rate constants of both catalysts were calculated (Figure 6e,f). The significantly higher ISC rate constant of the dyad (2.33 × 10^11^ s⁻¹) than that of TAPP (1.54 × 10^10^ s⁻¹) suggested more efficient singlet‐to‐triplet transitions, which increased the triplet‐state population necessary for the ROS generation. Moreover, the lower reverse ISC (rISC) rate constant of the dyad (9.14 × 10^−5^ s⁻¹) prolonged its triplet‐state lifetime, further enhancing the ROS production. In summary, the dyad demonstrated enhanced capability in producing ^1^O_2_ when excited by incident photons due to more efficient singlet‐to‐triplet transitions, which in turn led to superior catalytic performance.

### 2.5 Exceptional Recovering Efficiency and Catalyst Stability

**Figure 7:**
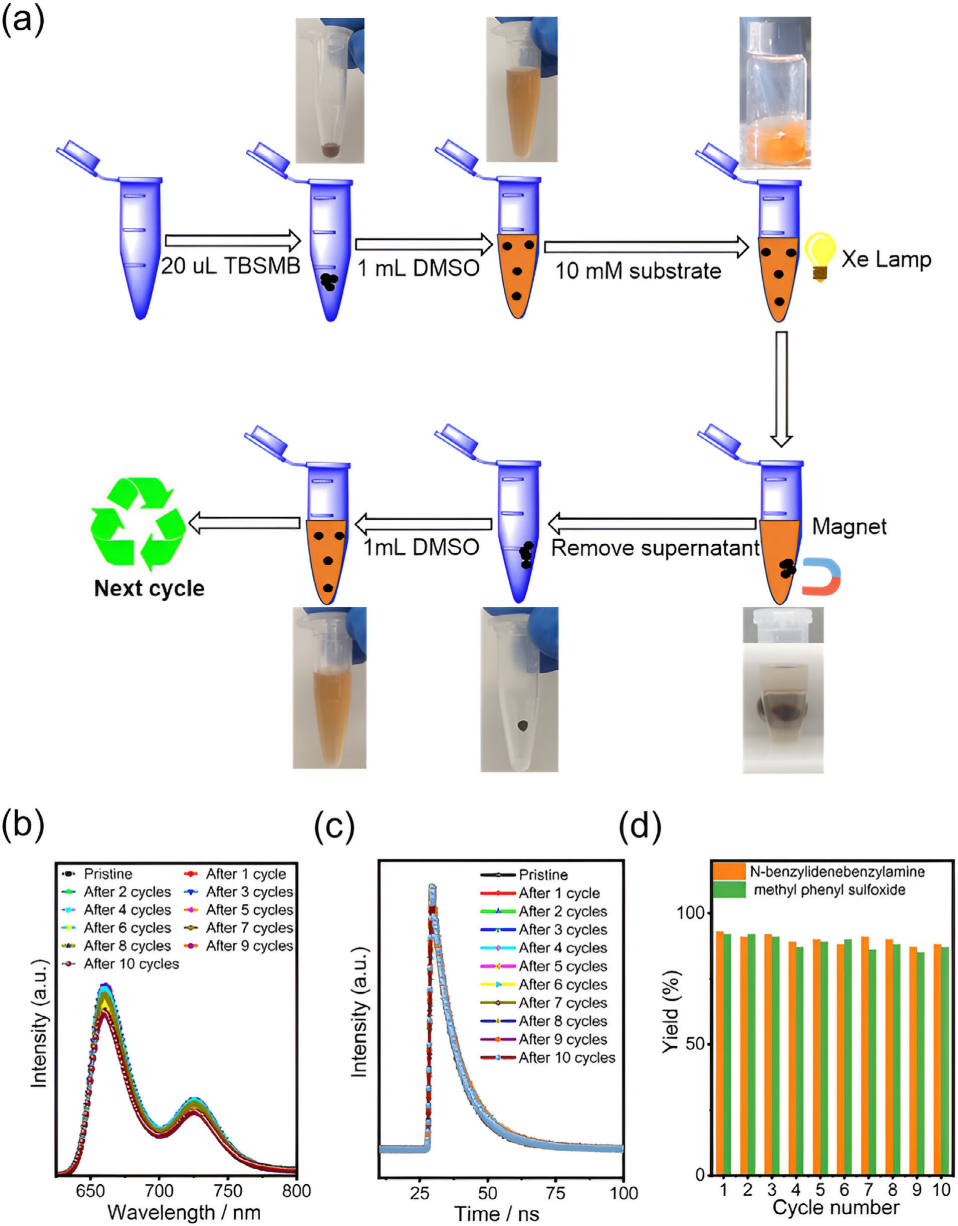
a) Schematic illustration of the recycling process; b) Fluorescence emission spectra and c) corresponding fluorescence lifetimes of pristine and recycled TBSMB; d) Yields of N‐benzylidenebenzylamine and methyl phenyl sulfoxide over 10 test cycles.

As mentioned earlier, efficient catalyst recovery for reuse purposes and stable catalytic activities are crucial for the practical applications of a heterogeneous photocatalytic system. Cyclic photocatalytic experiments of TBSMB were conducted following the procedures: the as‐prepared TBSMB (20 µL) was first dispersed well in 1 mL DMSO containing 0.01 m substrate; after the photocatalytic reaction, TBSMB was concentrated onto the vial wall with an external magnet and the supernatant solution was extracted; the collected TMSMB was then washed with DMSO several times before being re‐dispersed in substrate‐containing DMSO for the next round of photocatalytic reaction. It was observed even after 10 cycles, TBSMB still dispersed perfectly in DMSO, forming a transparent suspension with no visible precipitation, the mass loss of the recovered catalyst was as low as less than 0.1 mg (**Figure** **7**a).

Moreover, the fluorescence emission spectra of TBSMB in DMSO remained almost unchanged after 10 test cycles (Figure 7b), with minimal variation of the fluorescence lifetime of 10 ns (Figure 7c; Figures S21–S30, Table S3, Supporting Information), demonstrating the robustness of the as‐synthesized catalyst. More importantly, both the oxidative coupling of benzylamine and the oxidation of thioanisole proceeded efficiently with TBSMB throughout 10 test cycles with yields of 87%–93% for the former reaction and 87%–92% for the latter reaction (Figure 7d), further verifying the impressive chemical stability of the catalyst. The stability of TMSMB was further evaluated in various solvents (DMF, DMSO, acetonitrile, acetone, ethanol, and methanol) and pH (4–9; extreme acidic/basic conditions were excluded to avoid streptavidin conformational instability^[^ ^41^ ^]^) conditions with fluorescence emission spectroscopy and lifetime measurements (Figure S31, Supporting Information). The fluorescence spectra revealed minimal emission peak shifts aroused from the solvent effect (654–660 nm) and pH effect (656–660 nm). However, significant intensity reductions occurred in acetonitrile, ethanol, and at pH 9. Lifetime analysis showed negligible influence, with values narrowly distributed between 10.26 and 12.04 ns for most solvents. Acetonitrile was a notable exception, exhibiting a marked lifetime decrease to 6.45 ns. Similarly, pH variations (4–9) had limited impact: lifetimes remained stable at 10.04–11.09 ns under neutral/acidic conditions (pH 4–8) but decreased slightly to 9.01 ns at pH 9, consistent with the observed spectral intensity decline. These results demonstrate that TBSMB retained robust photophysical stability across diverse solvents and pH environments, with minor deviations observed only under specific conditions (acetonitrile, ethanol, and pH 9). Consequently, with the demonstrated exceptional recovering efficiency and catalyst stability, the TBSMB promised great potential in delivering acceptable photocatalytic activities for practical purposes in the future.

## 3 Conclusion

We have successfully developed a universal approach to construct exceedingly efficient and recyclable photocatalysts through tightly grafting chromophores onto commercial magnetic beads. The biotinylation of the four representative chromophore groups, including porphyrin, coumarin, anthraquinone, and pyrene, was found to hardly alter their photophysical properties. A pilot heterogeneous photocatalyst based on TAPP exhibited outstanding activities for the oxidative coupling of 24 benzylamine derivatives to imines and the oxidation of 15 thioanisole derivatives to sulfoxides, achieving product yield of as high as 99% and TON of as large as 10 000. The combination of experimental and theoretical studies unveiled that the two types of photocatalytic reactions took place with the mediation of photogenerated •O_2_⁻ and ^1^O_2_. Importantly, the catalytic system exhibited exceptional stability and recyclability, maintaining minute mass loss and high catalytic activities even after 10 test cycles. This work opens up new possibilities for pushing forward practical applications of solar‐driven synthesis of important organic chemical compounds.

## 4 Experimental Section

### Materials

Experimental reagents (Aladdin) and solvents were used without further purification. Silica gel 60 (230–400 mesh) was used in column chromatography. The streptavidin magnetic beads (BeyoMag Streptavidin Magnetic Beads, commodity number P2151) were purchased from Beyotime Biotechnology company. According to the kit instructions 1 mL magnetic beads could bind 20 µg biotin, which means the molar value of biotin would be 2 × 10^−8^ mol when 100 µL was extracted The molar ratio of porphyrin/biotin was 0.25. Thus, the molar value of porphyrin (TAPP‐Biotin) was 5 × 10^−9^ mol when 100 µL magnetic beads were extracted.

### Synthesis

The synthetic methods of preparing dyads TAPP‐biotin, AAQ‐biotin, AP‐biotin, and AMC‐biotin were quite similar. General procedures were: dissolving 0.3 µmol chromophore in 3 mL DMF and stirring for 5 min, then making a 5 mL‐DMF solution containing 0.9 µmol d‐biotin, 1 µmol benzotriazol‐1‐yl‐oxytripyrrolidino‐phosphonium hexafluorophosphate (PyBOP) and 1 µmol N, N‐diisopropylethylamine (DIPEA). The substrate solution was added dropwise by the mixed solution within 5 min at 45 °C in the round flask. The reaction was kept for less than 6 h until the starting material point disappeared shown in TLC. Evaporate the reaction mixture and run a quick flash silica column using the mixture of ethyl acetate and hexane, to obtain the pure product. Applying NMR and MS measurements to conduct the characterizations.

### Synthesis—TAPP‐Biotin

^1^H‐NMR (600 MHz, d‐DMSO): δ = 8.87 (s, 8H, pyrrole‐H), 8.12 (d, *J* = 8.4 Hz, 8H, phenyl‐H), 8.06 (d, *J* = 8.4 Hz, 8H, phenyl‐H), 6.49‐1.61 (64H, d‐biotin), ‐2.89 (s, 2H, pyrrole inner‐H). ESI‐MS (negative mode): Chemical Formula: C_84_H_90_N_16_O_8_S_4_; calculated [M+Na]: 1601.7898; observed: 1601.5883. Isolated yield: 86%.

### Synthesis—AAQ‐Biotin

^1^H‐NMR (600 MHz, MeOD): δ = 7.88 (d, *J* = 8.5 Hz, 2H, phenyl‐H), 7.74 (d, *J* = 8.3 Hz, 2H, phenyl‐H), 7.56 (m, 3H, phenyl‐H), 7.49 (t, *J* = 7.5 Hz, 3H), 4.49 (dd, *J* = 7.8, 4.9 Hz, 1H), 4.31 (dd, *J* = 7.8, 4.5 Hz, 1H), 3.72 (m, 6H), 2.31 (d, *J* = 7.4 Hz, 2H), 1.46 (d, *J* = 6.8 Hz, 2H), 1.32 (d, *J* = 6.5 Hz, 3H). ESI‐MS (negative mode): Chemical Formula: C_24_H_23_N_3_O_4_S; calculated [M]: 449.14, observed [M‐H]: 448.13. Isolated yield: 76%.

### Synthesis—AP‐Biotin

^1^H‐NMR (600 MHz, MeOD): δ = 8.17 (s, 1H, phenyl‐H), 8.06 (d, *J* = 8.3 Hz, 2H, phenyl‐H), 7.95 (d, *J* = 8.2 Hz, 2H, phenyl‐H), 7.75 (m, 5H), 3.58 (s, 2H, NH), 3.39 (d, *J* = 6.9 Hz, 4H, ‐CH2), 2.21 (s, 2H, ‐CH2), 2.14 (s, 2H), 2.09 (s, 3H), 1.51 (d, *J* = 6.3 Hz, 2H, CH). ESI‐MS (negative mode): Chemical Formula: C_26_H_25_N_3_O_2_S; calculated [M]: 443.17, observed [M‐H]: 442.16. ESI‐MS (positive mode): calculated [M]: 443.17, observed [M+H]: 444.17. Isolated yield: 82%.

### Synthesis—AMC‐Biotin

^1^H‐NMR (600 MHz, CDCl_3_): δ = 9.07 (s, 2H, NH‐H in d‐biotin), 7.30 (d, *J* = 8.5 Hz, 4H, phenyl‐H), 6.56 (dd, *J* = 8.5, 2.2 Hz, 3H), 6.51 (d, *J* = 2.2 Hz, 3H), 5.93 (s, 3H, ‐CH3), 3.65 (m, 2H), 1.23 (s, 4H, ‐CH2), 1.18(s, 2H). ESI‐MS (positive mode): Chemical Formula: C_20_H_23_N_3_O_4_S; calculated [M]: 401.14, observed [M+H]: 402.14. Isolated yield: 78%.

### Physical Methods

^1^H, ^19^F NMR spectra were recorded on a Bruker Avance III 600 spectrometer equipped with a 5 mm, automated tuning, and matching broadband probe (BBFO) with z‐gradients. Chemical shifts were reported in ppm relative to the residual hydrogen atoms in the deuterated solvent (d‐DMSO (δ = 2.50 ppm), CDCl_3_ (δ = 7.26 ppm)). Absorption spectra of the samples were measured on an Agilent 8454 spectrophotometer. Mass spectra for the compounds were performed on a Bruker MALDI‐TOF mass spectrometer. GC‐MS (Agilent 1260) was applied to determine the conversion yields of 24 substrates of amine and 15 substrates of sulfide.

### Spectroscopy

Steady‐state emission spectra were recorded on a Cary Eclipse fluorimeter, at room temperature. The fluorescence lifetimes (τ) were measured by the combined spectrometer consisting of the EDINBURGH LIFESPEC II and the picosecond pulsed diode laser: EDINBURGH EPL‐365/405 in DMSO at room temperature.

### Scanning Electron Microscopy (SEM)

SEM images were taken with Hitachi SU8020 scanning electron microscopy. Energy Dispersive X‐ray Spectroscopy (EDS) mapping was based on HORIBA EMAX mics2. Speeding voltage is 5KV.

### EPR Assays for Detecting the ^1^O_2_ and O_2_^−^ Radical

EPR experiments were performed in a mixture of the 10 µm TAPP or TAPP‐biotin and 0.1 m DMPO or TEMP air‐saturated DMSO suspension in the dark or irradiated by 300 W xenon lamp (100 mW cm^−1^).

### UV–vis Method for Detecting the ^1^O_2_ and O_2_^−^ Radical

Using 1,3‐diphenylisobenzofuran (DPBF) and nitro blue tetrazolium (NBT) as scavengers for ^1^O_2_ and O_2_^−^ radical. 3 mL DMF solution containing 5 µm TAPP or TAPP‐biotin, and 50 µm DPBF or NBT, irradiation by Xe lamp, and monitor the absorption peaks of DPBF and NBT with irradiation for 180 s or 90 min, respectively.

### Singlet Oxygen Quantum Yield Determinations

The steady‐state luminescence of singlet oxygen was measured by using an FLS920 spectrofluorimeter (Edinburgh Instruments) with a wavelength ranges from 600 to 1700 nm. The FLS920 spectrofluorimeter was equipped with a TM300 excitation monochromator and a TM300 emission monochromator equipped with a NIR grating, and a Hamamatsu R5509‐72 supercooled photomultiplier tube at 193 K. The excitation wavelength was 550 nm. The singlet oxygen quantum yields Φ_Δ_ could be calculated by the comparative actinometry method.^[^ ^42^ ^]^ In the following equation, the Φ_Δ_ of TAPP in DMF is the standard sample, whose value is 0.26.^[^ ^40^ ^]^ I and Istd refer to the singlet oxygen emission intensities at the peaks for the tested samples and TAPP, respectively. A and A^std^ stand for the ground‐state absorbance of the tested samples and TAPP at the excited wavelength of 550 nm, respectively.

(1) $$\Phi \Delta =\Phi \Delta^{std}I\left(1-10^{-Astd}\right)/I_{std}\left(1-10^{-A}\right)$$

### DFT Calculations for S1 and T1

All the calculations were performed by using Gaussian 16 and Orca 5.0.2 suite of programs.^[^ ^43^, ^44^ ^]^ Ground state geometries were calculated by B3LYP‐D3 functional. The excited state calculation was simulated by TD‐DFT. The rate constant of Intersystem Crossing (ISC) and reverse Intersystem Crossing (rISC) were evaluated by using Marcus theory.^[^ ^45^ ^]^

### DFT Calculations for Reaction Mechanism

All computational studies were conducted using density functional theory (DFT) as implemented in the Gaussian 16 quantum chemistry software suite.^[^ ^43^ ^]^ Visualization of the computational results was carried out with the help of GaussView 6.^[^ ^46^ ^]^ Geometry optimizations were performed using the B3LYP‐D3functional combined with the LanL2DZ basis set.^[^ ^47^, ^48^, ^49^ ^]^ Excited‐state properties were calculated using time‐dependent DFT (TD‐DFT) with the camB3LYP functional, including empirical dispersion corrections. To ensure the nature of each optimized structure, harmonic frequency analyses were conducted. Structures were classified as minima if no negative eigenvalues were found in the Hessian matrix and as transition states if exactly one negative eigenvalue was present. Thermal and entropy corrections to the total energy were obtained from the thermochemical data in the output files, evaluated at 298 K and 1 atm pressure. All calculations were carried out in the solvent phase (acetonitrile) to ensure consistency with experimental conditions, as the experiments were also performed in solution.

## Conflict of Interest

The authors declare no conflict of interest.

## Supporting information



Supporting Information

## Data Availability

The data that support the findings of this study are available from the corresponding author upon reasonable request.
