# Supplementary material for: A Universal Approach to Anchoring Chromophores onto Magnetic Scaffold for Achieving Easily Recyclable Heterogeneous Photocatalytic Systems
Source: Adv Sci (Weinh). 2025 Mar 26;12(26):2502342. doi: 10.1002/advs.202502342 (PMC12245082; doi:10.1002/advs.202502342)
Supplement: Supplementary file 1 — Supporting Information [file ADVS-12-2502342-s001.docx]

Supporting Information

**A Universal Approach to Anchoring Chromophores onto Magnetic Scaffold for Achieving Easily Recyclable Heterogeneous Photocatalytic Systems**

*Xuan Zhan, Yikun Wang, Chenyang Sun, Yuchen Fang, Lihua Huang, Zakir Ullah,* Qiang Chen, Xuejing Wang, Zheng Xing,* and Gangfeng Ouyang**

X. Zhan, Y. Wang, C. Sun, Y. Fang, L. Huang, Q. Chen, Z. Xing and G. Ouyang: School of Chemical Engineering and Technology, Sun Yat-sen University, Zhuhai 519082, China

E-mail: xingzh7@mail.sysu.edu.cn; cesoygf@mail.sysu.edu.cn

Z. Ullah: Institut de Ciència de Materials de Barcelona (ICMAB–CSIC), Consejo Superior de Investigaciones Científicas, Campus Universitari de Bellaterra, Cerdanyola del Vallès 08193, Spain

E-mail: azazay1@gmail.com

X. Wang: Macao Institute of Materials Science and Engineering (MIMSE), Faculty of Innovation Engineering, Macau University of Science and Technology, Taipa, Macao 999078, China

**Content**

| Scheme S1 | S3 |
| --- | --- |
| Figure S1: The ^1^H-NMR spectra of the TAPP-biotin dyad in d-DMSO | S3 |
| Figure S2: MALDI-TOF spectrum of the TAPP-biotin dyad in MeOH | S4 |
| Figure S3-5: ^1^H-NMR and MS characterization of dyads AAQ-biotin, AP-biotin, AMC-biotin | S4-5 |
| Figure S6-9: UV-vis spectra, Fluorescence emission spectra and lifetimes of all chromophore and their corresponding dyads | S6-7 |
| Table S1: Biotinylation effect on UV-vis peaks and fluorescence emission peaks | S7 |
| Figure S10: Scheme of preparing TBSMB | S8 |
| Figure S11: SEM image of TBSMB | S8 |
| Figure S12: SEM image of commercial streptavidin magnetic beads | S9 |
| Figure S13. SEM image and EDS mapping of commercial streptavidin magnetic beads | S9 |
| Figure S14: Element analysis for both two types of magnetic beads | S10 |
| Figure S15: Measured UV-vis for investigating aggregation behavior | S10 |
| Table S2: Catalytic performance comparison | S11 |
| Figure S16: Wavelength-dependent photocatalytic performance and time courses | S12 |
| Figure S17: Wavelength-dependent photocatalytic performance | S13 |
| Figure S18: Time courses of two oxidation reactions | S13 |
| Figure S19: UV-vis method to assess the ^1^O_2_ generation of TAPP and the TAPP-biotin dyad | S14 |
| Figure S20: UV-vis method to assess the O_2_^-^ generation of TAPP and the TAPP-biotin dyad | S14 |
| Table S3: Summarized fitting results of 10-cycle fluorescence lifetimes | S15 |
| Figure S21-30: Fluorescence lifetime data for 10 test cycles | S16-19 |
| Figure S31: Solvent and pH effect on photocatalytic performance | S19 |
| Figure S32-70: GC-MS analysis for two types of photocatalytic reactions | S20-32 |
| Optimized cartesian coordinates | S33-47 |

**
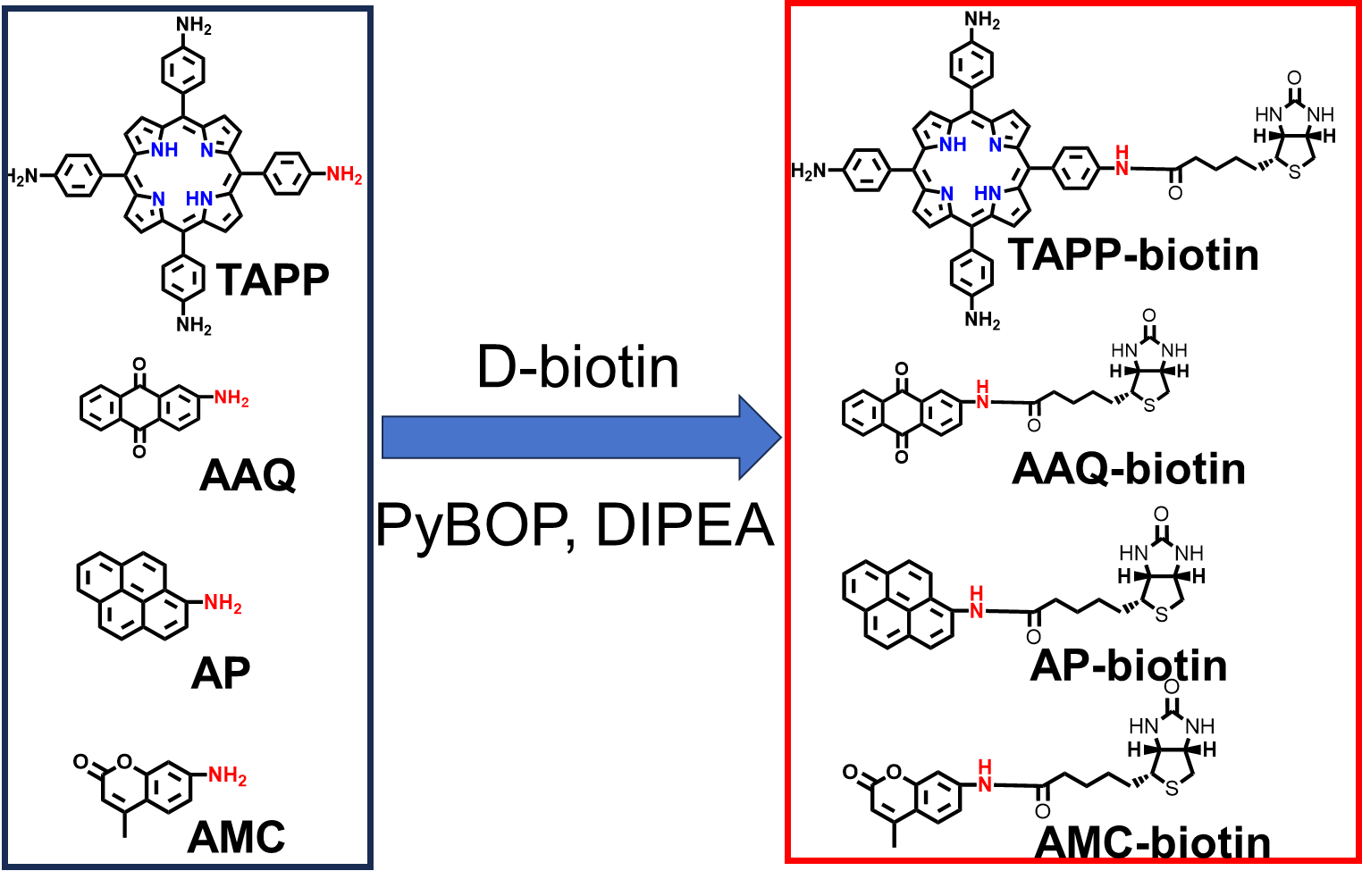
**

**Scheme S1.** Chemical structures of chromophores and corresponding prepared chromophore-biotin dyads TAPP-biotin, AAQ-biotin, AP-biotin and AMC-biotin.

**
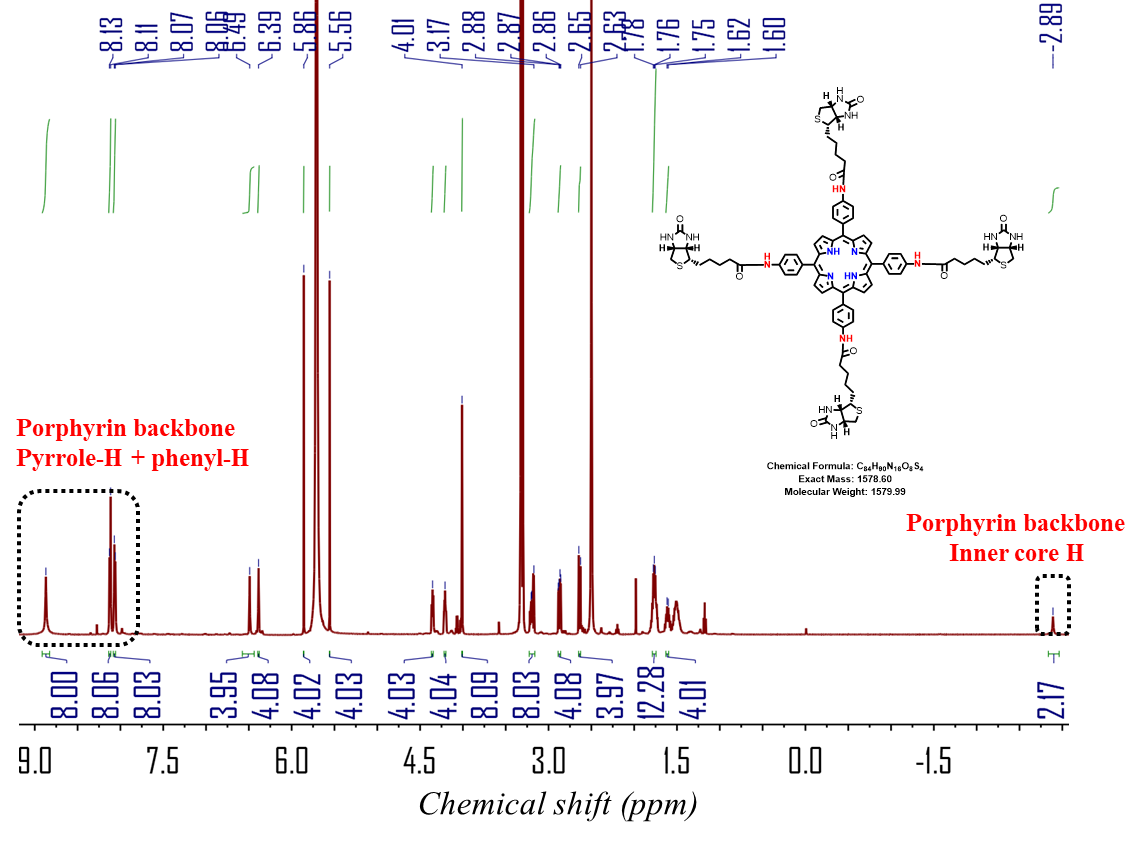
**

**Figure S1.** The ^1^H-NMR spectra of the TAPP-biotin dyad in d-DMSO.

**
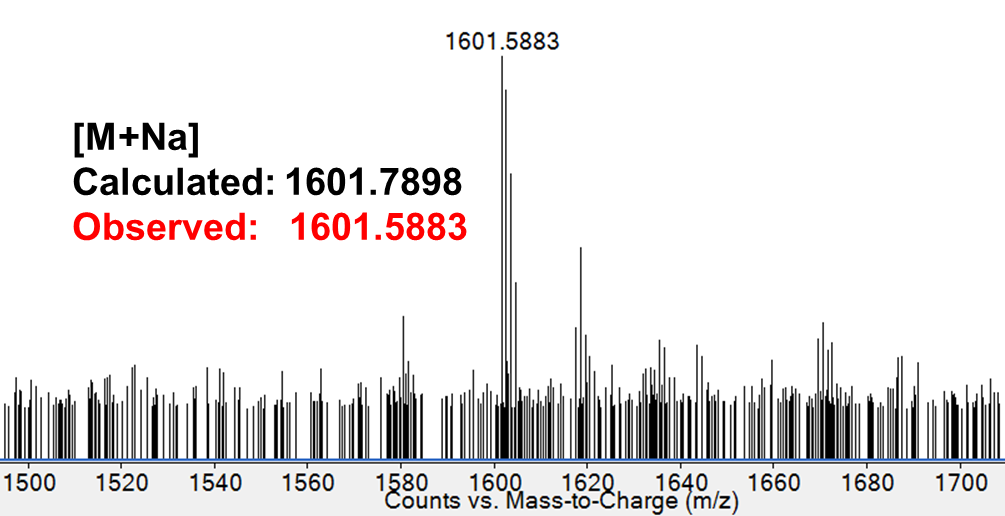
**

**Figure S2.** MALDI-TOF spectrum of the TAPP-biotin dyad in methanol.

**
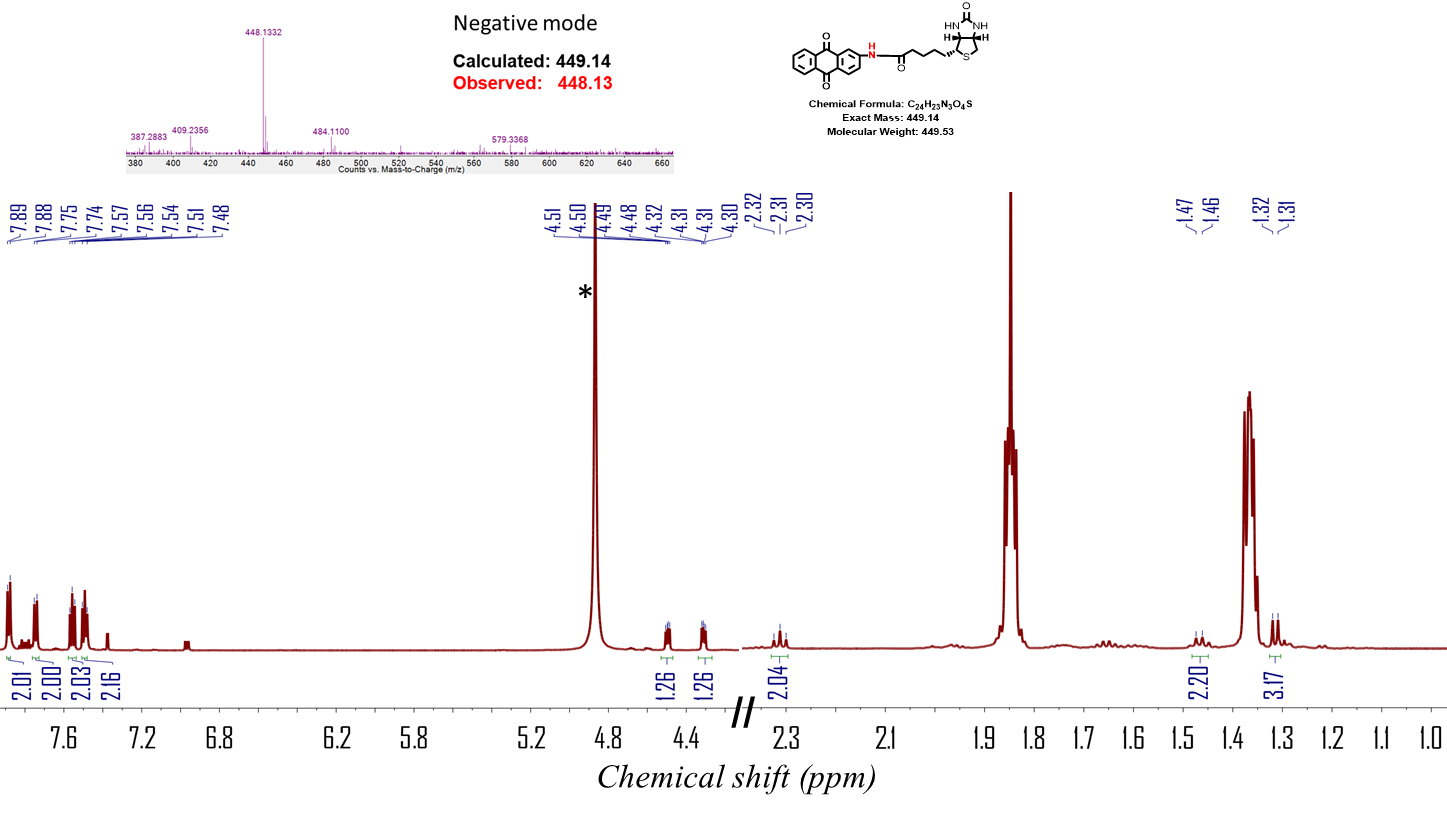
**

**Figure S3.** The ^1^H-NMR spectra of the AAQ-biotin dyad in d-DMSO and its MS data. * Solvent residue from ethyl acetate and H_2_O.

**
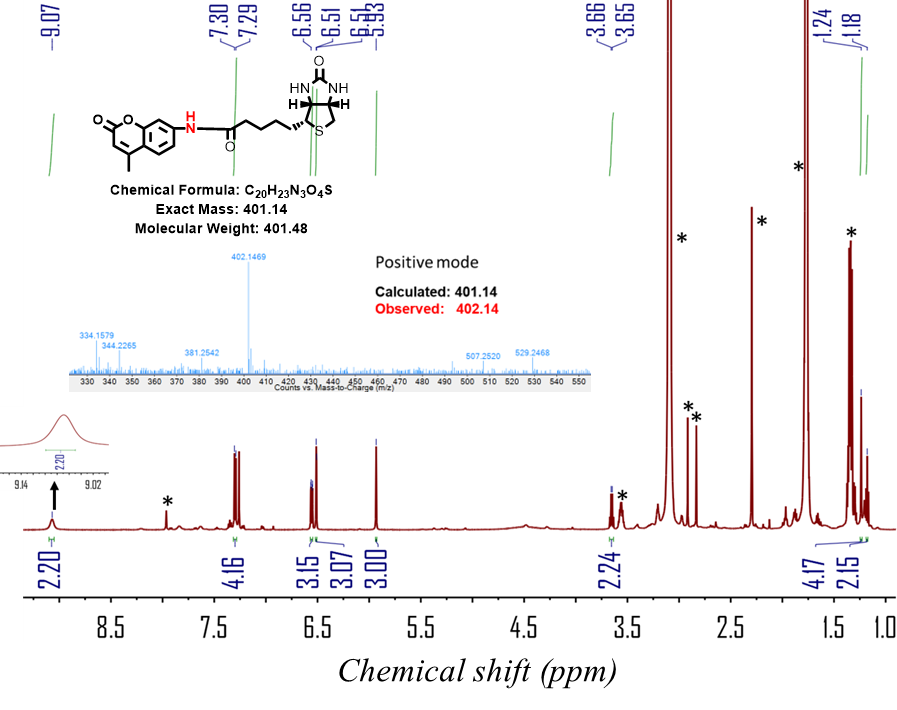
**

**Figure S4.** The ^1^H-NMR spectra of the AMC-biotin dyad in d-DMSO and its MS data. * Solvent residue from DMF, ethyl acetate, ethanol and DIPEA.

**
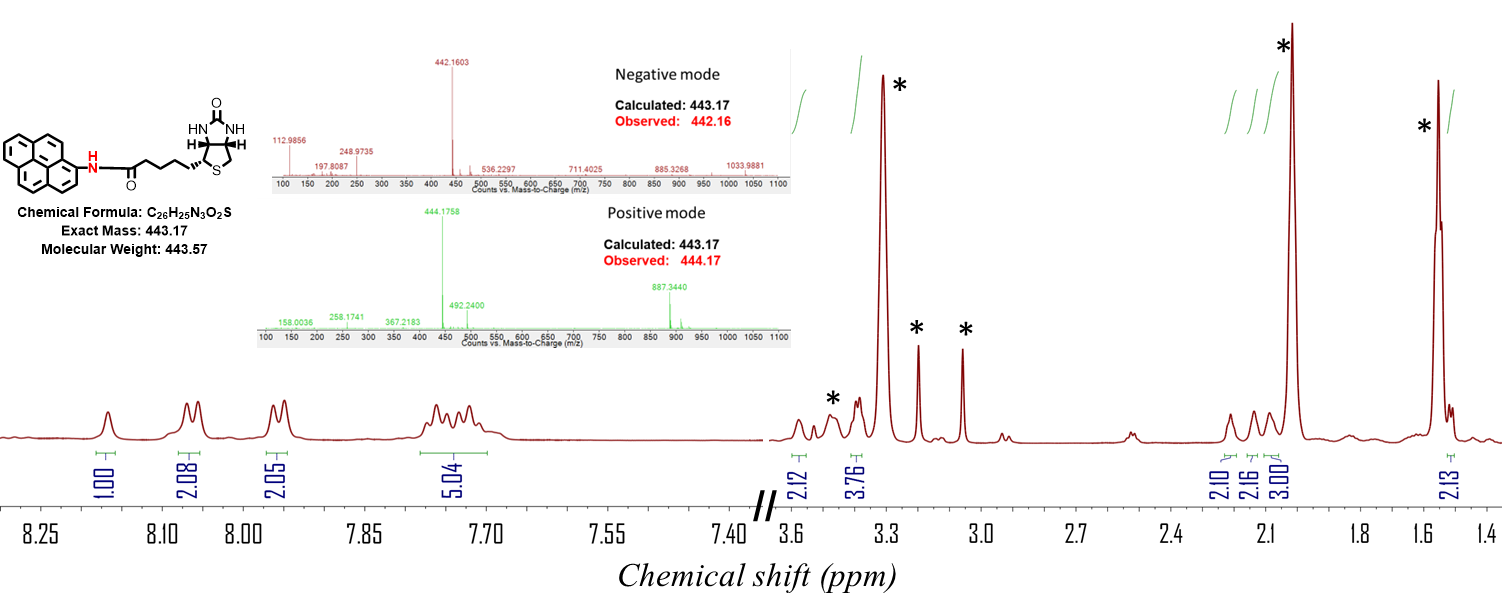
**

**Figure S5.** The ^1^H-NMR spectra of the AP-biotin dyad in d-DMSO and its MS data. * Solvent residue from MeOD, ethyl acetate and methanol.

**
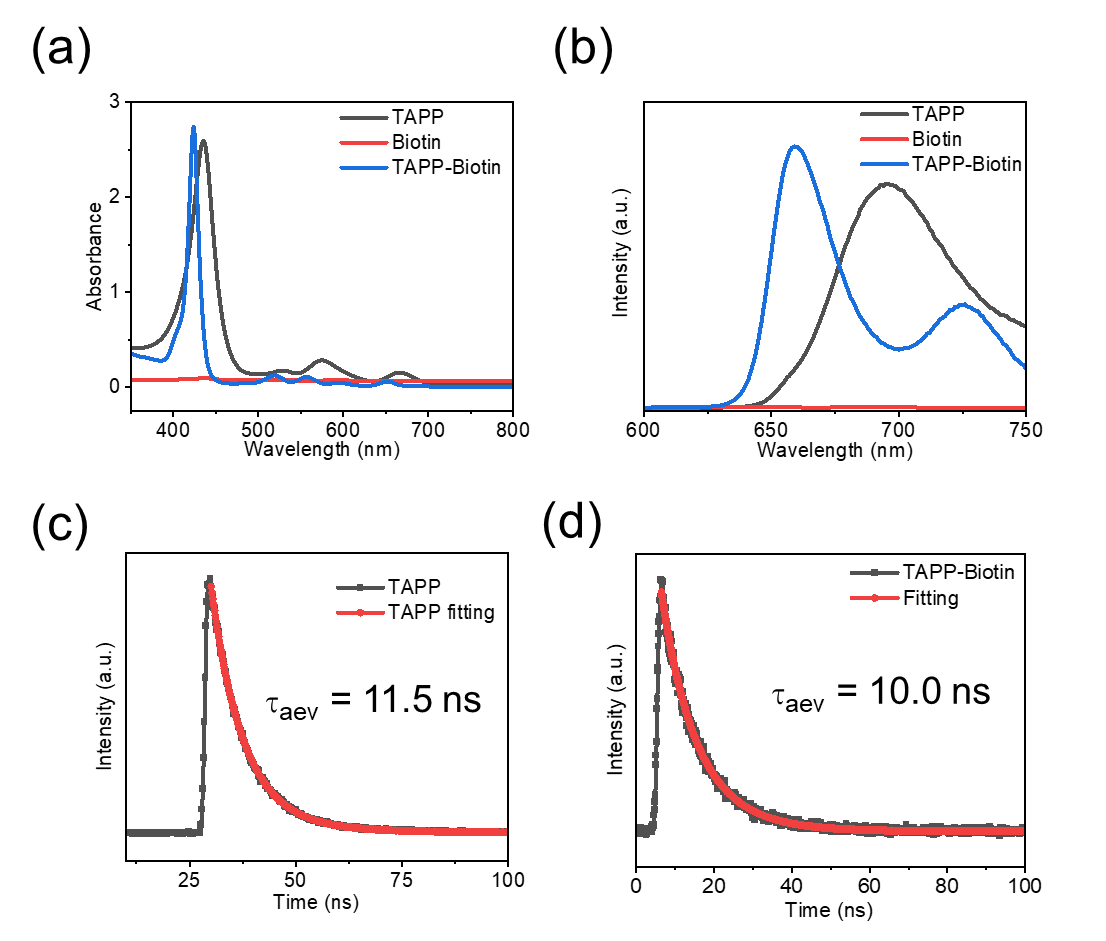
**

**Figure S6.** (a) UV-vis spectra of TAPP, biotin and the TAPP-biotin dyad in DMF; (b) Fluorescence emission spectra of TAPP, biotin and the TAPP-biotin dyad in DMF; time-resolved fluorescence lifetimes of TAPP and the TAPP-biotin dyad in DMF. All measured concentrations are set at 10^-5^ M.


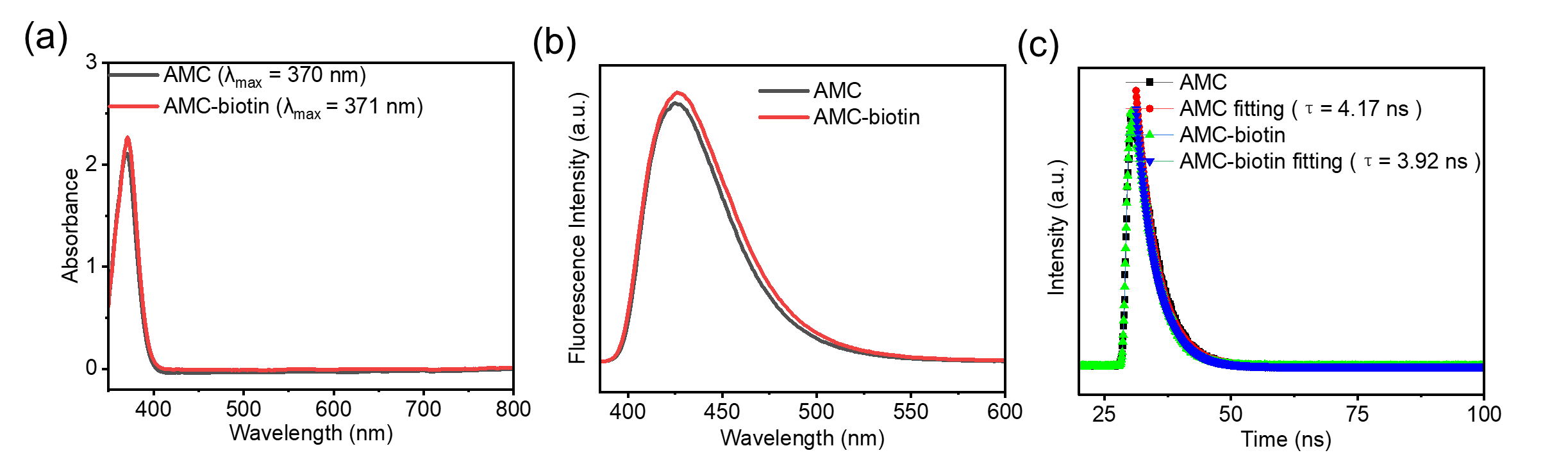


**Figure S7.** UV-vis (a), fluorescence emission spectra (b) and time-resolved fluorescence lifetimes (c) of AMC, the AMC-biotin dyad.


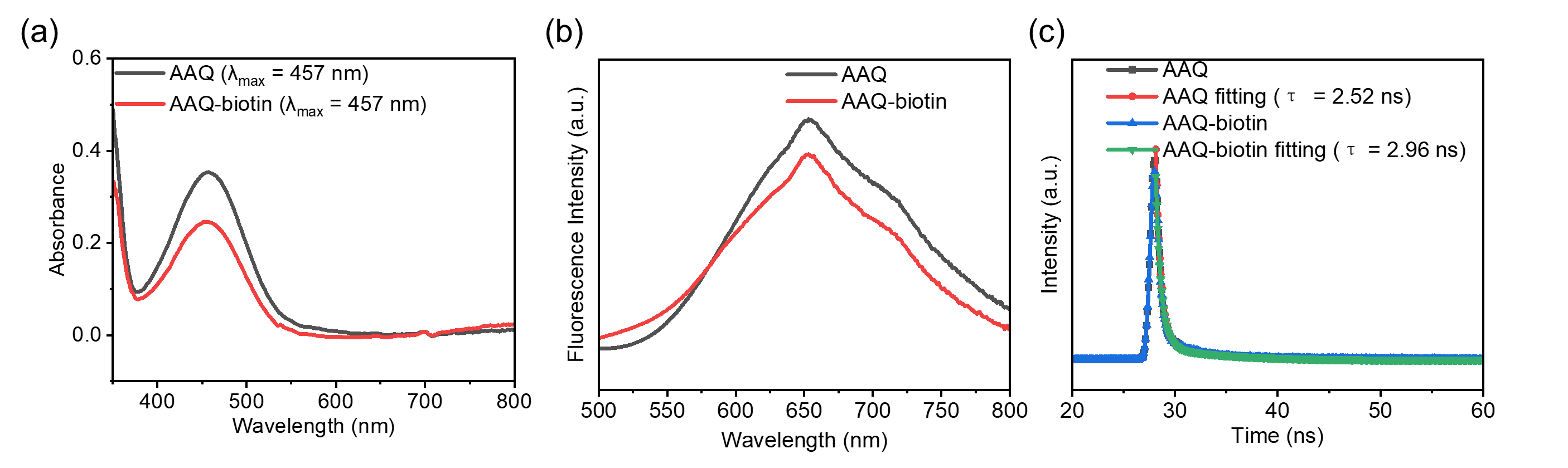


**Figure S8.** UV-vis (a), fluorescence emission spectra (b) and time-resolved fluorescence lifetimes (c) of AAQ, the AAQ-biotin dyad.


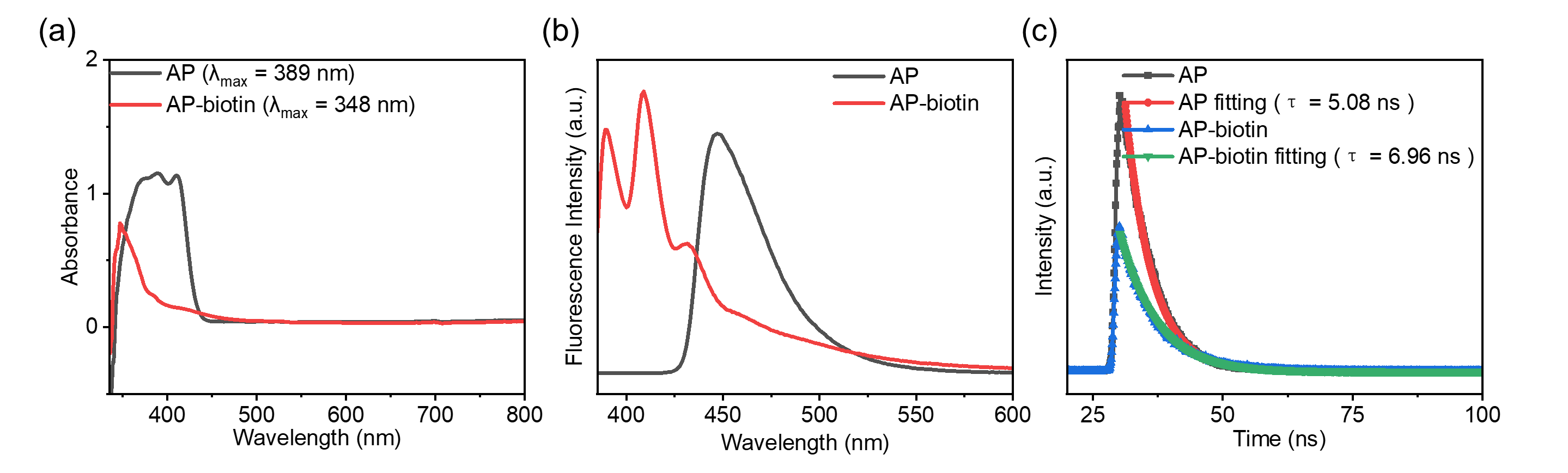


**Figure S9.** UV-vis (a), fluorescence emission spectra (b) and time-resolved fluorescence lifetimes (c) of AP, the AP-biotin dyad.


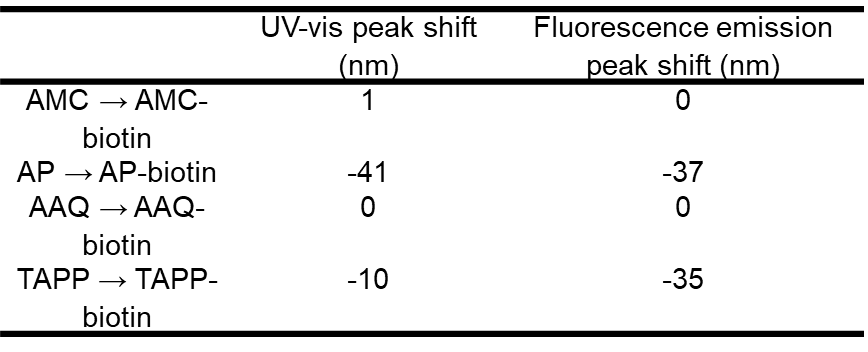


**Table S1.** Biotinylation effect on UV-vis peaks and fluorescence emission peaks on four chromophores.

**
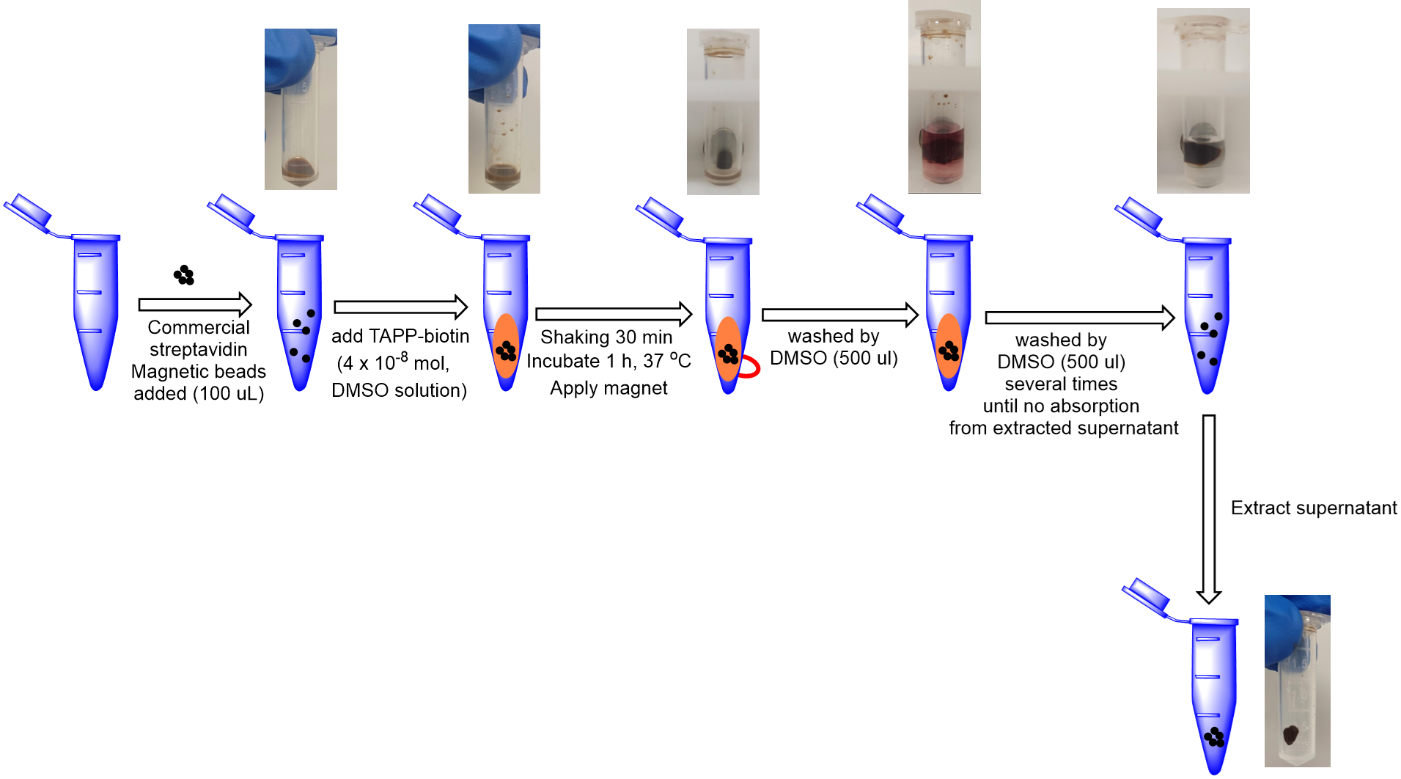
**

**Figure S10.** Illustrated scheme of the preparation of TMSMB.


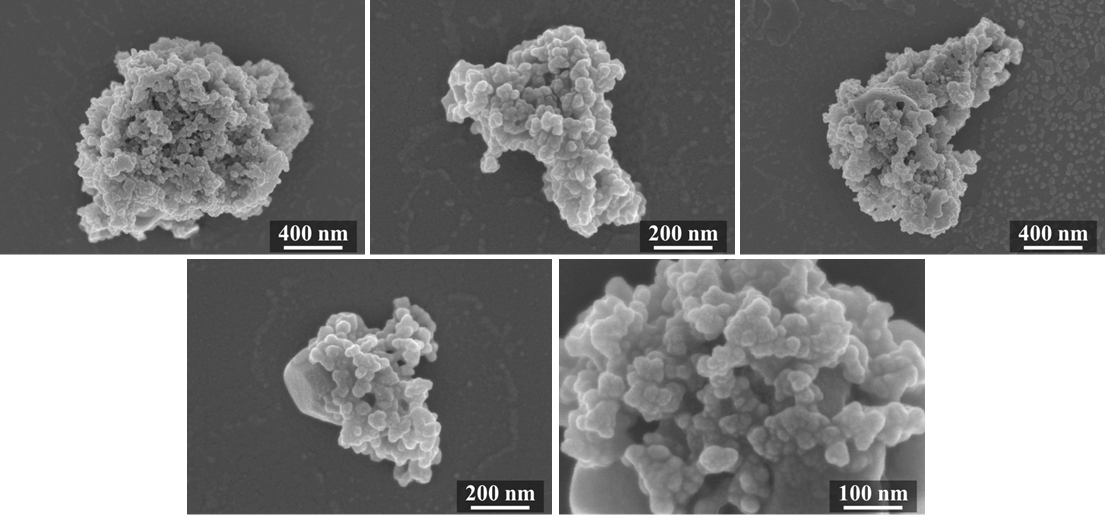


**Figure S11.** SEM image of as-prepared TMSMB.


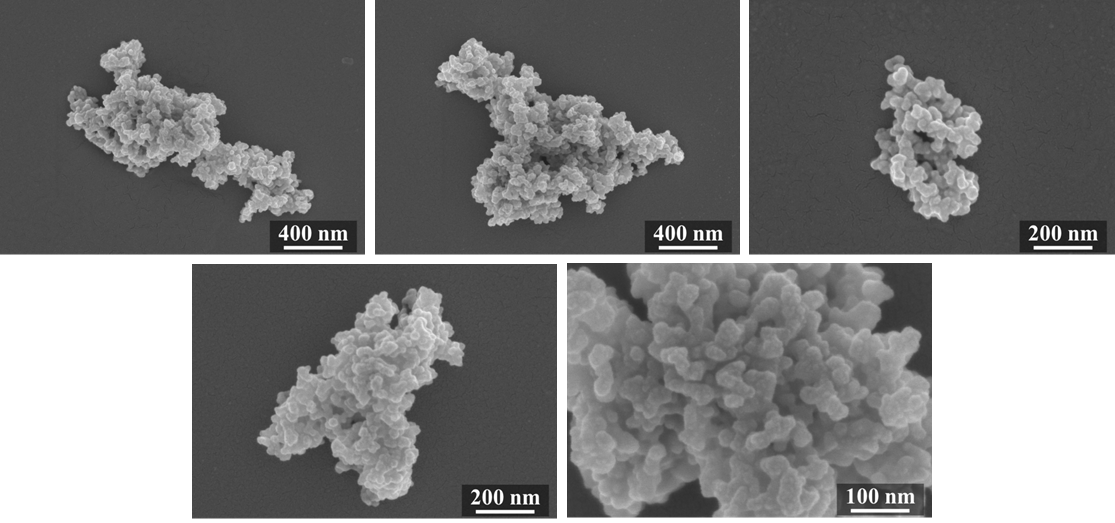


**Figure S12.** SEM image of commercial streptavidin magnetic beads.


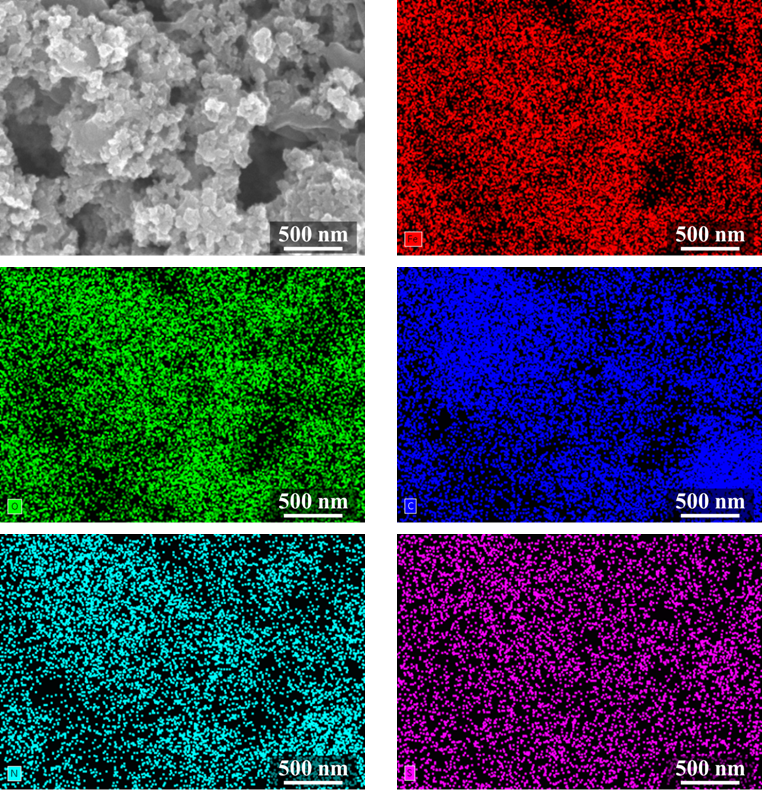


**Figure S13.** SEM image of commercial streptavidin magnetic beads and

corresponding elemental mapping images of Fe, O, C, N and S.


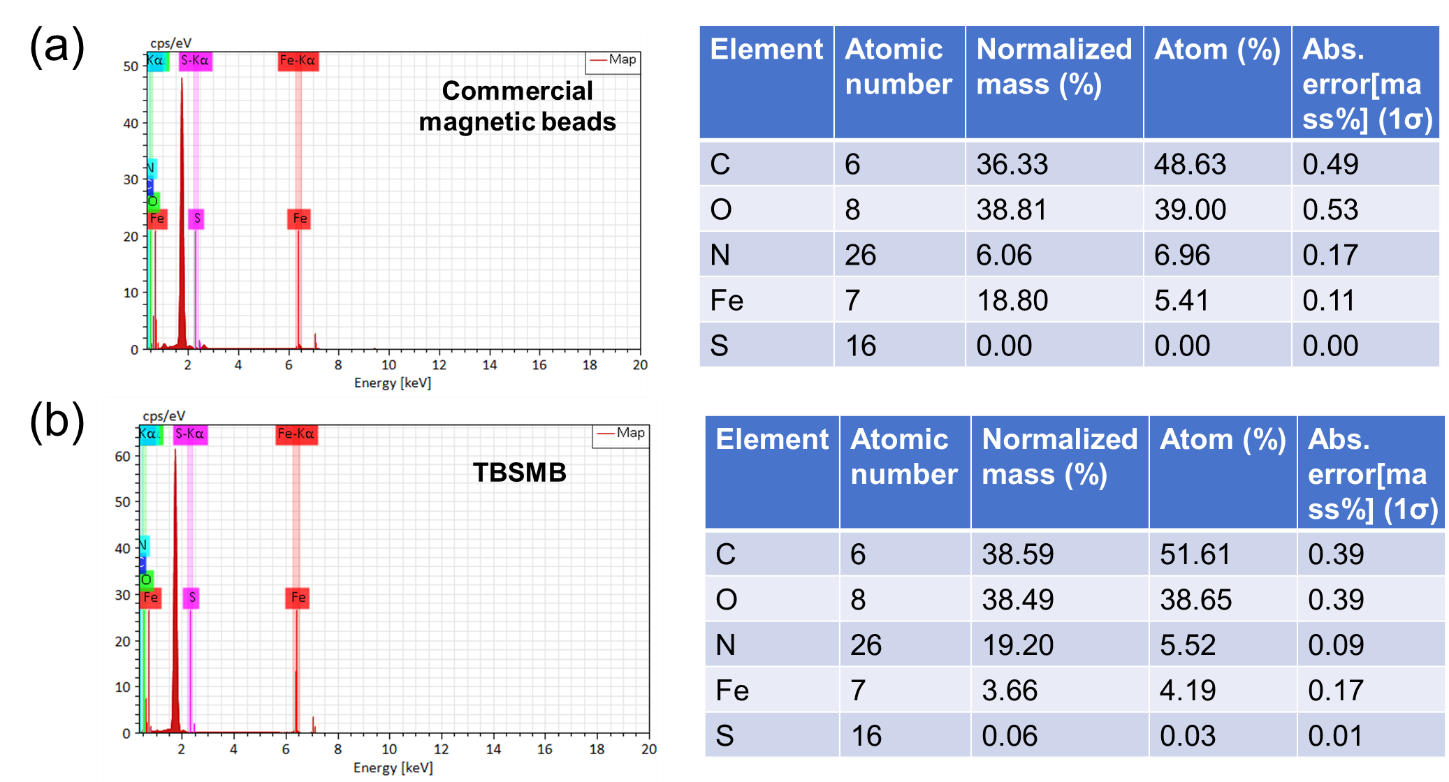


**Figure S14.** Element analysis for both TBSMB (a) and commercial streptavidin magnetic beads (b).

**
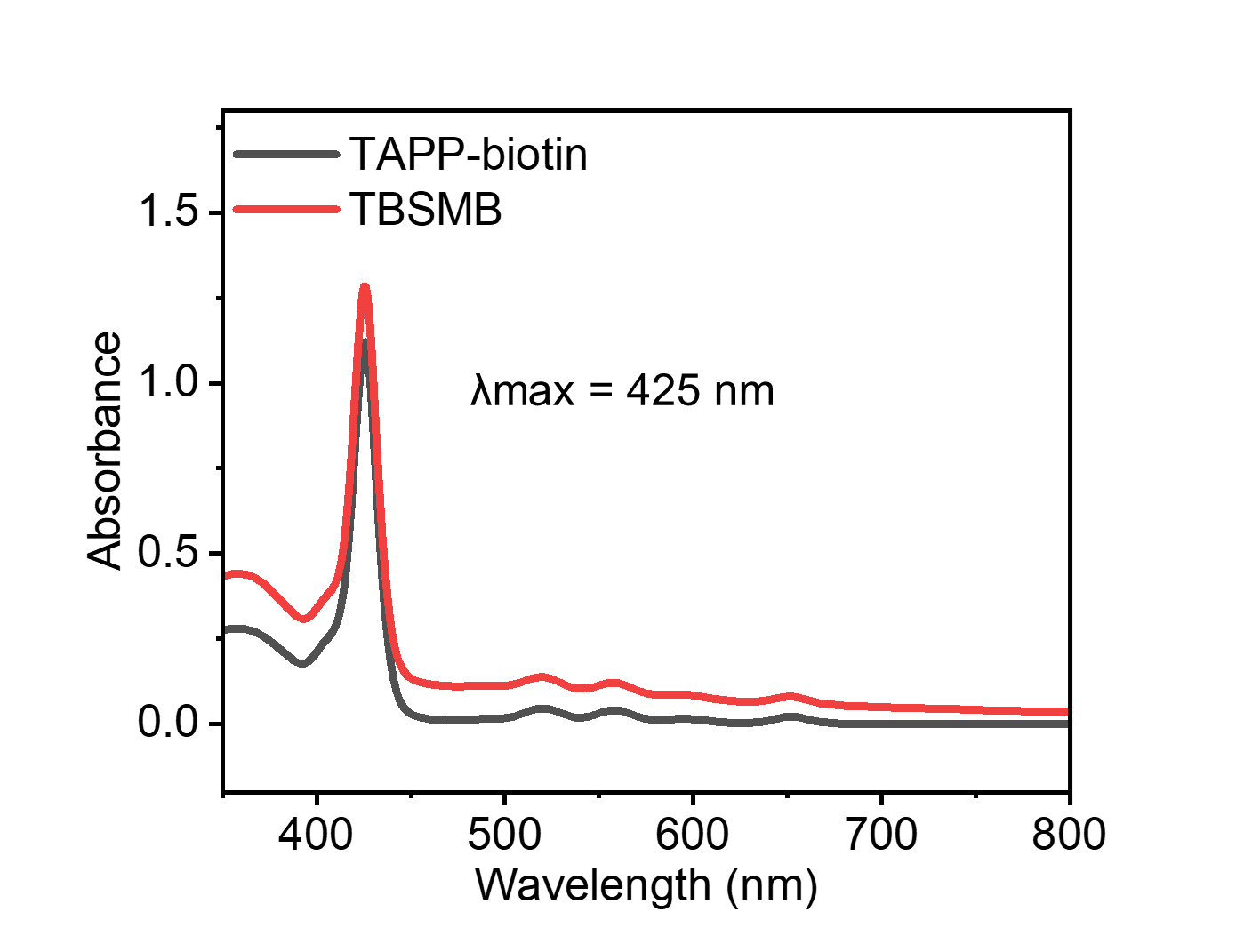
**

**Figure S15.** UV-vis spectra of 20 uM TAPP-biotin and TBSMB containing 20 uM TAPP-biotin in DMSO.

**
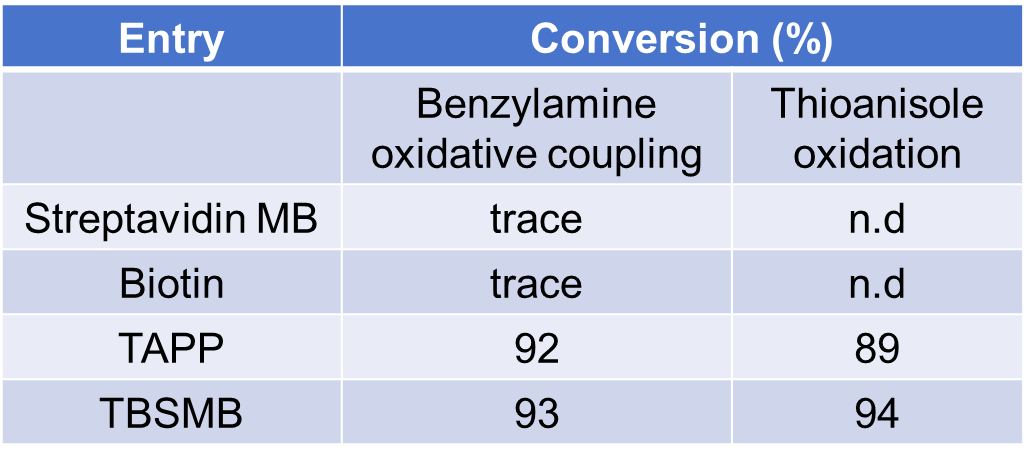
**

**Table S2.** Catalytic performance comparison among streptavidin magnetic beads, biotin, TAPP and TBSMB, with catalyst moles set to 5 × 10^-9^ in DMSO containing 0.1 M benzylamine or thioanisole, under irradiation for 3 hours (Xe lamp).


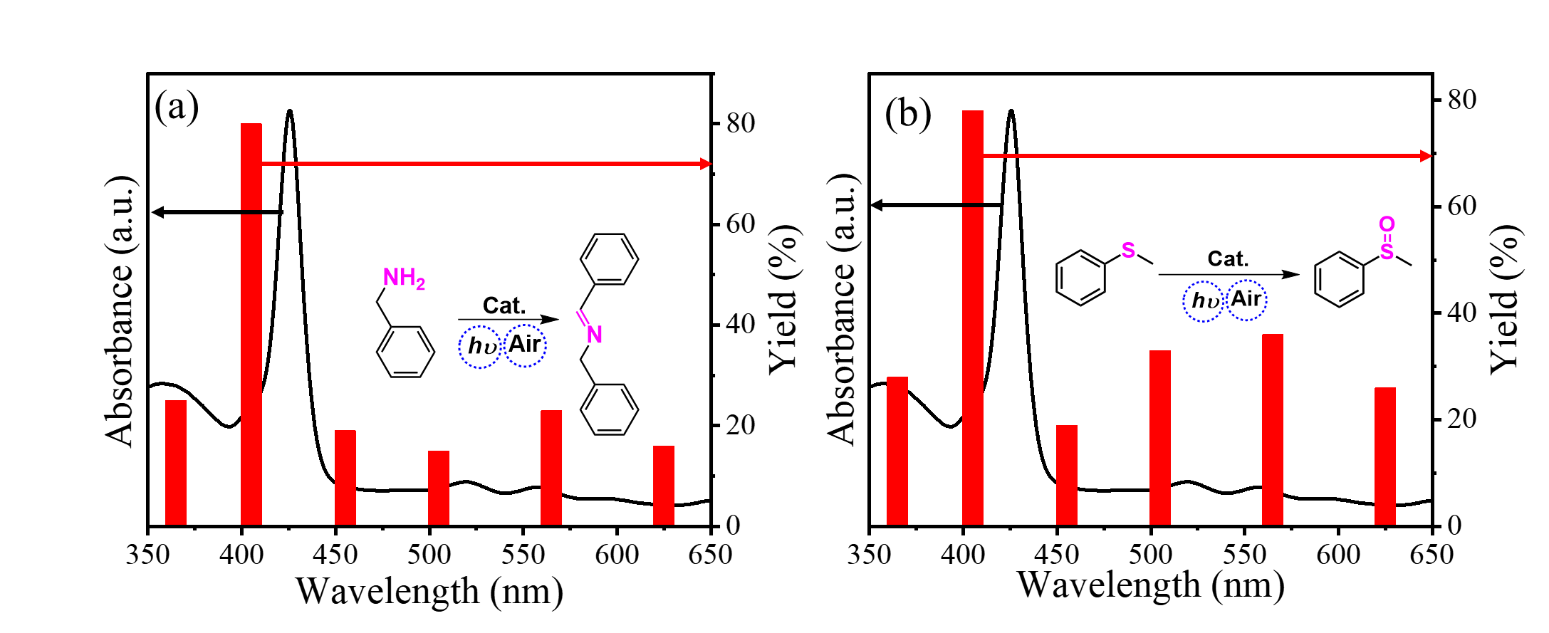


**Figure S16.** Wavelength-dependent photocatalytic performance (yield) for (a) the benzylamine oxidation and (b) the thioanisole oxidation under irradiation at 365, 405, 455, 505, 565 and 625 nm. The intensities of all applied light source were set to 20 mW.


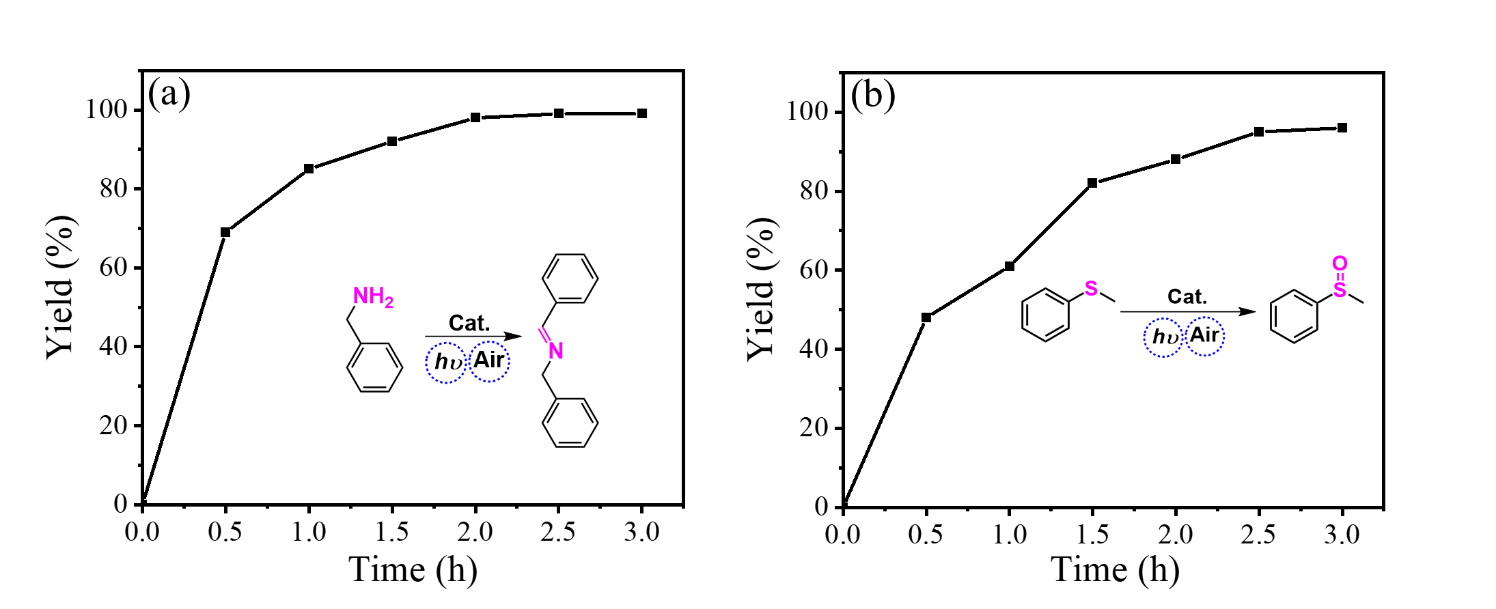


**Figure S17.** The time profiles of photocatalytic oxidation of (c) benzylamine and (d) thioanisole under Xe lamp irradiation conducted with TBSMB. Experimental condition:1mL DMSO containing 20 uL TBSMB and 0.01mM benzylamine or thioanisole was applied.


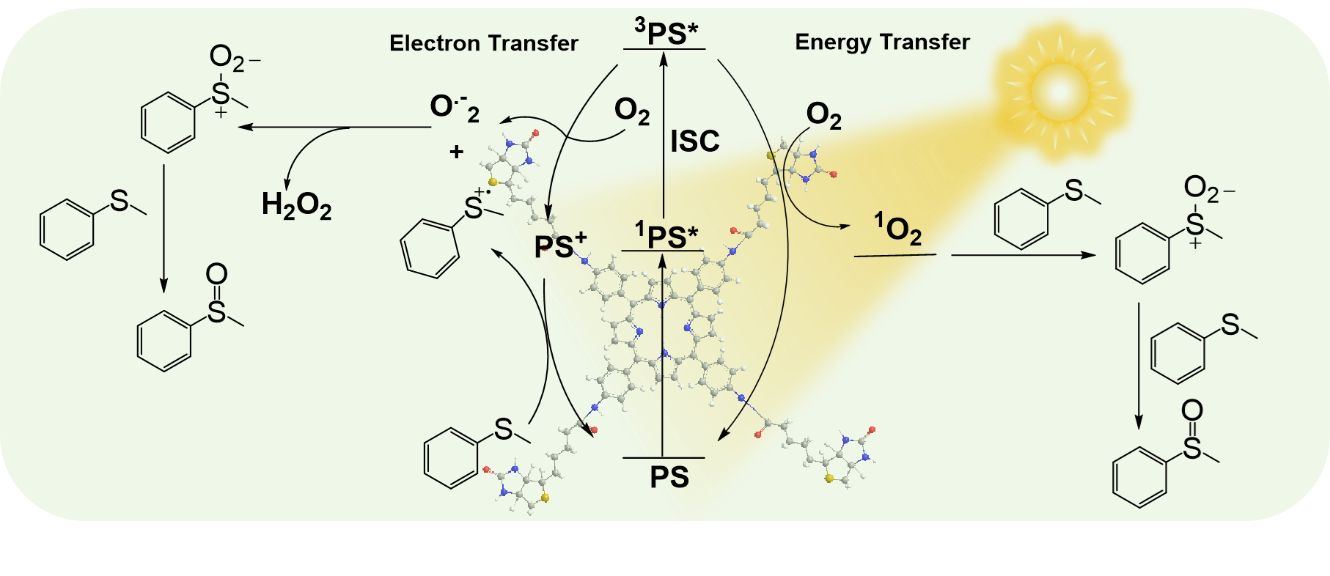


**Figure S18.** Proposed mechanism of the oxidation of thioanisole by using TBSMB as photocatalyst.

**
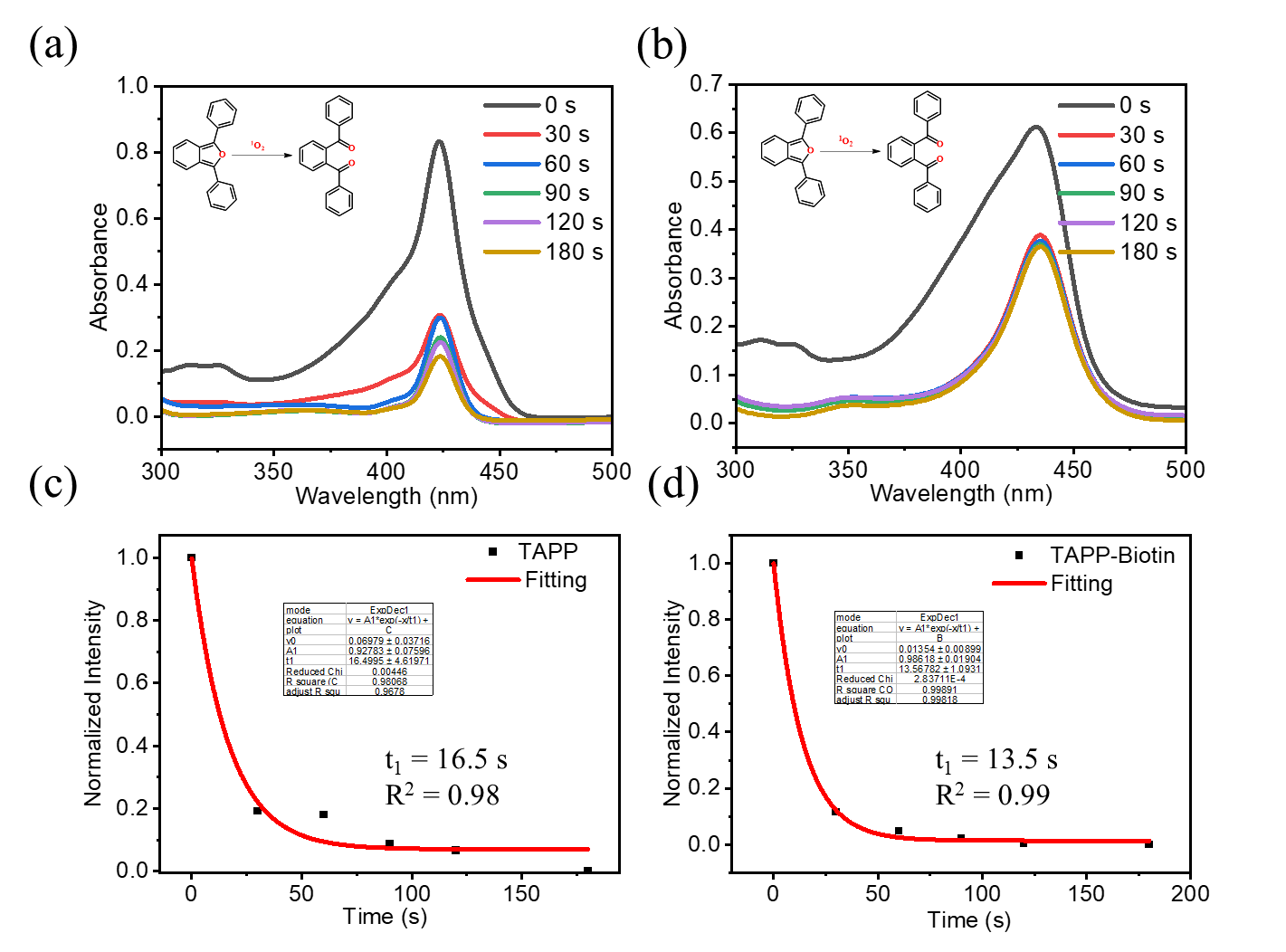
**

**Figure S19.** UV-vis spectra of DPBF (5 1,3-Diphenylisobenzofuran, 50 uM) in DMF containing (a) 5uM TAPP or (b) the TAPP-biotin dyad under irradiation for 180 seconds; their single exponential decay fittings of normalized absorbance were also conducted resulted in lifetimes of 16.5 s and 13.5 s for (c) TAPP and (d) the TAPP-biotin dyad, respectively.

**
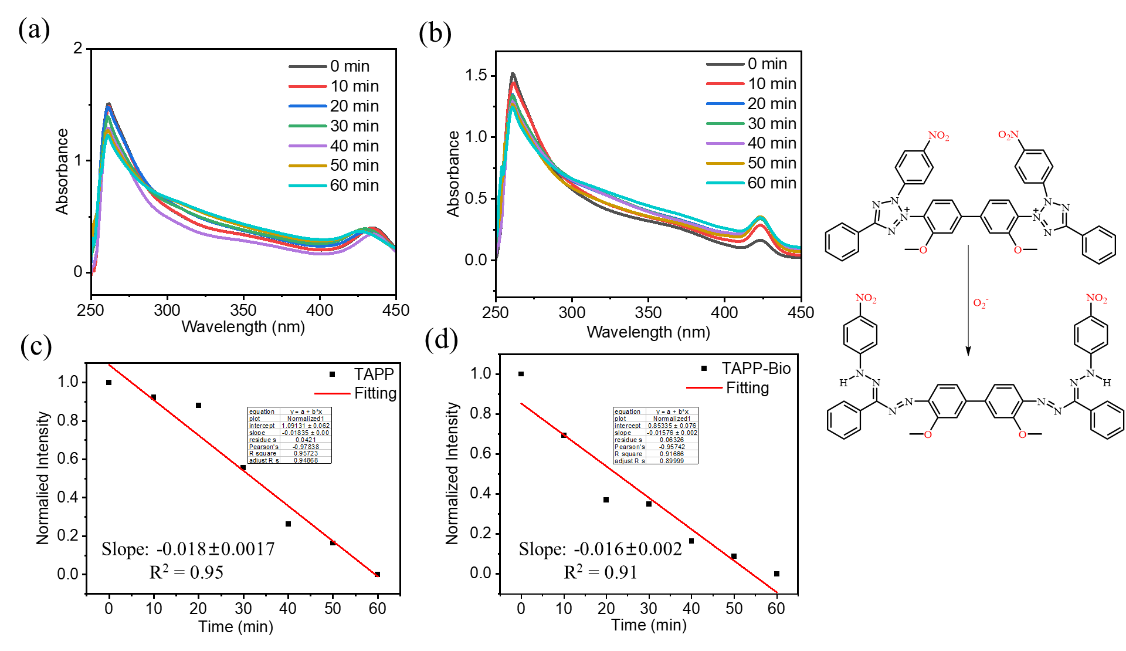
**

**Figure S20.** UV-vis spectra of NBT (Nitrotetrazolium blue, 50 uM) in DMF containing (a) 5uM TAPP and (b) the TAPP-biotin dyad under irradiation for 90 min; their linear decay fittings of normalized absorbance were also conducted resulted in similar values of slope for (c) TAPP and (d) the TAPP-biotin dyad. The reaction between NBT and O_2_^-^ radical was shown here.


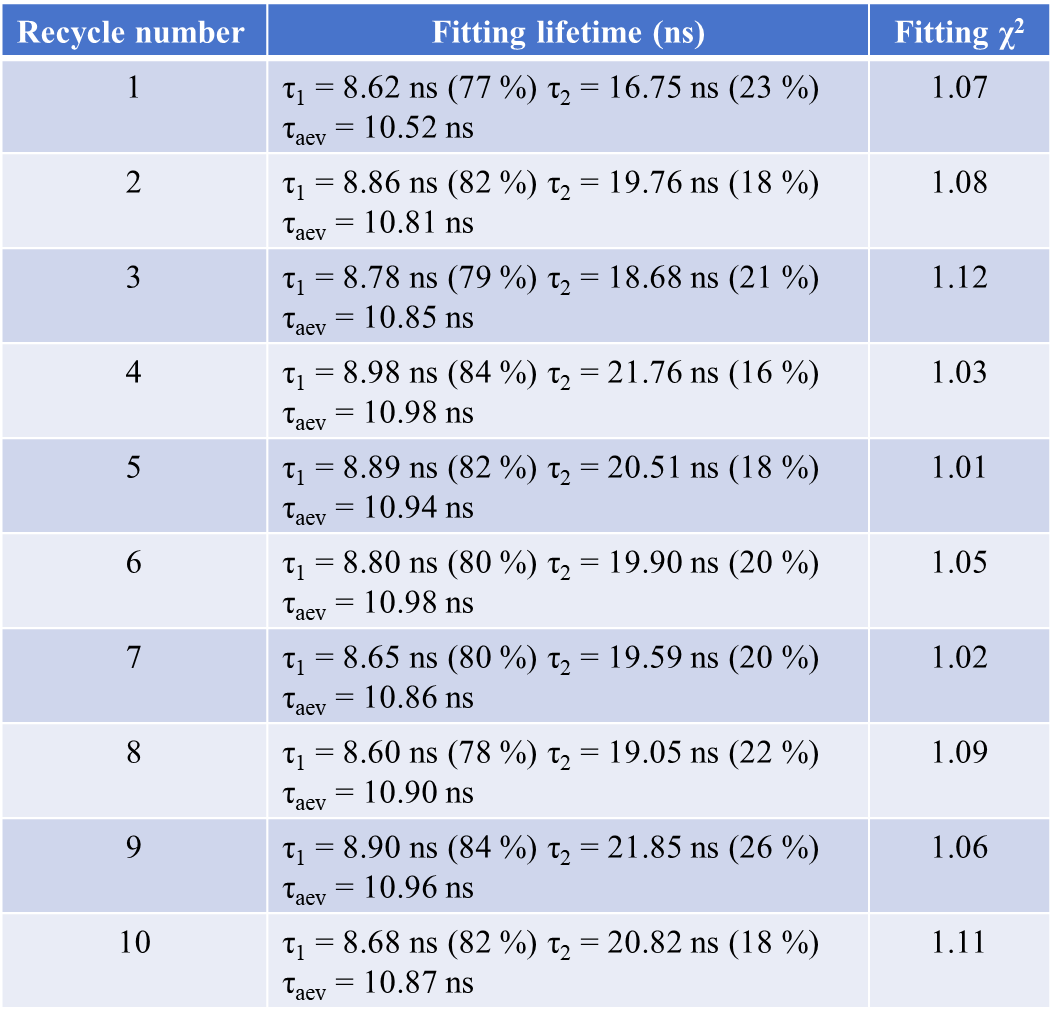


**Table S3.** Summarized fitting results of 10-cycle fluorescence lifetimes.


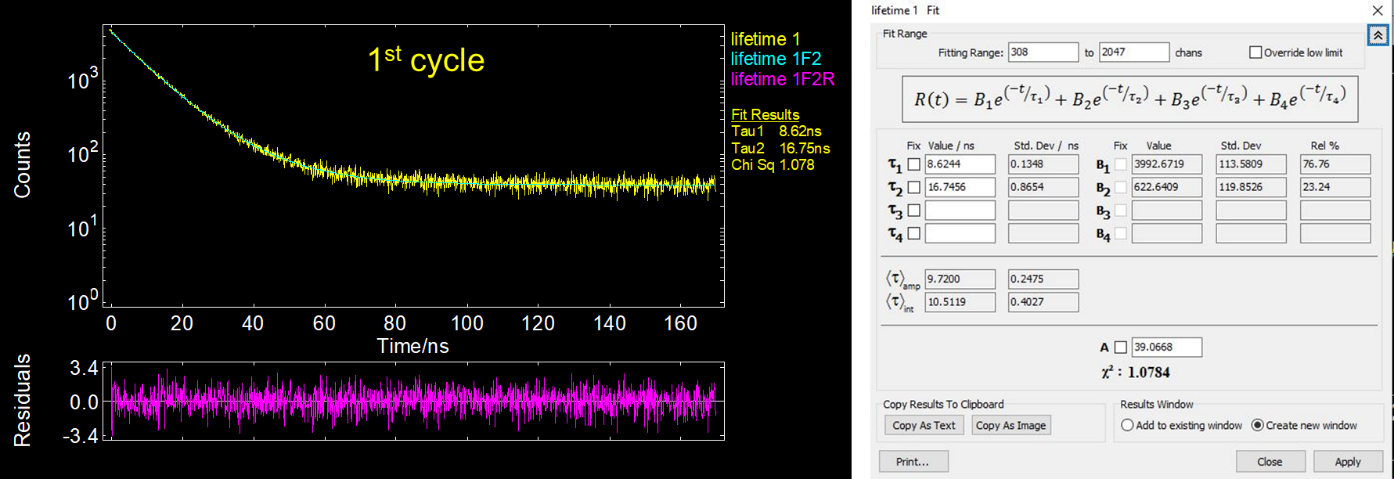


**Figure S21.** Fitting curves and values of fluorescence lifetimes of TBSMB after one test cycles.


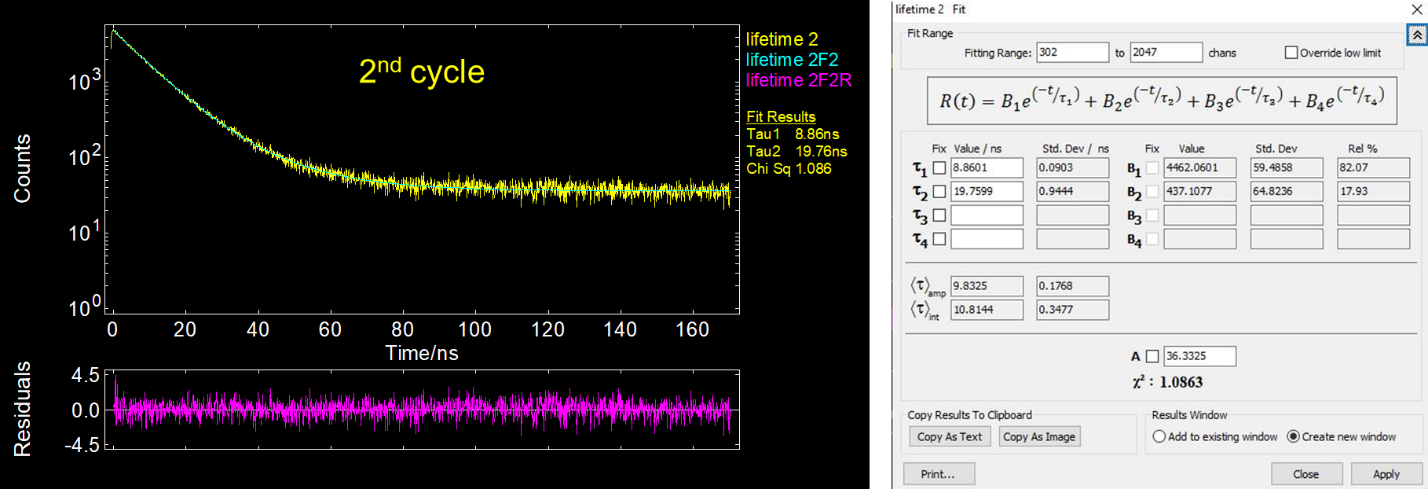


**Figure S22.** Fitting curves and values of fluorescence lifetimes of TBSMB after two cycles.


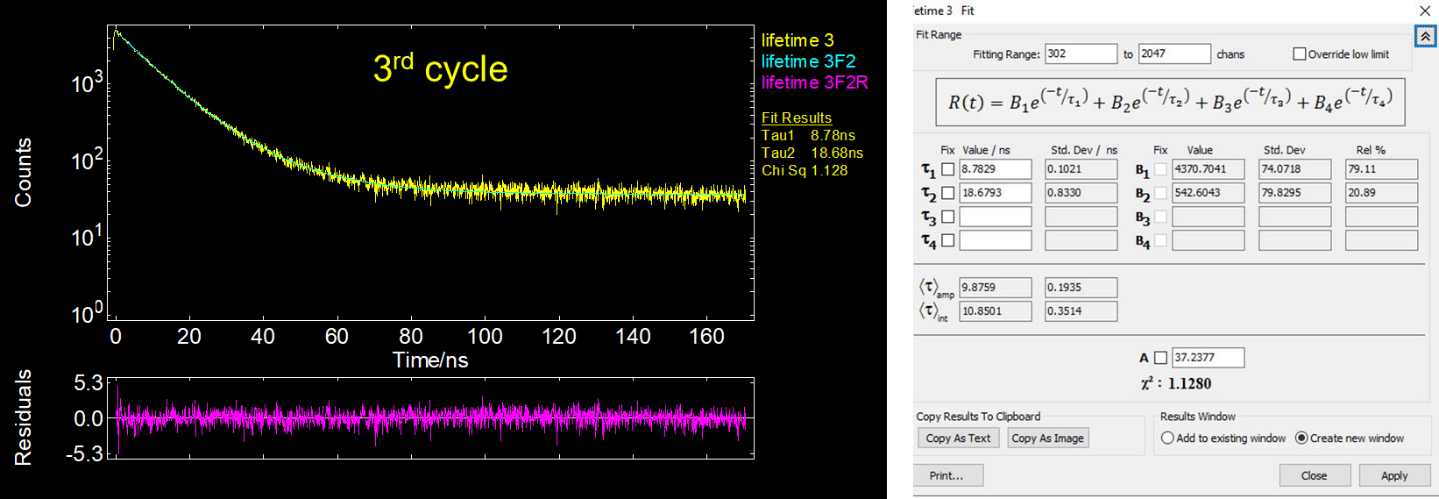


**Figure S23.** Fitting curves and values of fluorescence lifetimes of TBSMB after three cycles.


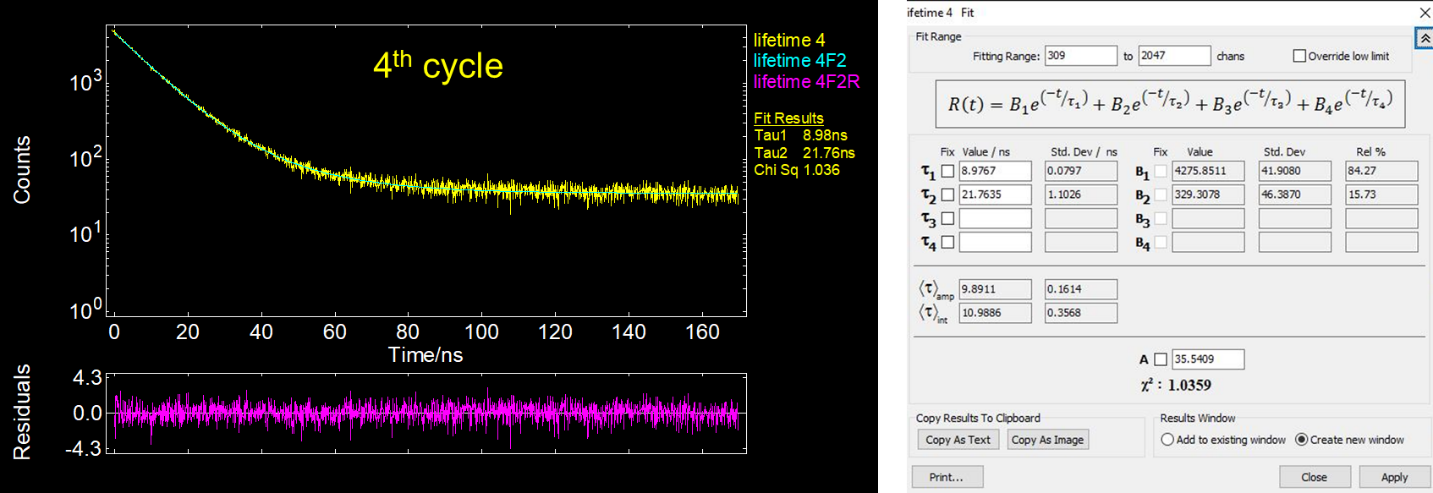


**Figure S24.** Fitting curves and values of fluorescence lifetimes of TBSMB after four cycles.


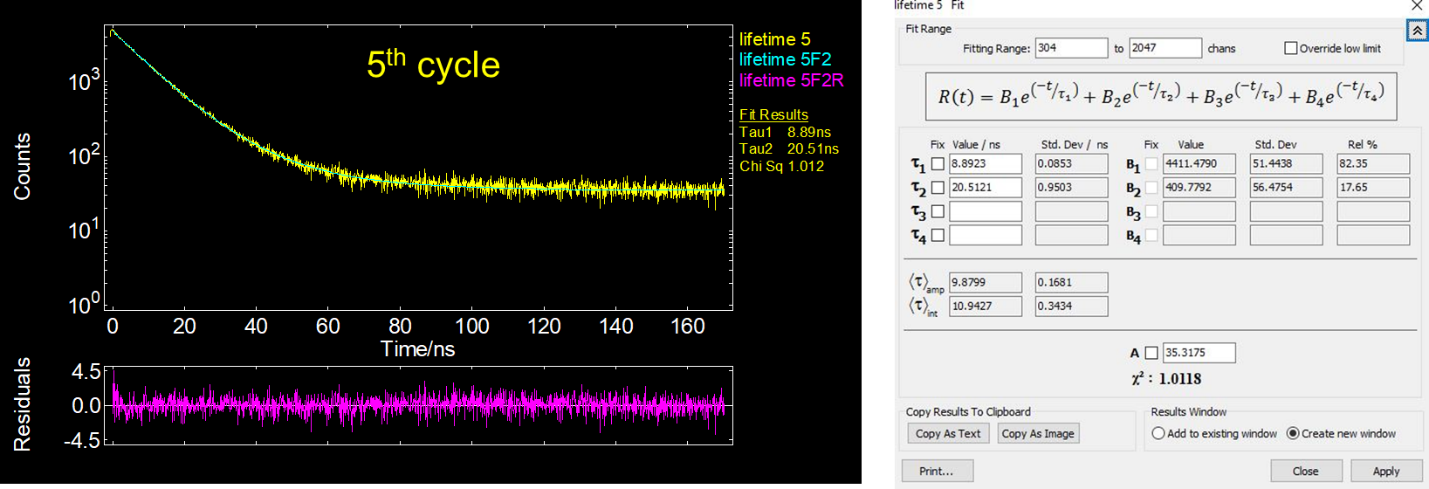


**Figure S25.** Fitting curves and values of fluorescence lifetimes of TBSMB after five cycles.


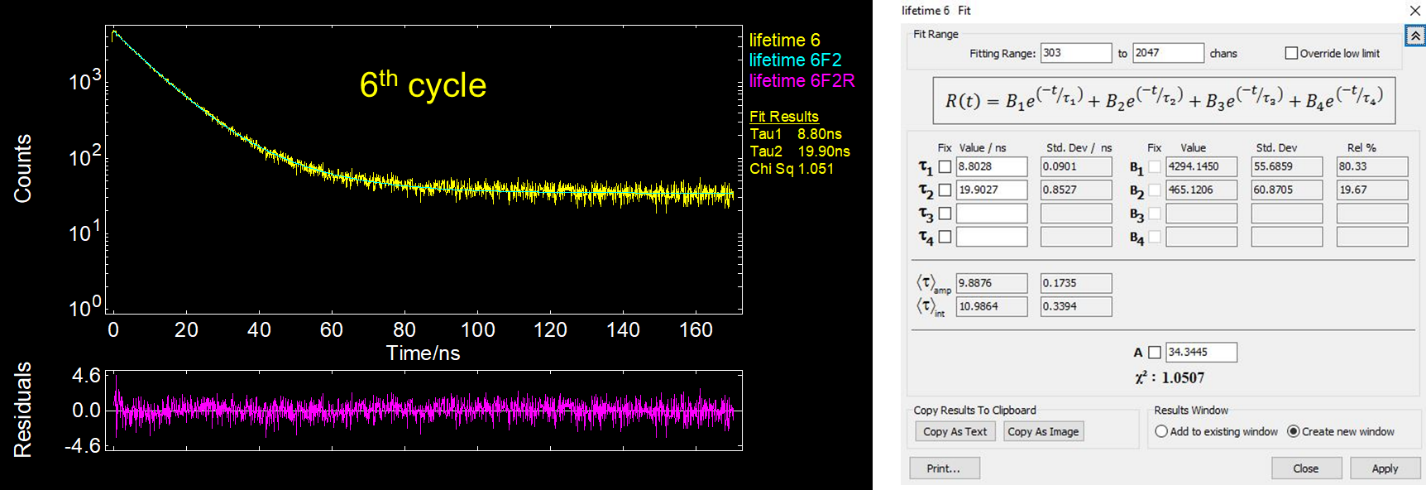


**Figure S26.** Fitting curves and values of fluorescence lifetimes of TBSMB after six cycles.


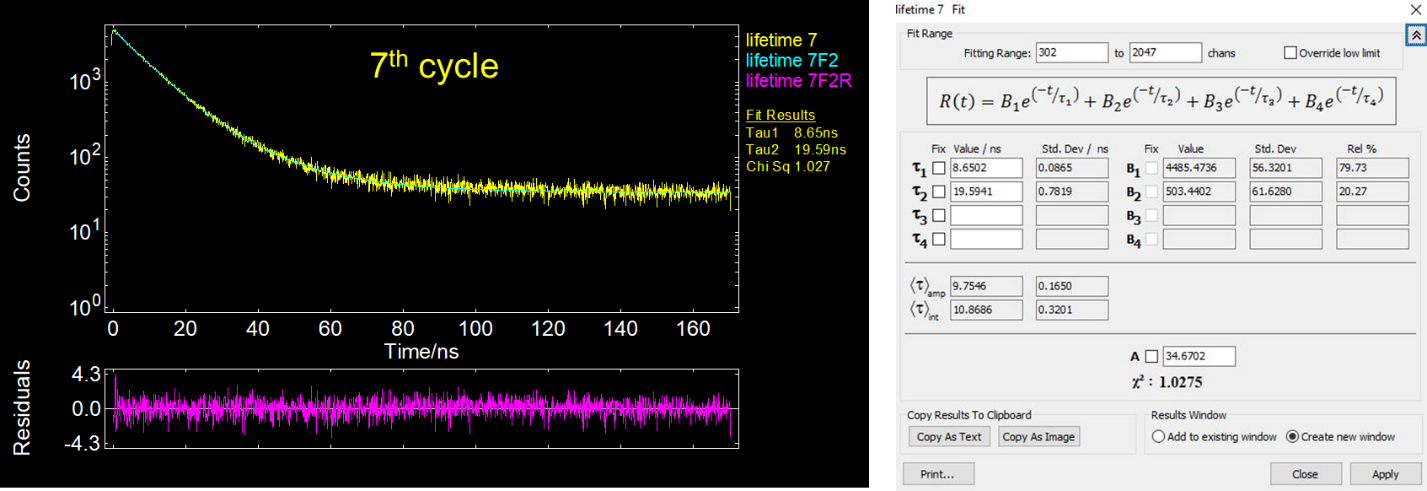


**Figure S27.** Fitting curves and values of fluorescence lifetimes of TBSMB after seven cycles.


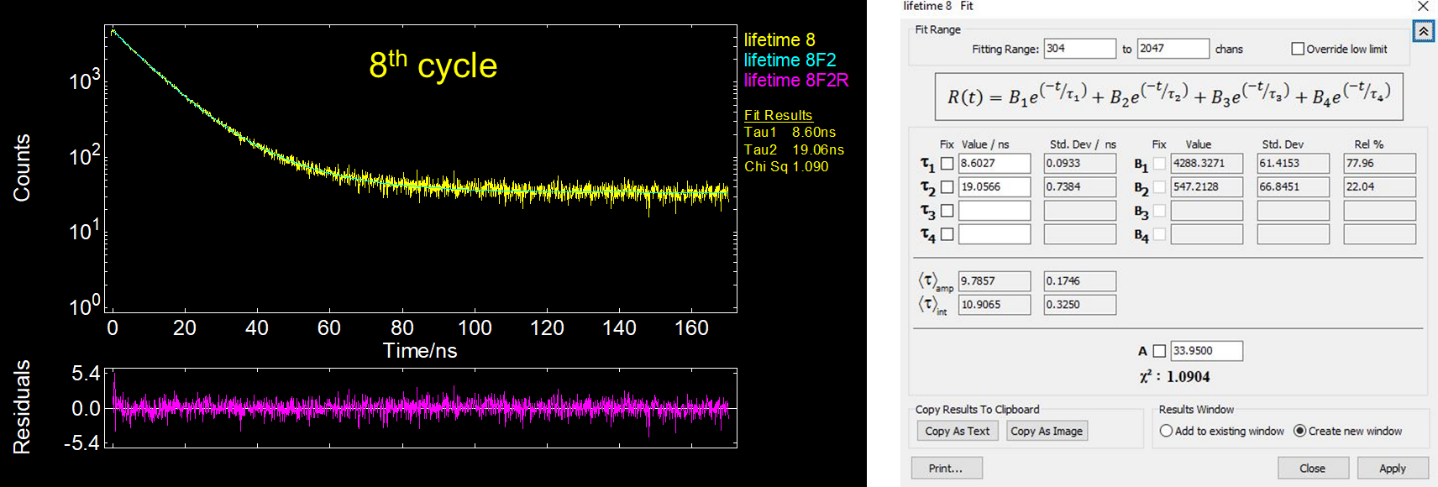


**Figure S28.** Fitting curves and values of fluorescence lifetimes of TBSMB after eight cycles.


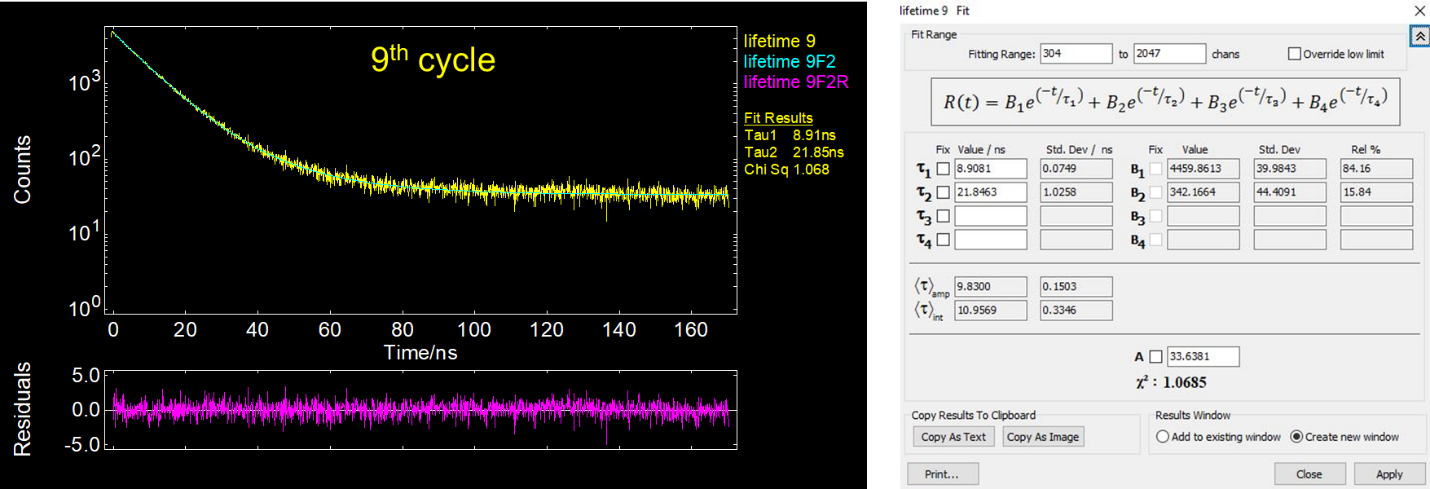


**Figure S29.** Fitting curves and values of fluorescence lifetimes of TBSMB after nine cycles.


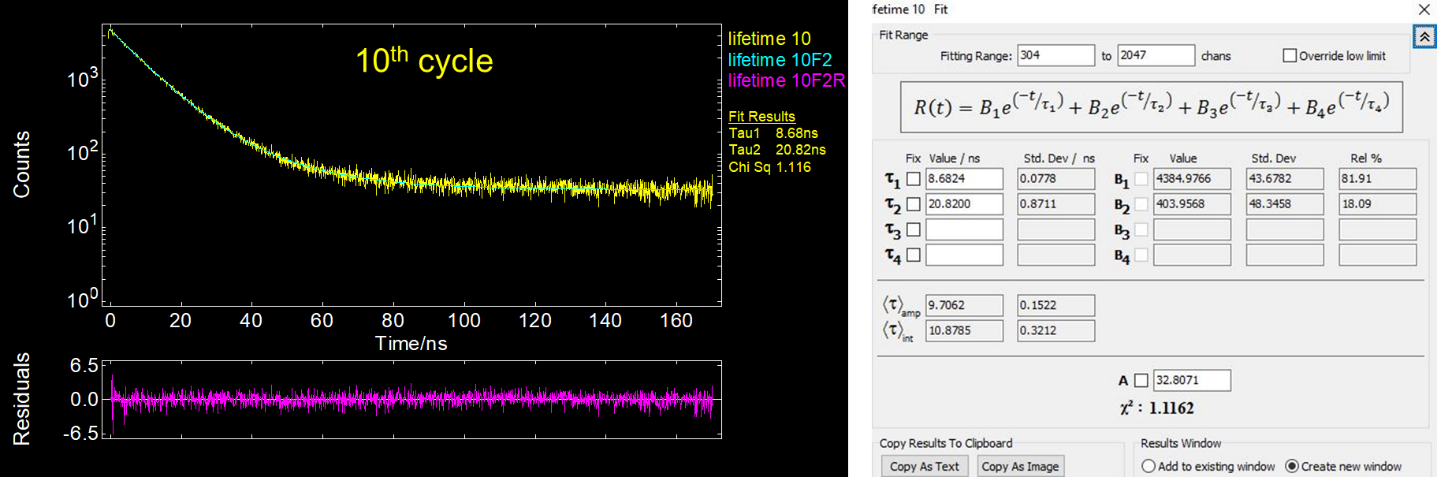


**Figure S30.** Fitting curves and values of fluorescence lifetimes of TBSMB after ten cycles.

**
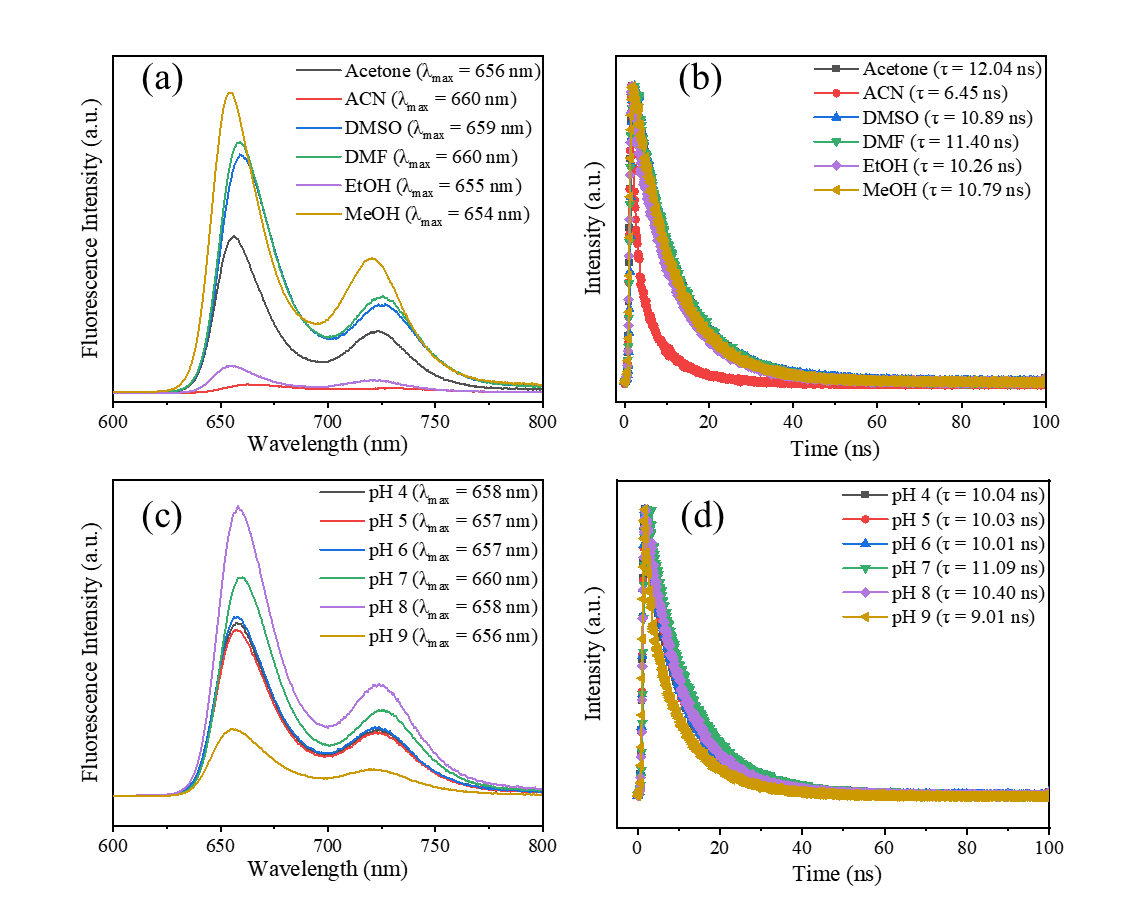
**

**Figure S31.** (a) Fluorescence emission spectra of TBSMB (10 uL) in various solvents and (b) the corresponding measured lifetimes; (c) Fluorescence emission spectra of TBSMB (10 uL) in various PBS buffer-DMSO with different pH 4-9 and (d) the corresponding lifetimes.

**Figure S32-S70 are the GC-MS data for both photocatalytic oxidative coupling of benzylamine and its derivatives (24 substrates 1-24), and oxidation of thioanisole and its derivatives (15 substrates 25-39).**


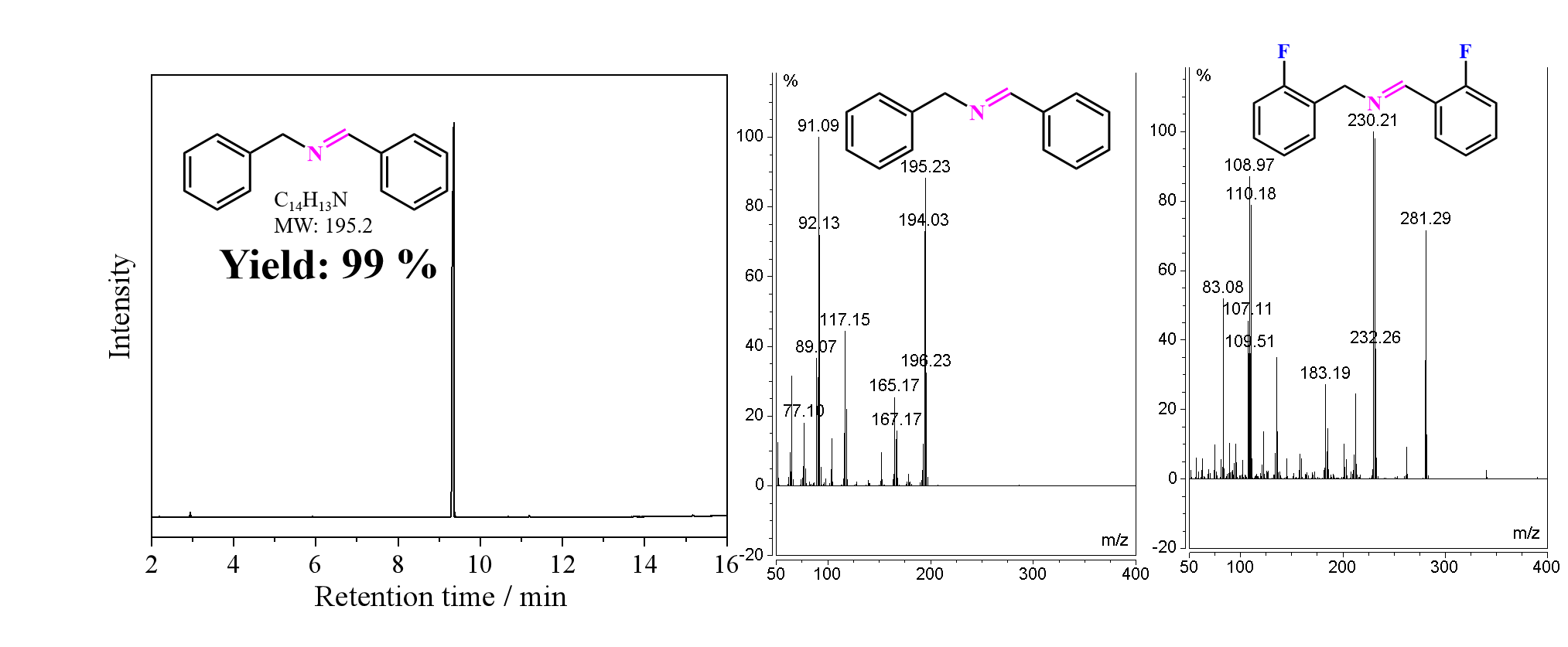


**Figure S32.** GC trace for the imine **1** formation and the corresponding MS spectra.


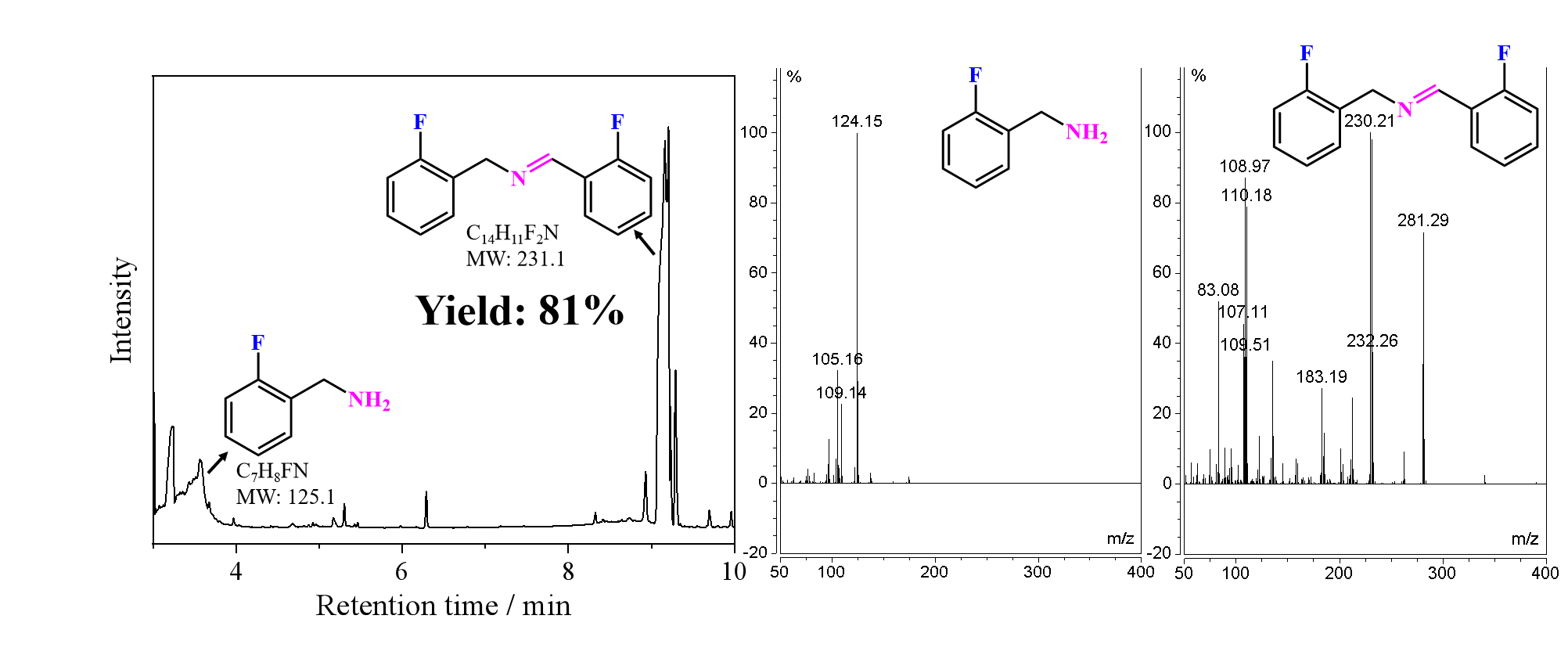


**Figure S33.** GC trace for the imine **2** formation and the corresponding MS spectra.


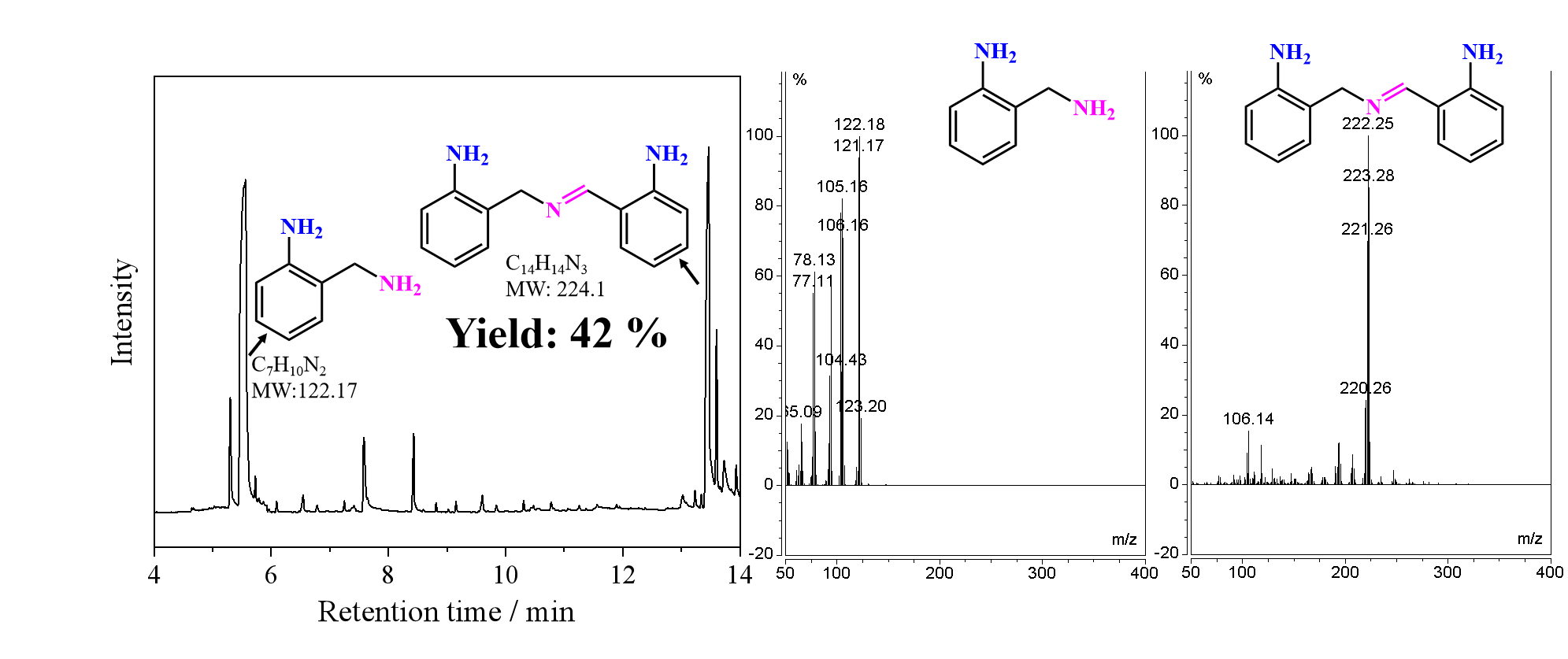


**Figure S34.** GC trace for the imine **3** formation and the corresponding MS spectra.


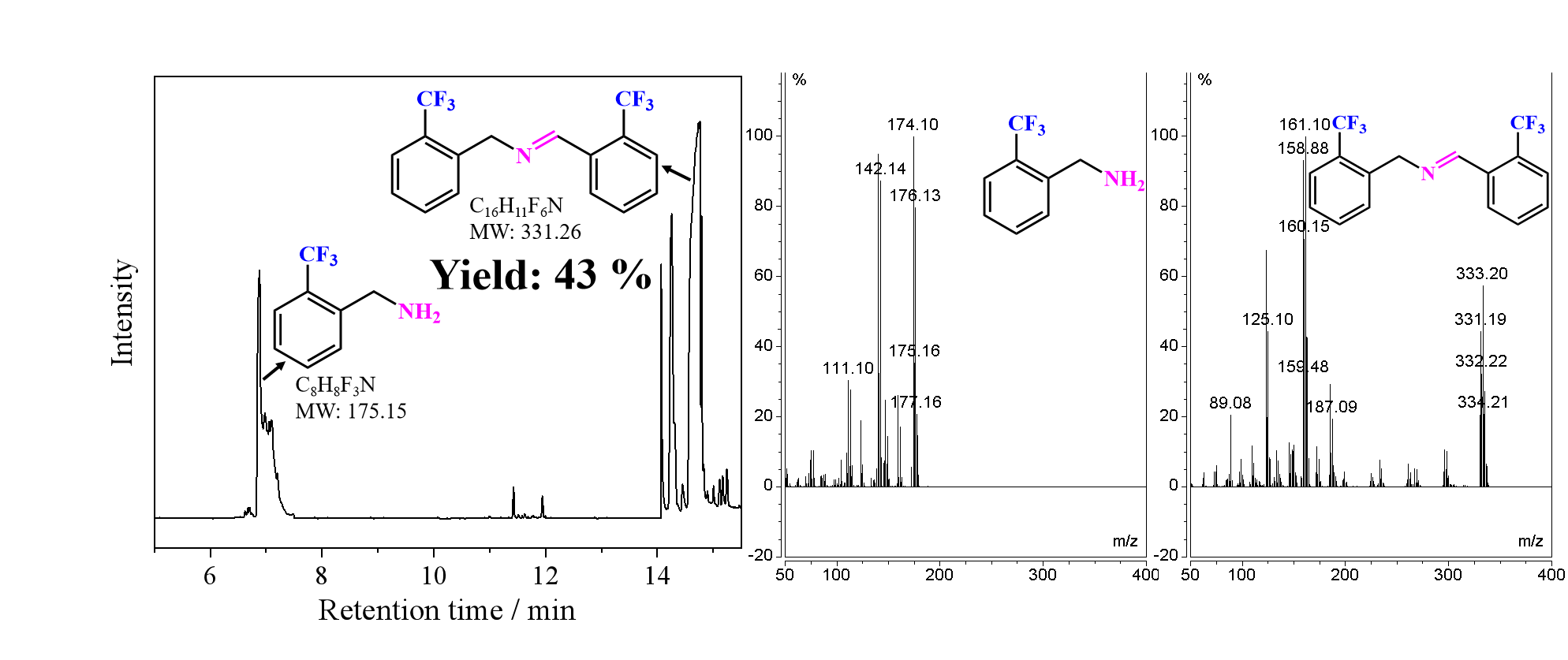


**Figure S35.** GC trace for the imine **4** formation and the corresponding MS spectra.


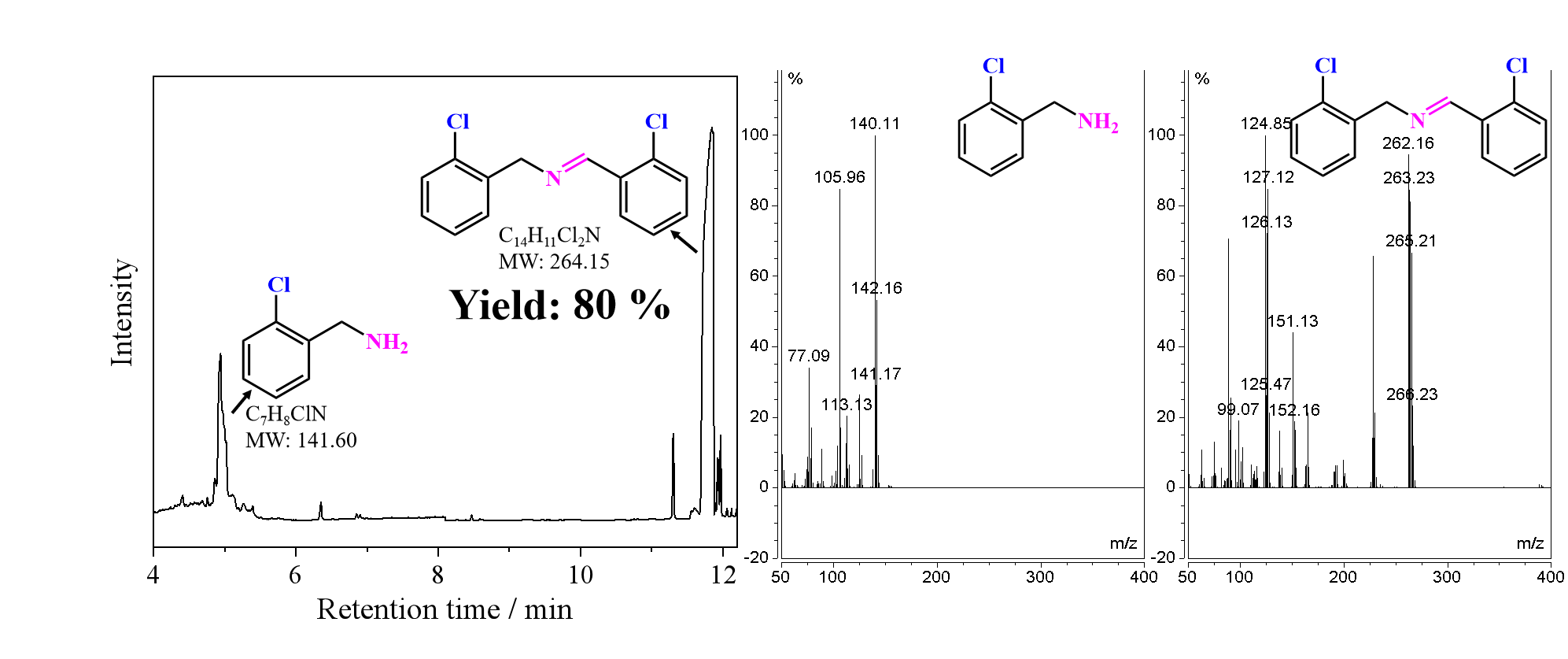


**Figure S36.** GC trace for the imine **5** formation and the corresponding MS spectra.


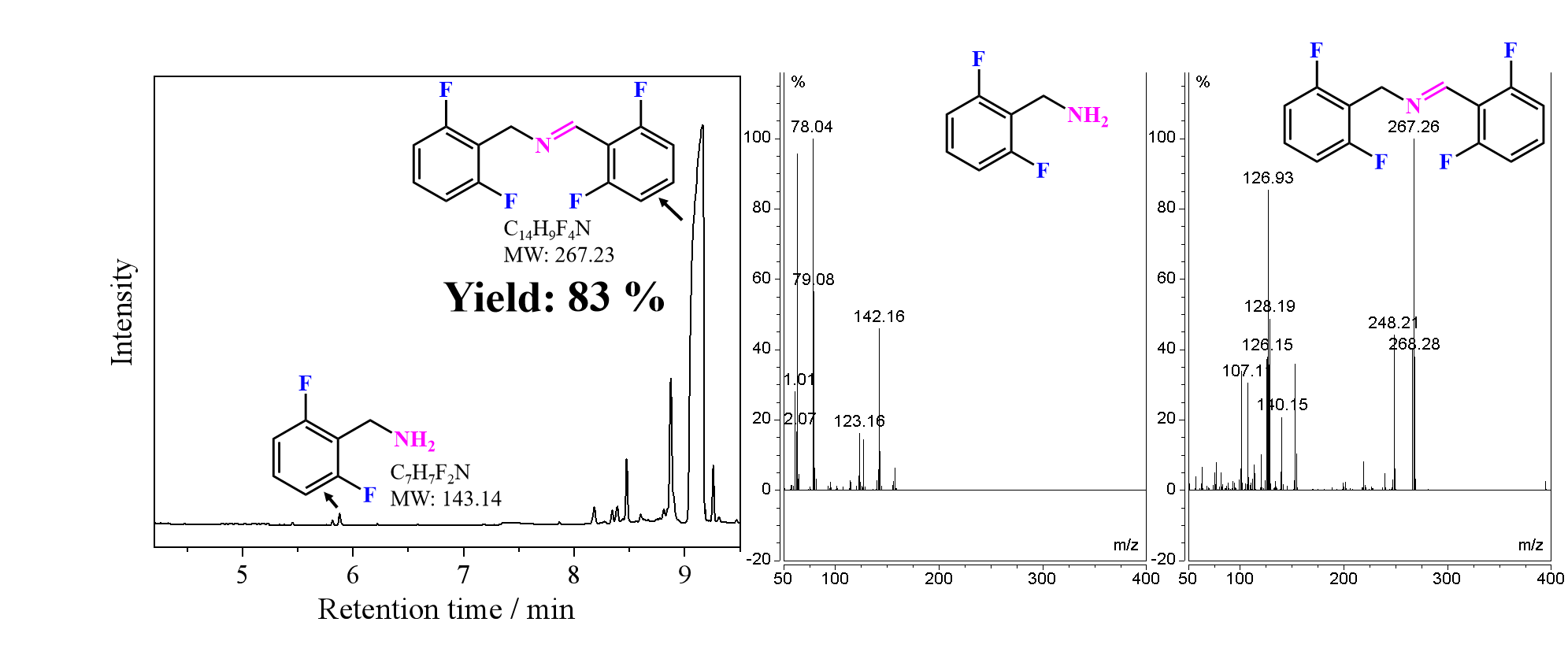


**Figure S37.** GC trace for the imine **6** formation and the corresponding MS spectra.


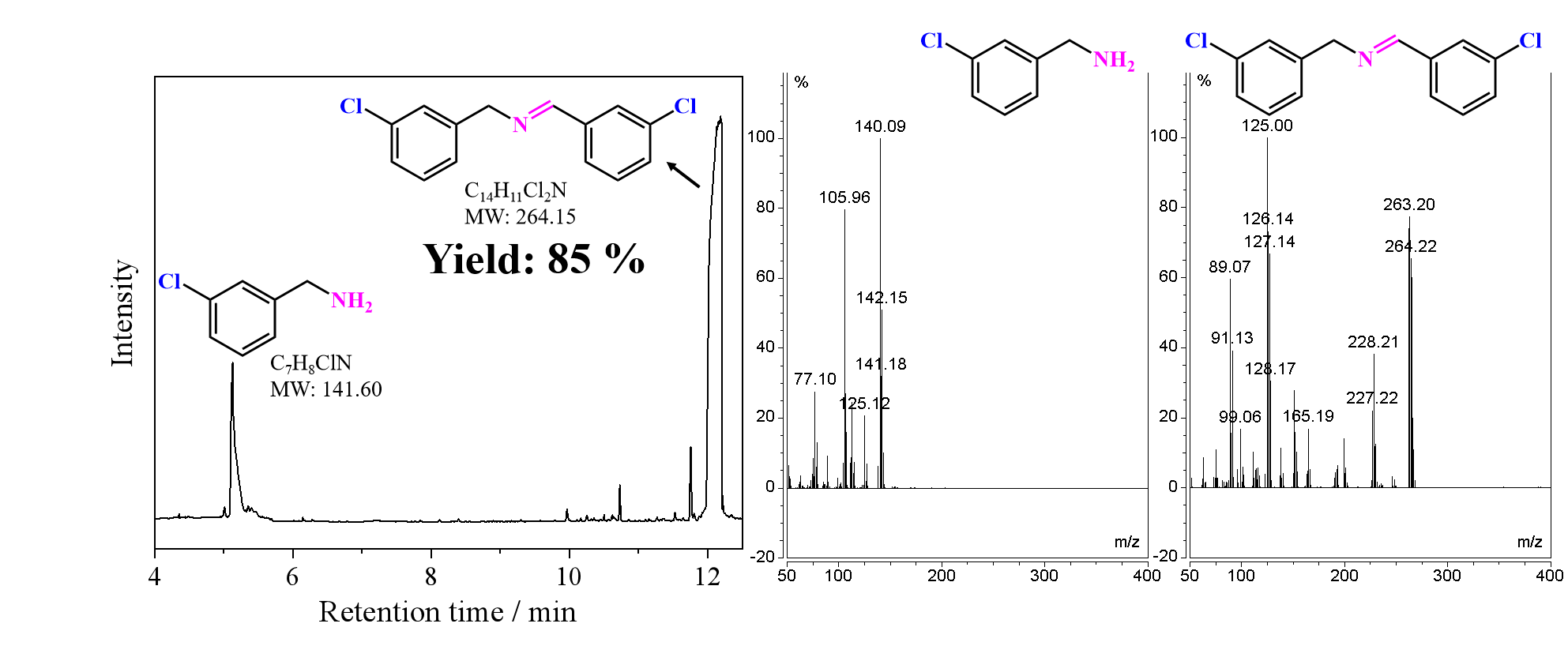


**Figure S38.** GC trace for the imine **7** formation and the corresponding MS spectra.


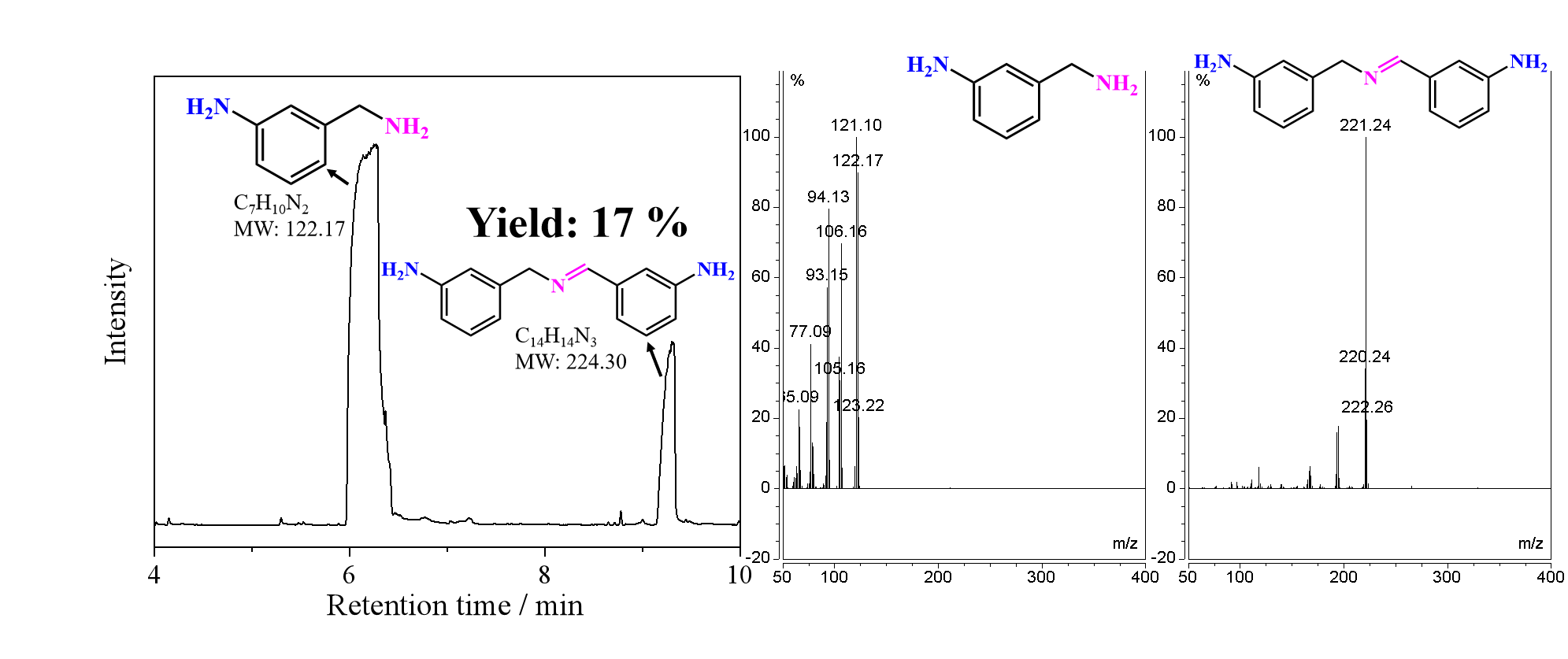


**Figure S39.** GC trace for the imine **8** formation and the corresponding MS spectra.


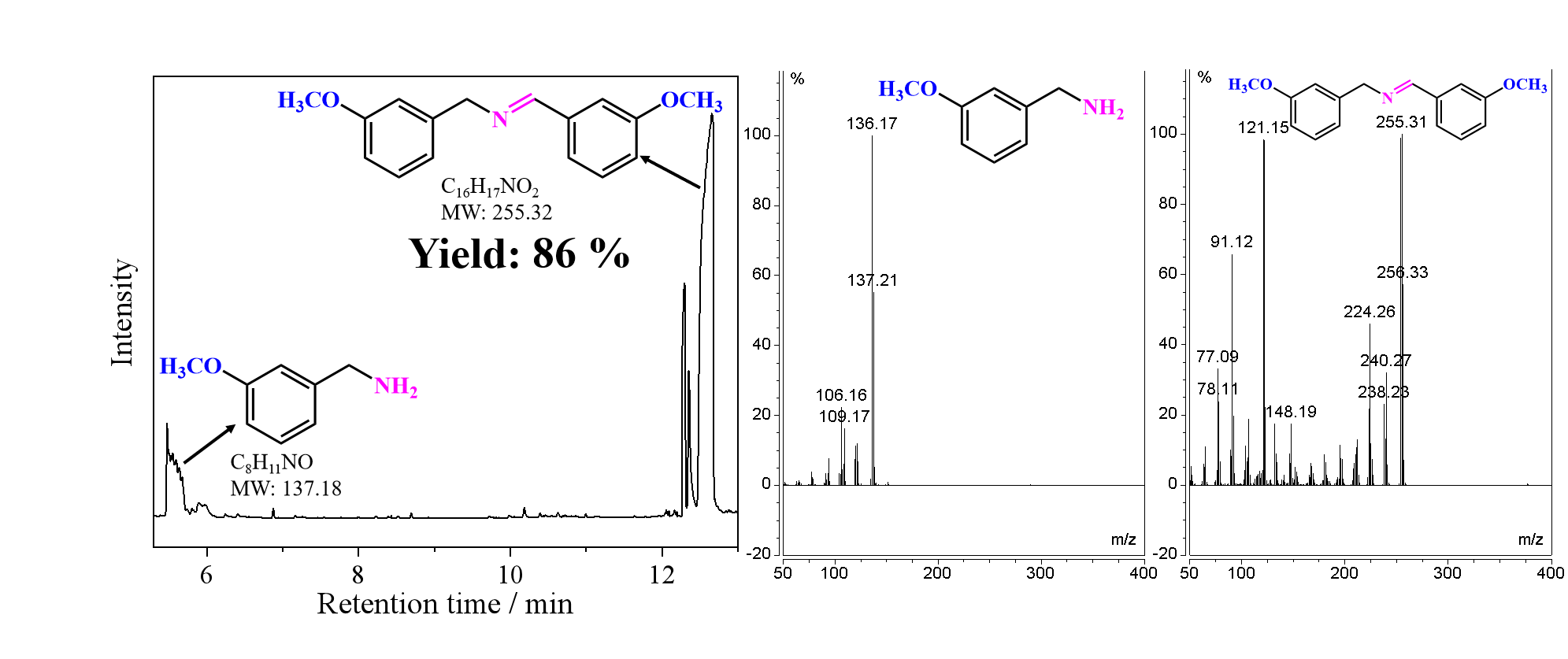


**Figure S40.** GC trace for the imine **9** formation and the corresponding MS spectra.


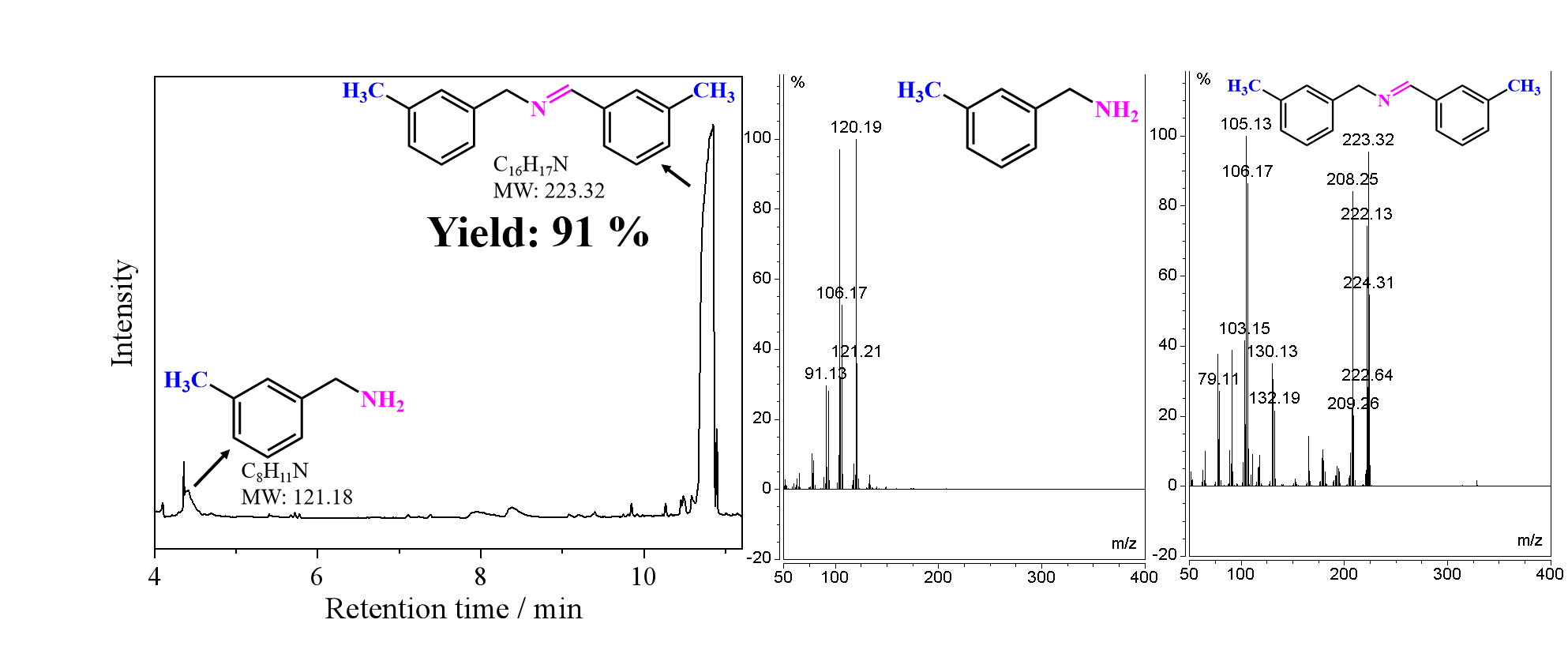


**Figure S41.** GC trace for the imine **10** formation and the corresponding MS spectra.


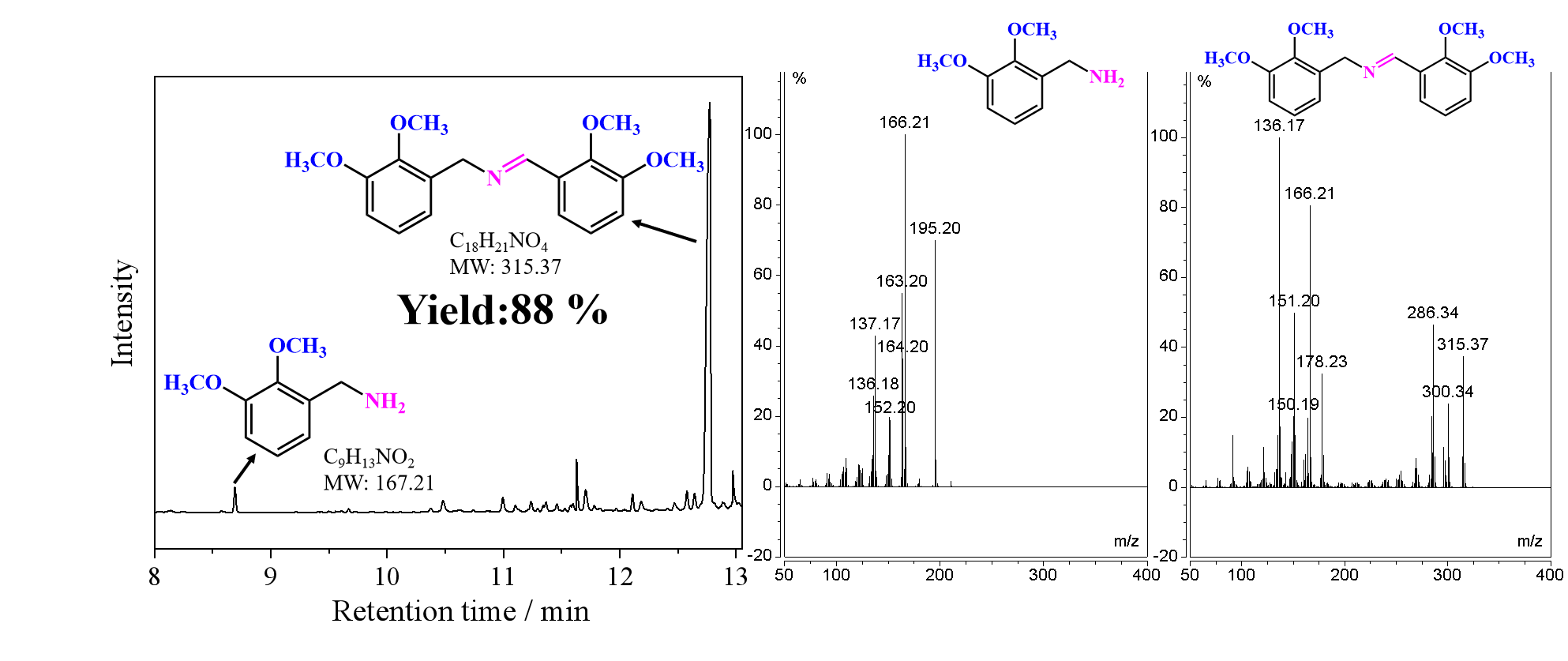


**Figure S42.** GC trace for the imine **11** formation and the corresponding MS spectra.


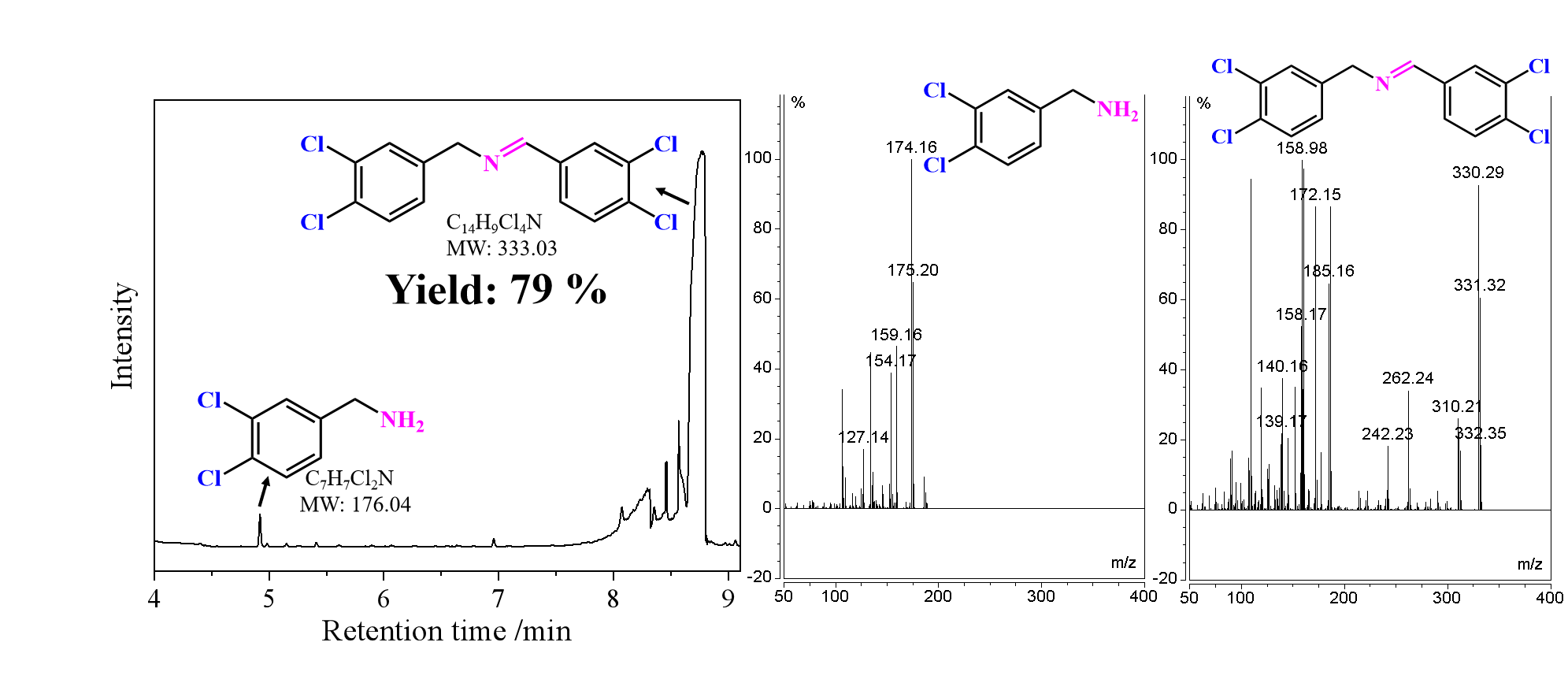


**Figure S43.** GC trace for the imine **12** formation and the corresponding MS spectra.


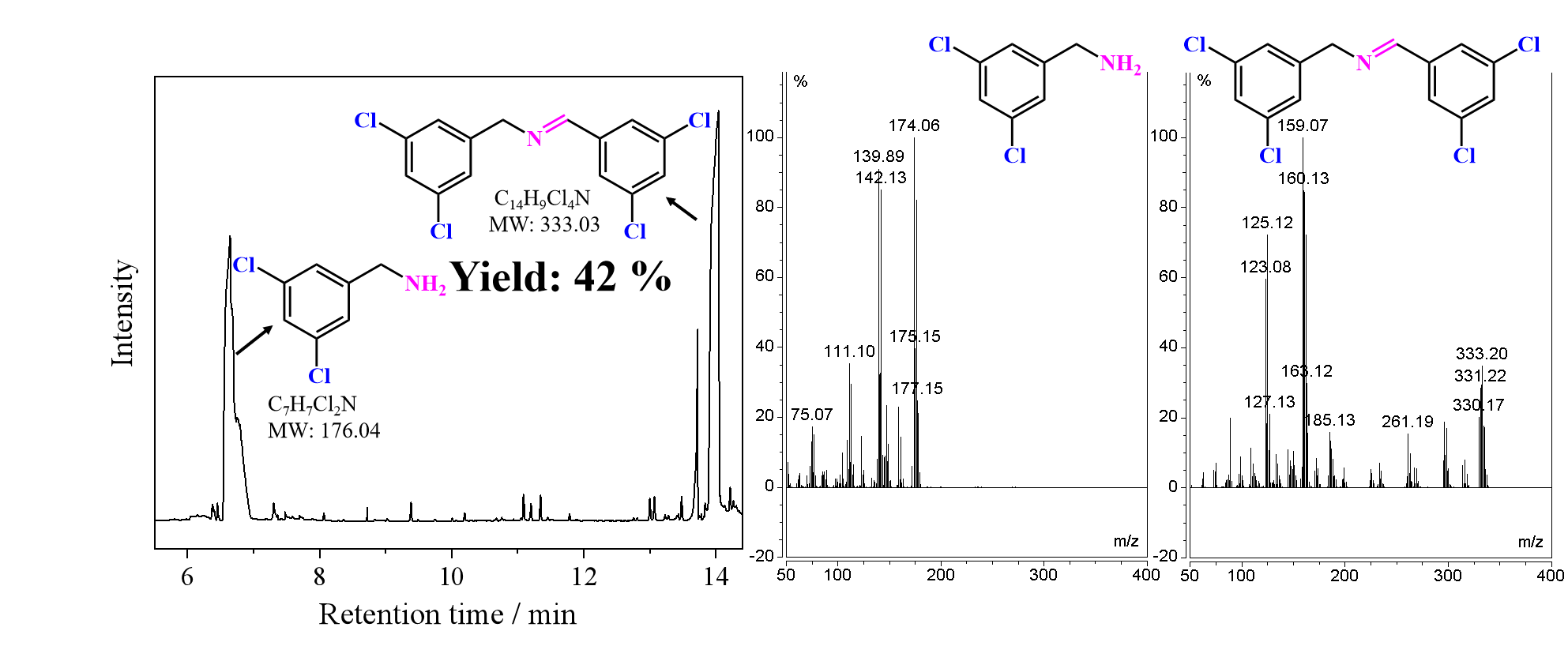


**Figure S44.** GC trace for the imine **13** formation and the corresponding MS spectra.


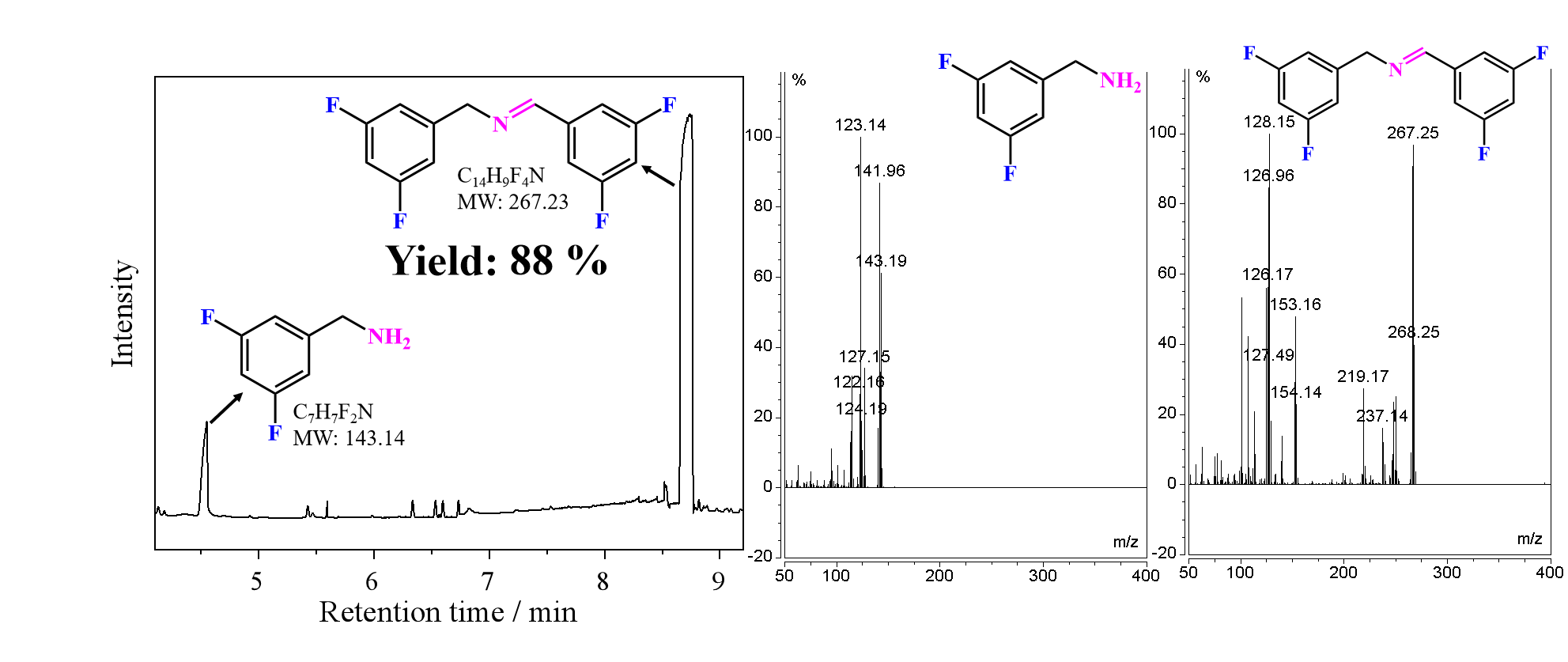


**Figure S45.** GC trace for the imine **14** formation and the corresponding MS spectra.


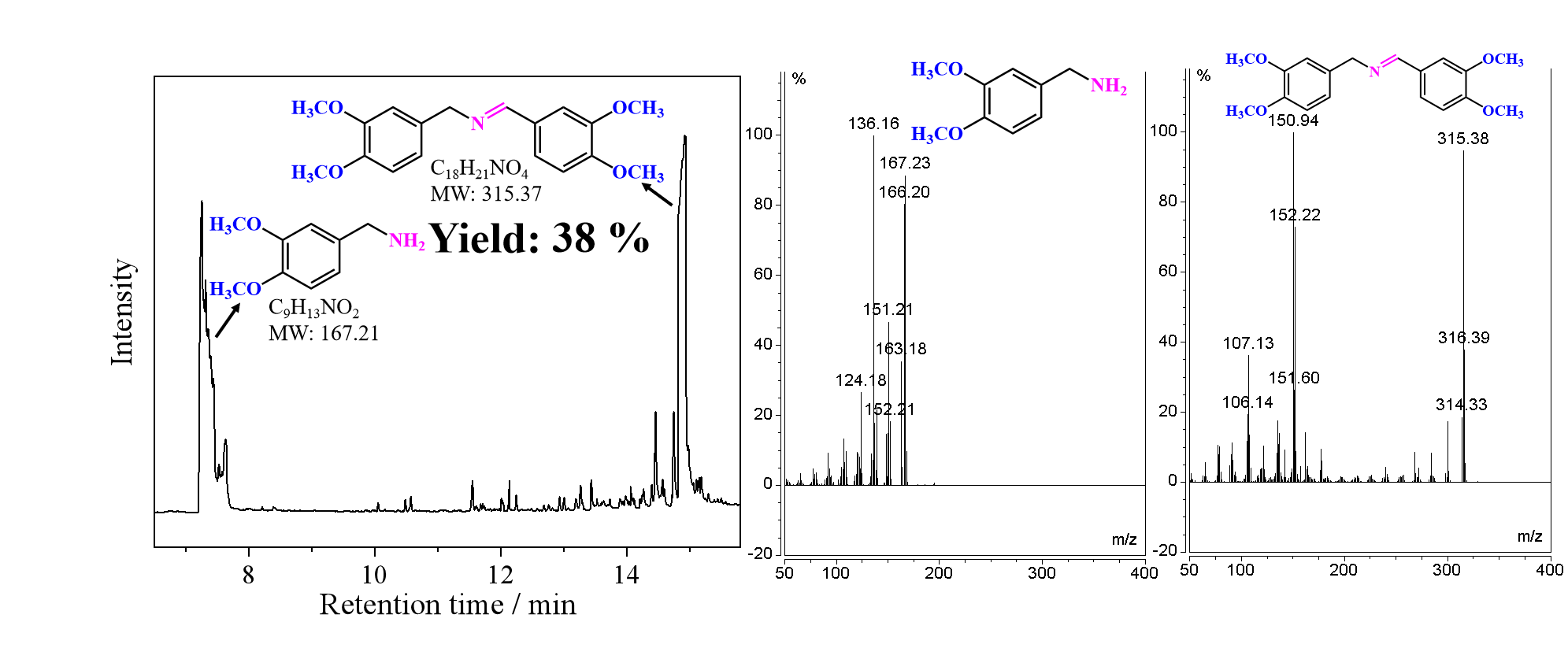


**Figure S46.** GC trace for the imine **15** formation and the corresponding MS spectra.


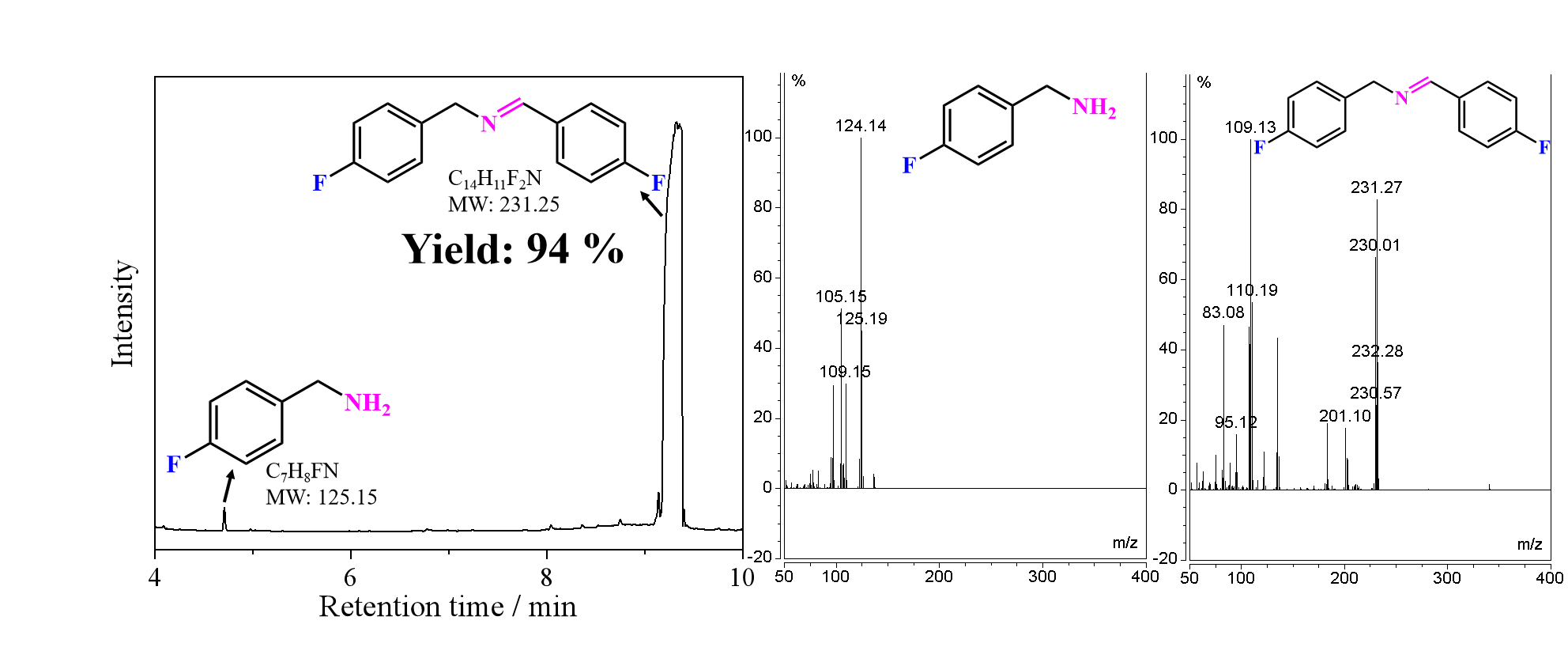


**Figure S47.** GC trace for the imine **16** formation and the corresponding MS spectra.


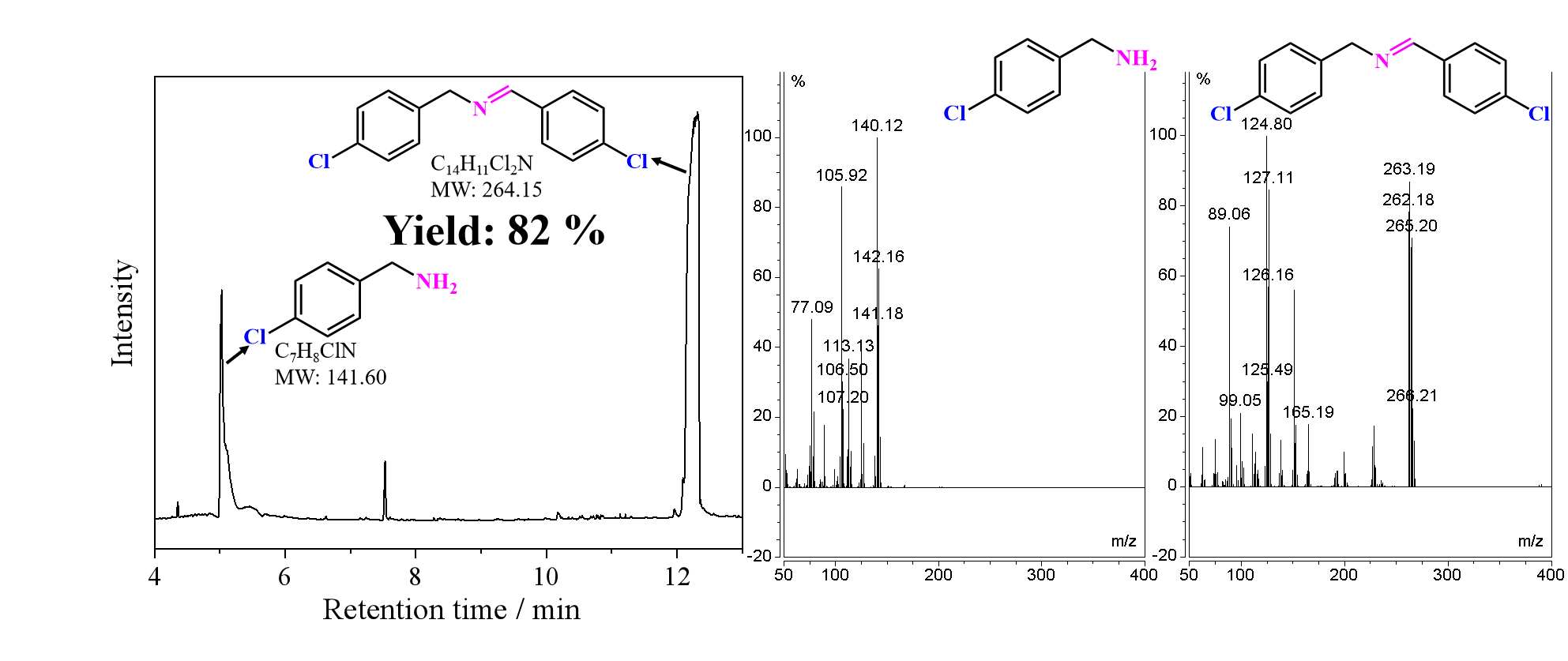


**Figure S48.** GC trace for the imine **17** formation and the corresponding MS spectra.


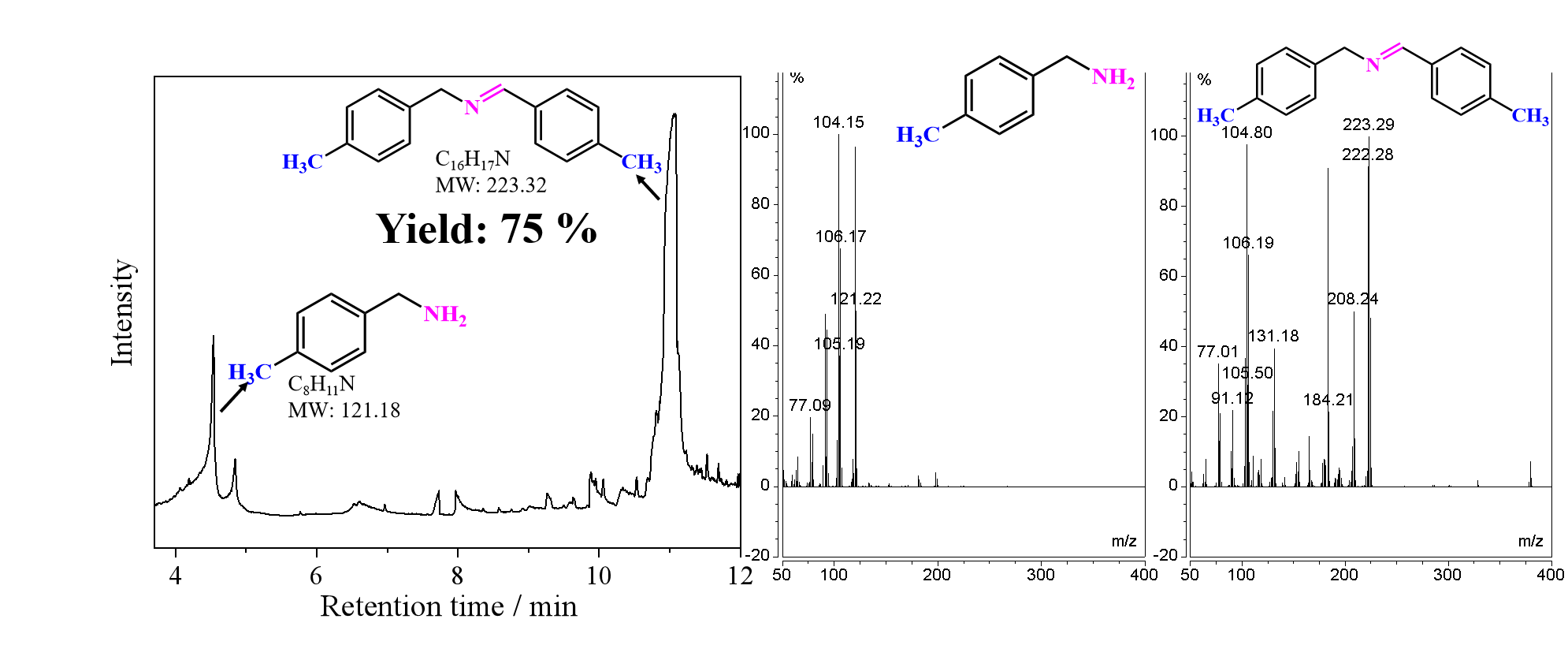


**Figure S49.** GC trace for the imine **18** formation and the corresponding MS spectra.


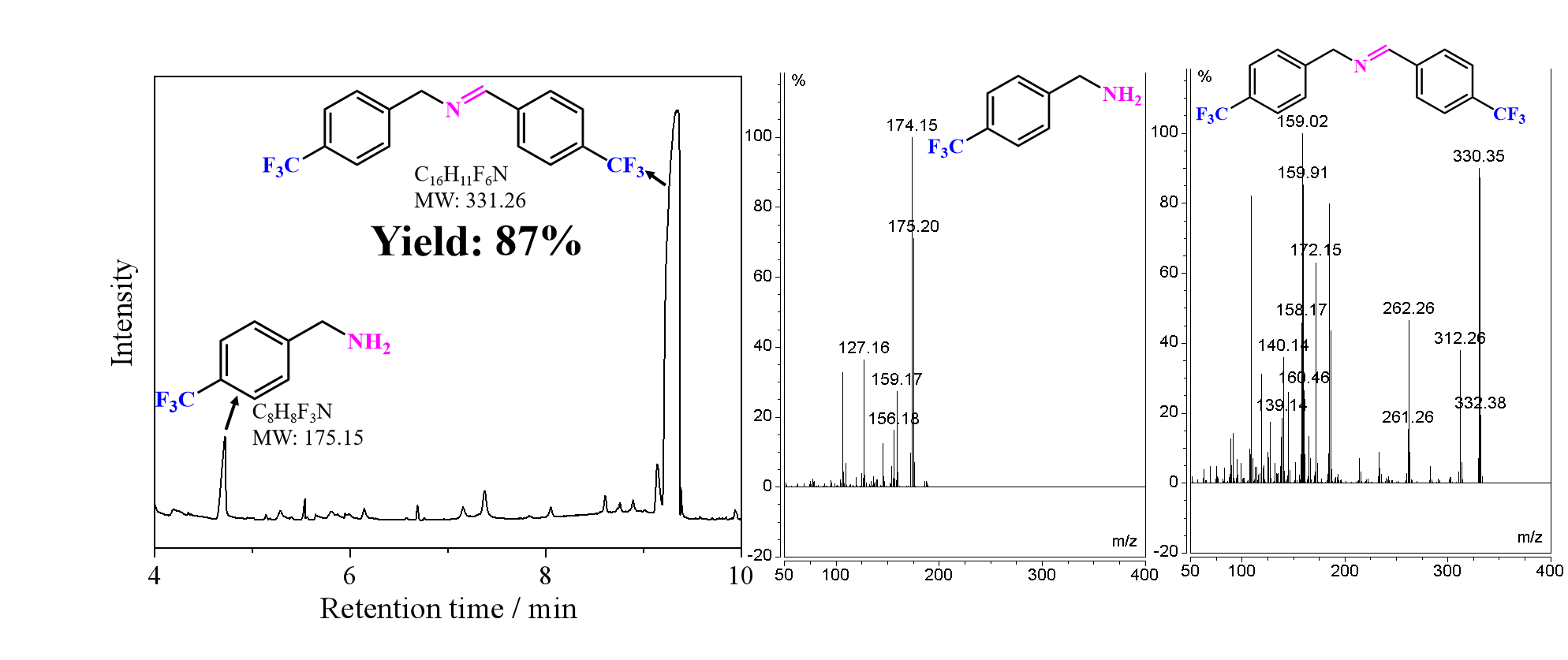


**Figure S50.** GC trace for the imine **19** formation and the corresponding MS spectra.


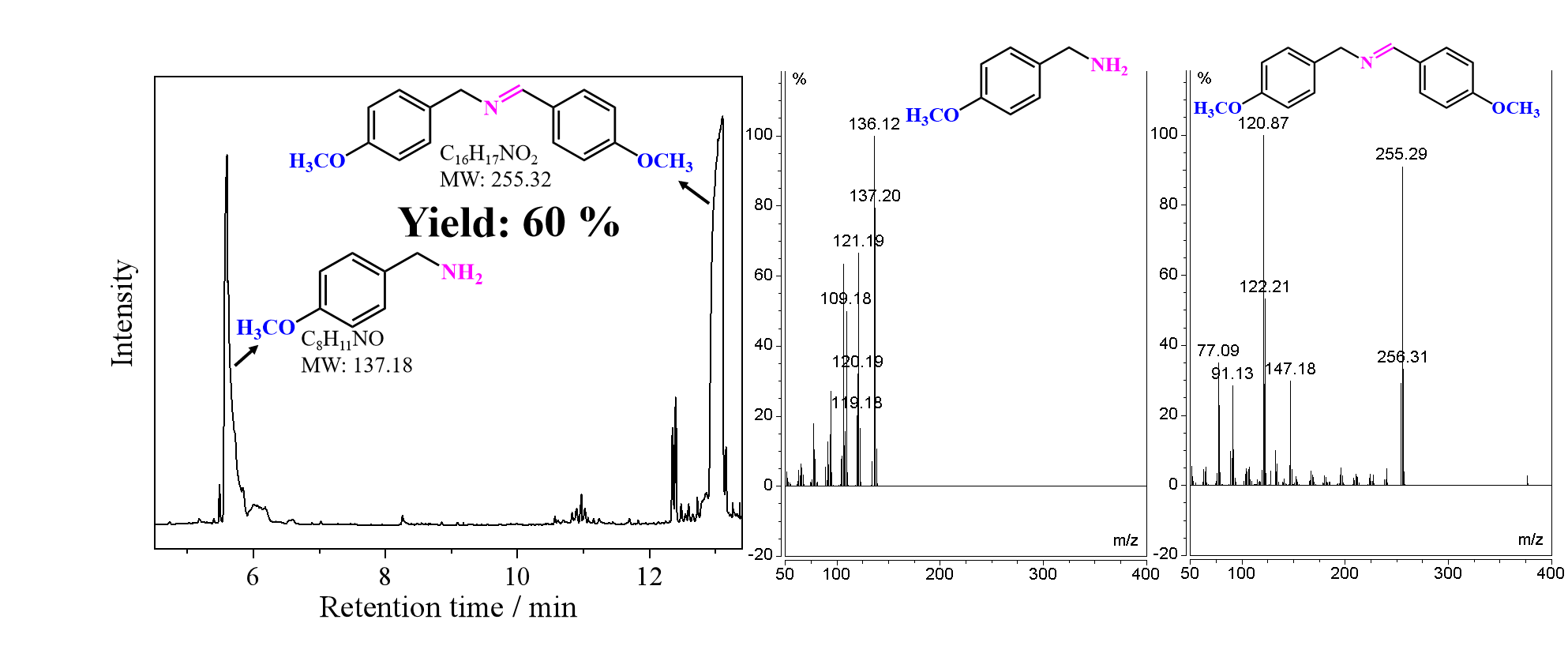


**Figure S51.** GC trace for the imine **20** formation and the corresponding MS spectra.


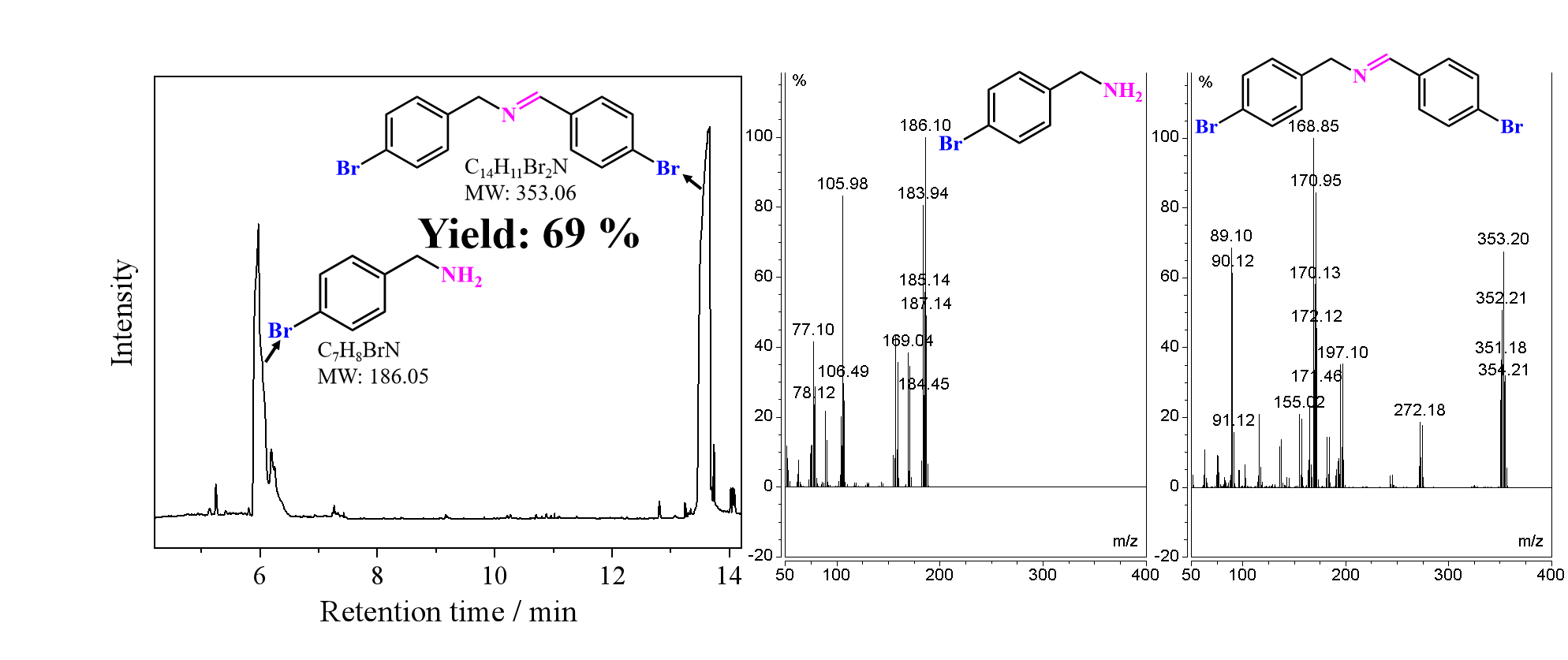


**Figure S52.** GC trace for the imine **21** formation and the corresponding MS spectra.


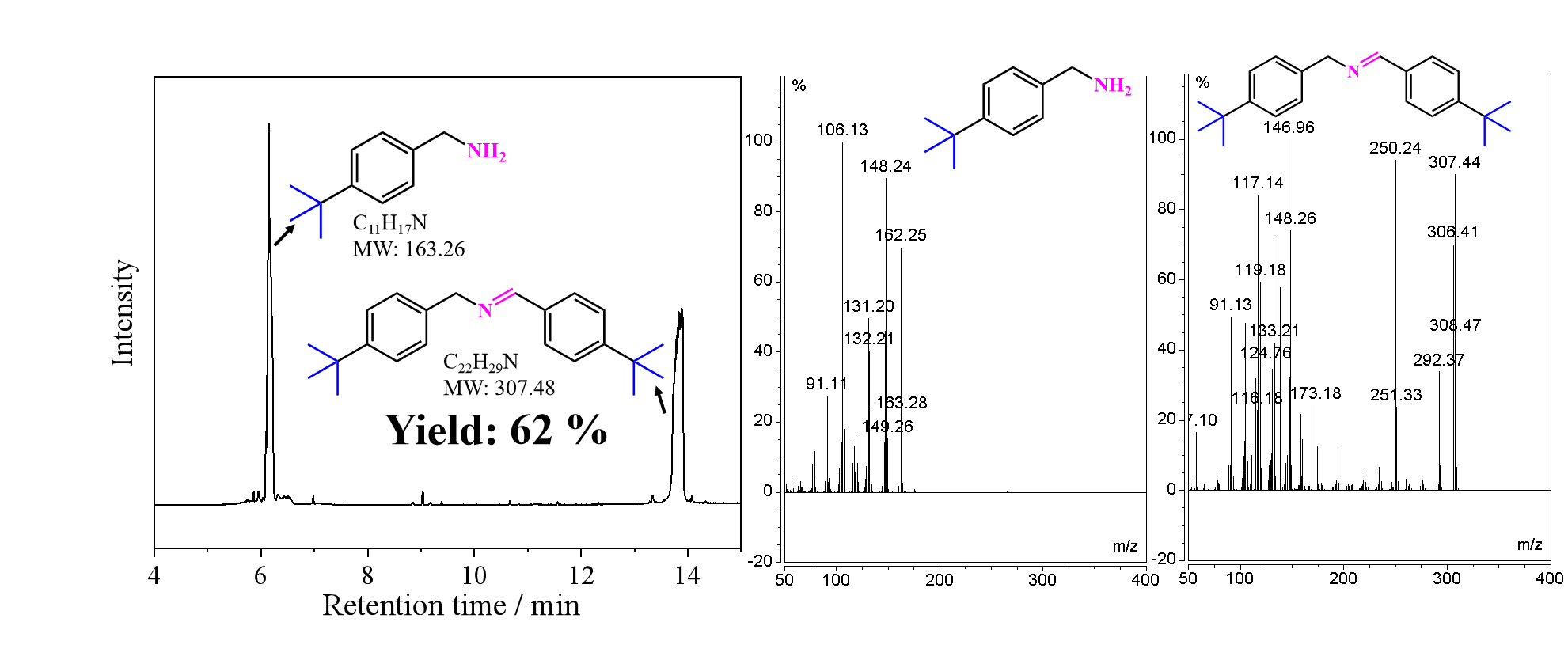


**Figure S53.** GC trace for the imine **22** formation and the corresponding MS spectra.


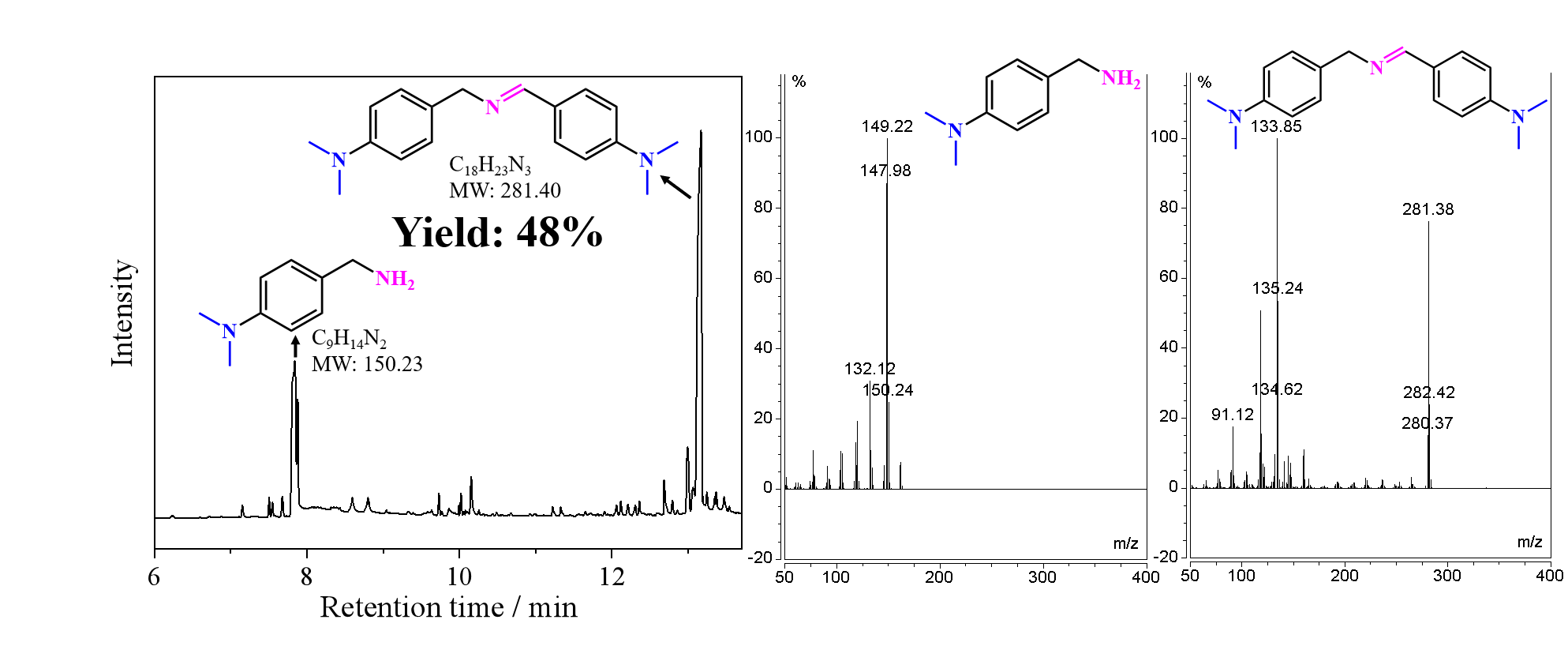


**Figure S54.** GC trace for the imine **23** formation and the corresponding MS spectra.


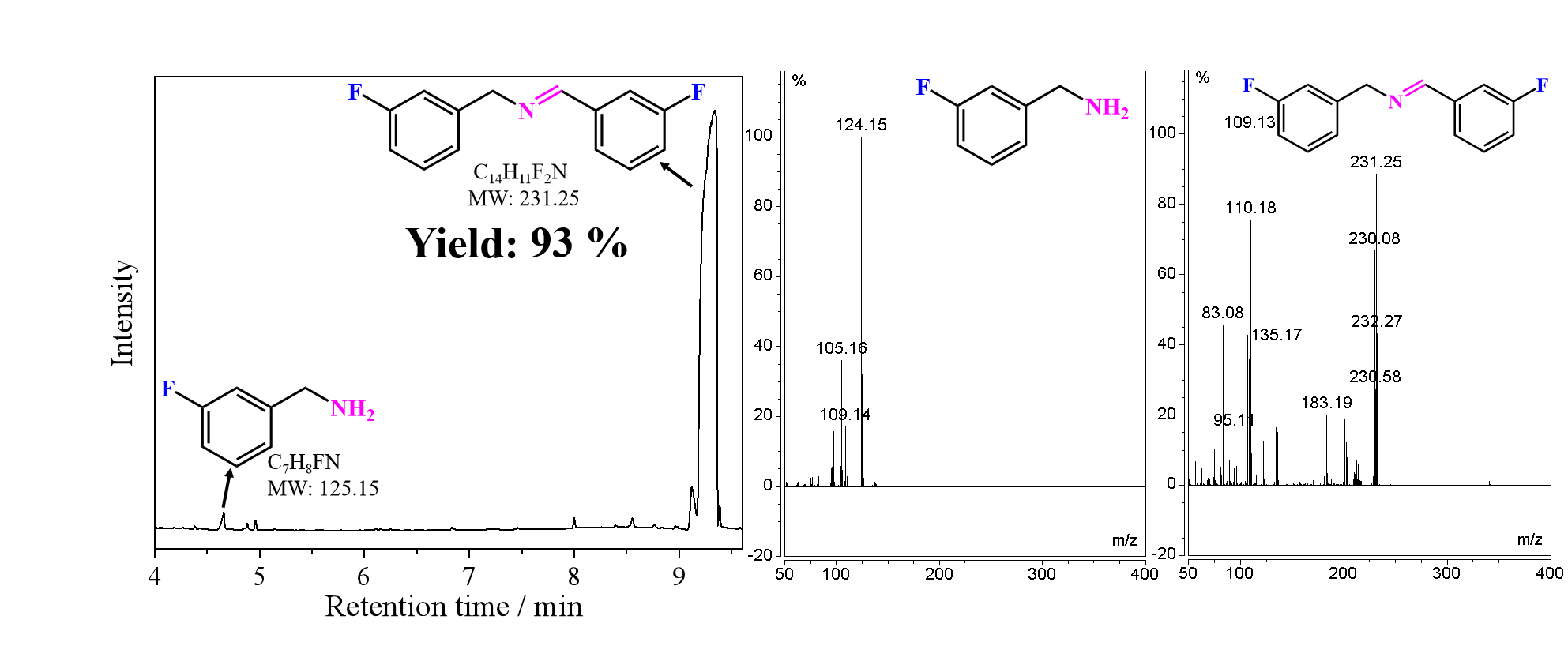


**Figure S55.** GC trace for the imine **24** formation and the corresponding MS spectra.


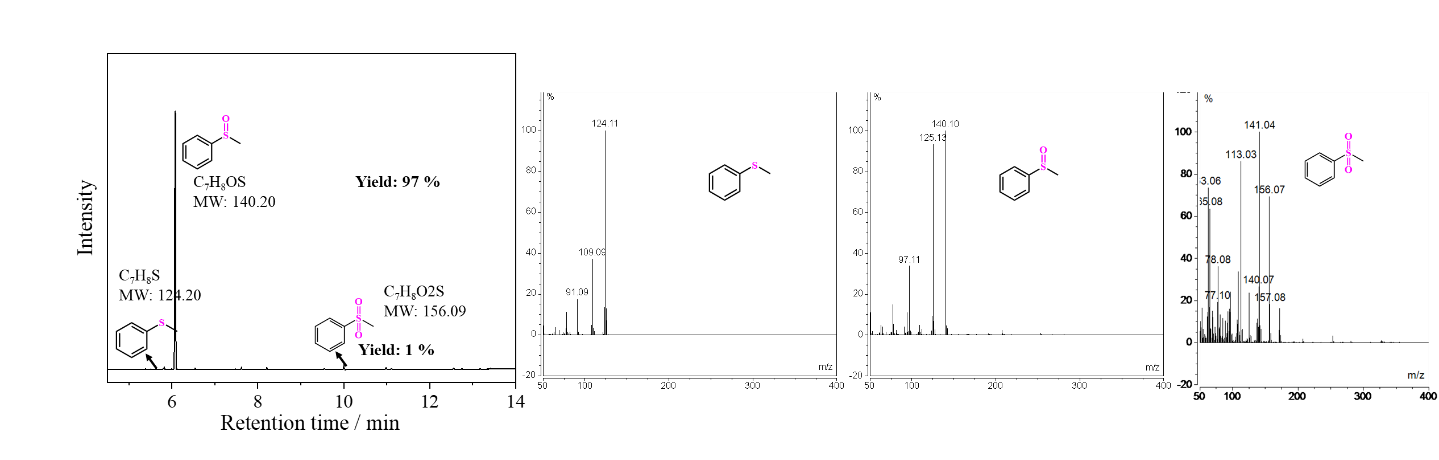


**Figure S56.** GC trace for the sulfoxide **25** formation and the corresponding MS spectra. The yield of identified by-product di-sulfxoide was 1 %.


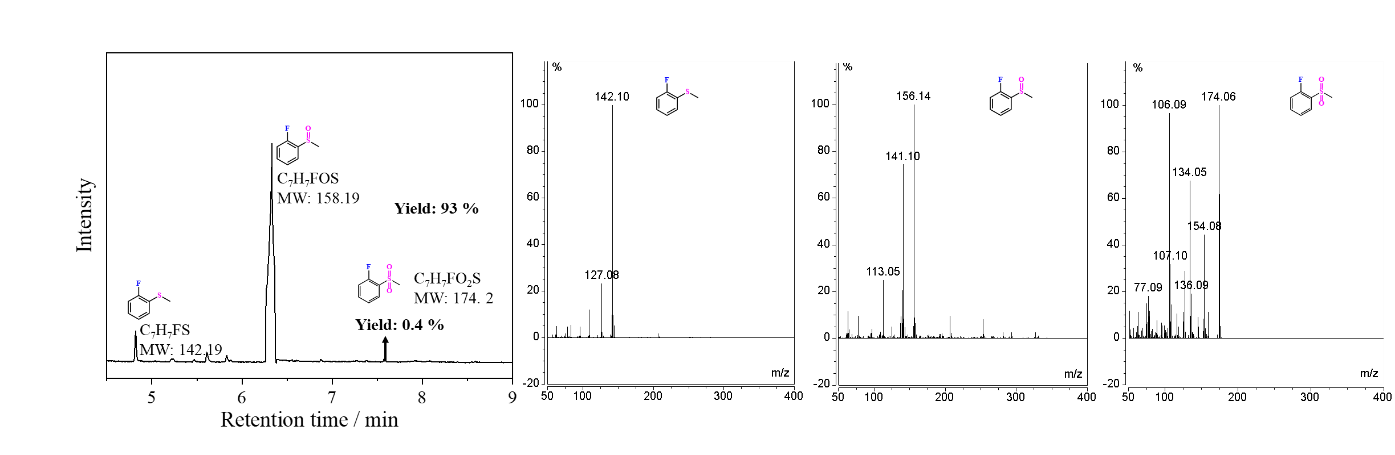


**Figure S57.** GC trace for the sulfoxide **26** formation and the corresponding MS spectra. The yield of identified by-product di-sulfxoide was 0.4 %.


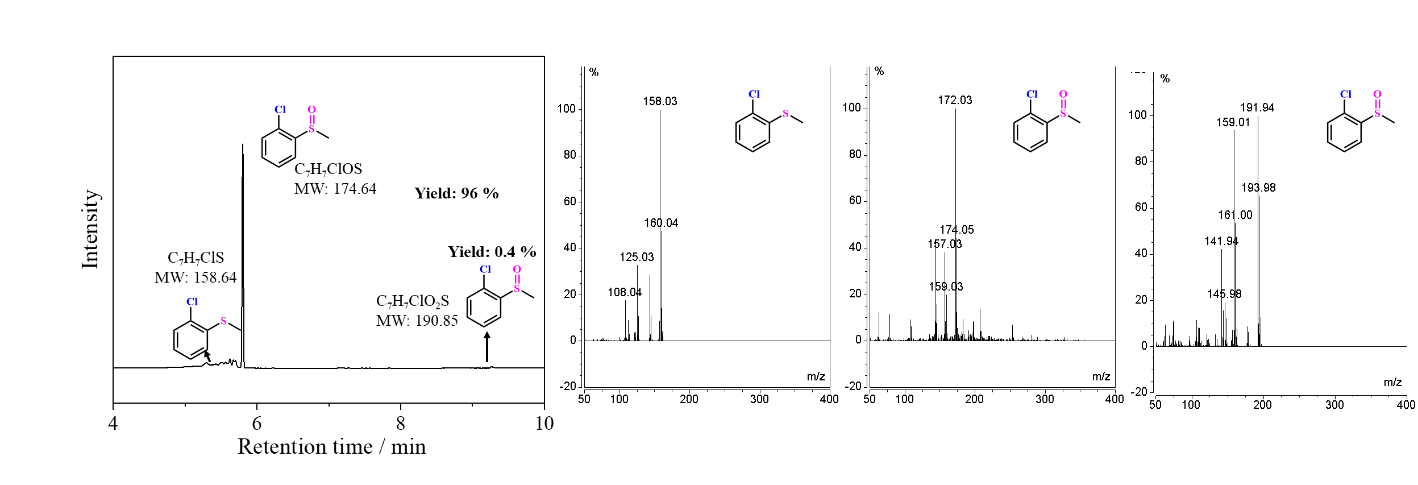


**Figure S58.** GC trace for the sulfoxide **27** formation and the corresponding MS spectra. The yield of identified by-product di-sulfxoide was 0.4 %.


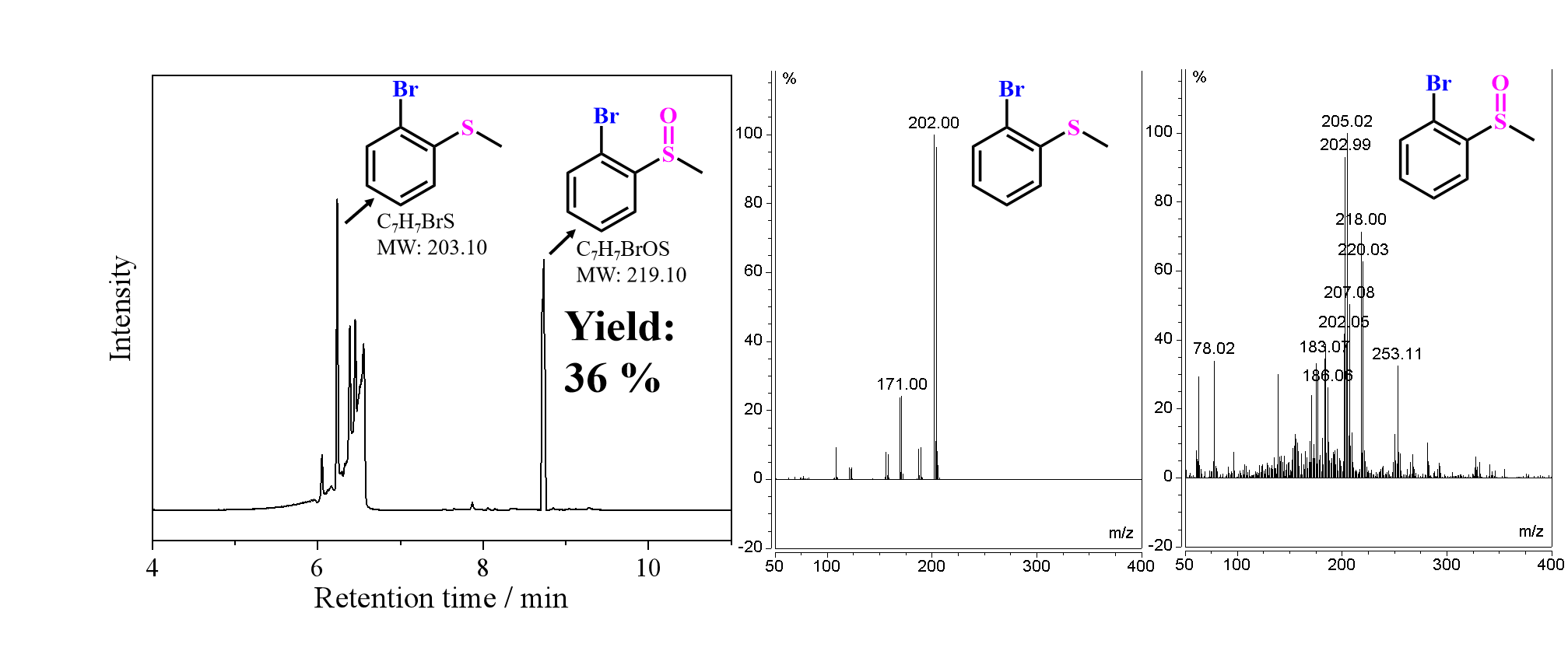


**Figure S59.** GC trace for the sulfoxide **28** formation and the corresponding MS spectra.


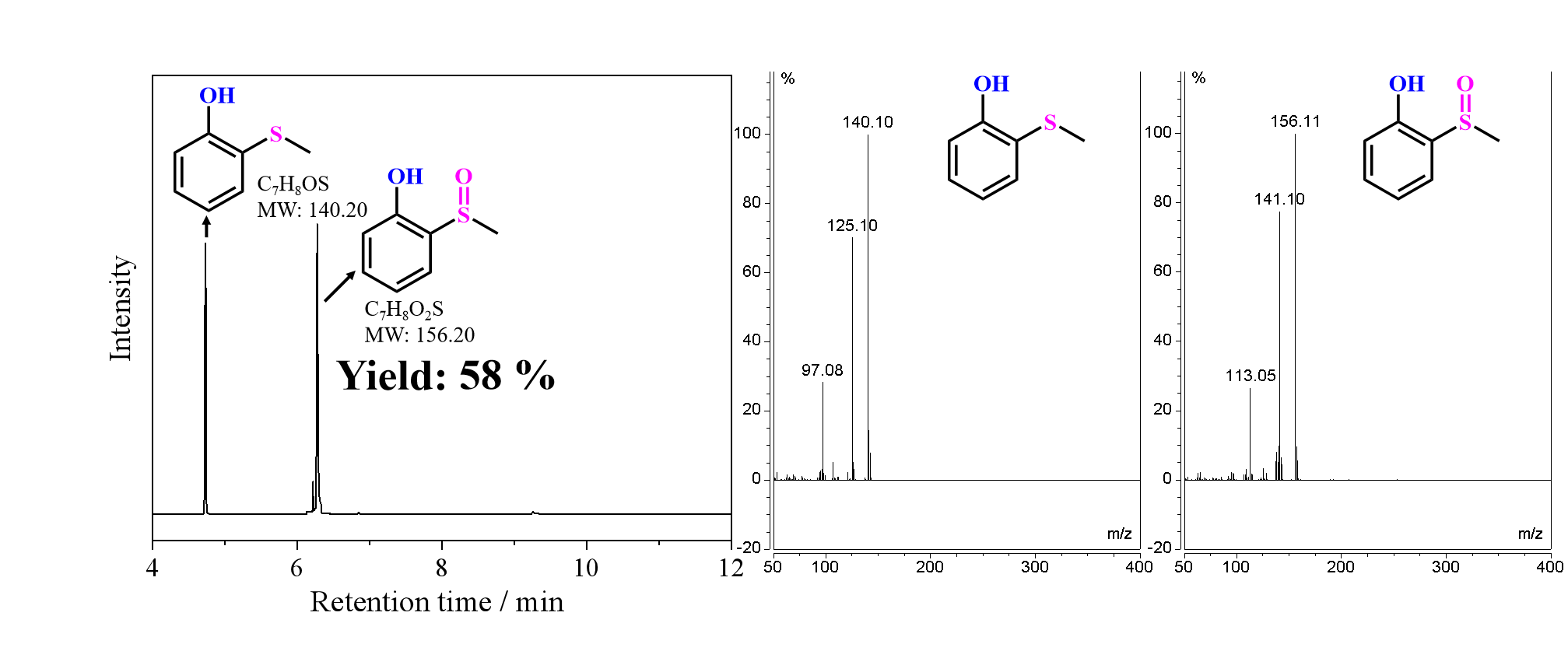


**Figure S60.** GC trace for the sulfoxide **29** formation and the corresponding MS spectra.


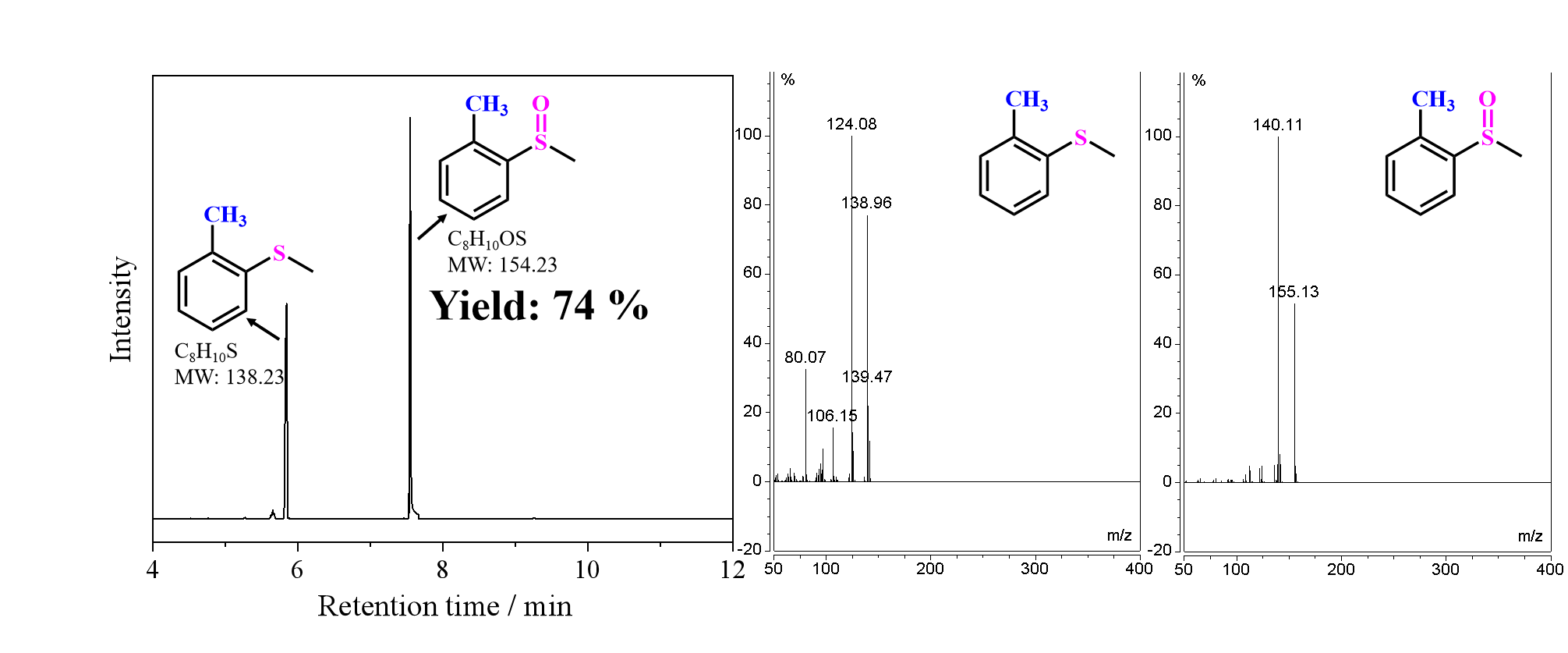


**Figure S61.** GC trace for the sulfoxide **30** formation and the corresponding MS spectra.


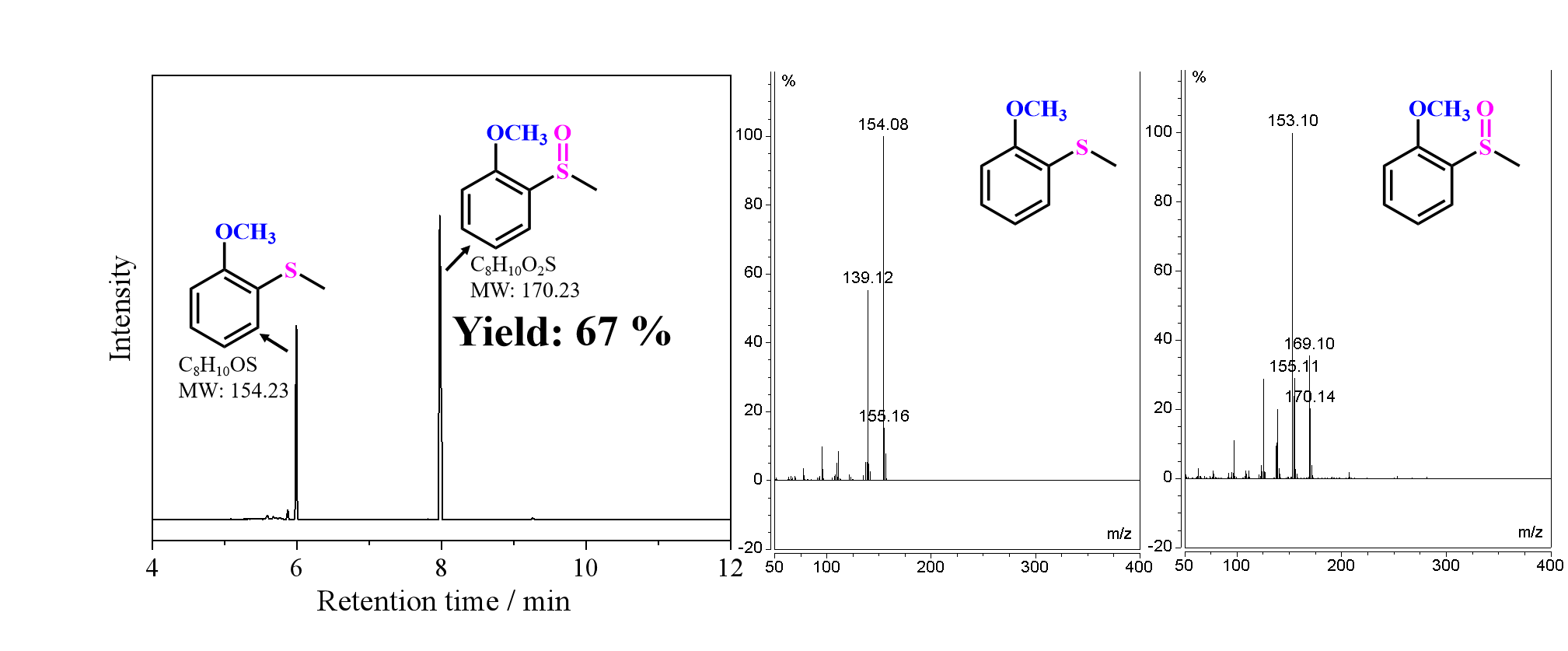


**Figure S62.** GC trace for the sulfoxide **31** formation and the corresponding MS spectra.


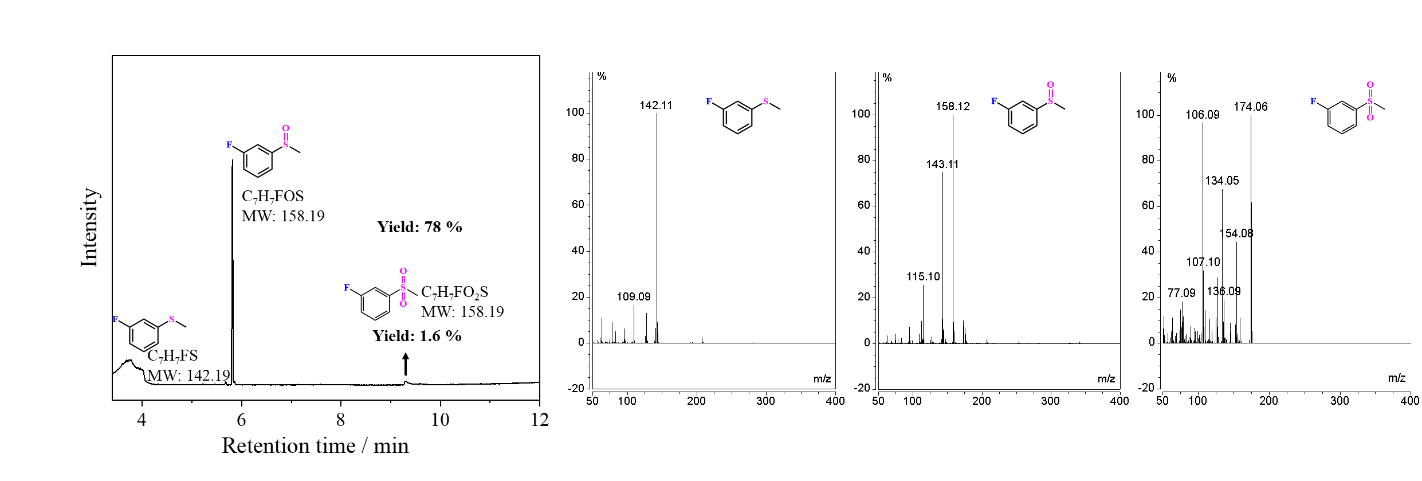


**Figure S63.** GC trace for the sulfoxide **32** formation and the corresponding MS spectra. The yield of identified by-product di-sulfxoide was 1.6 %.


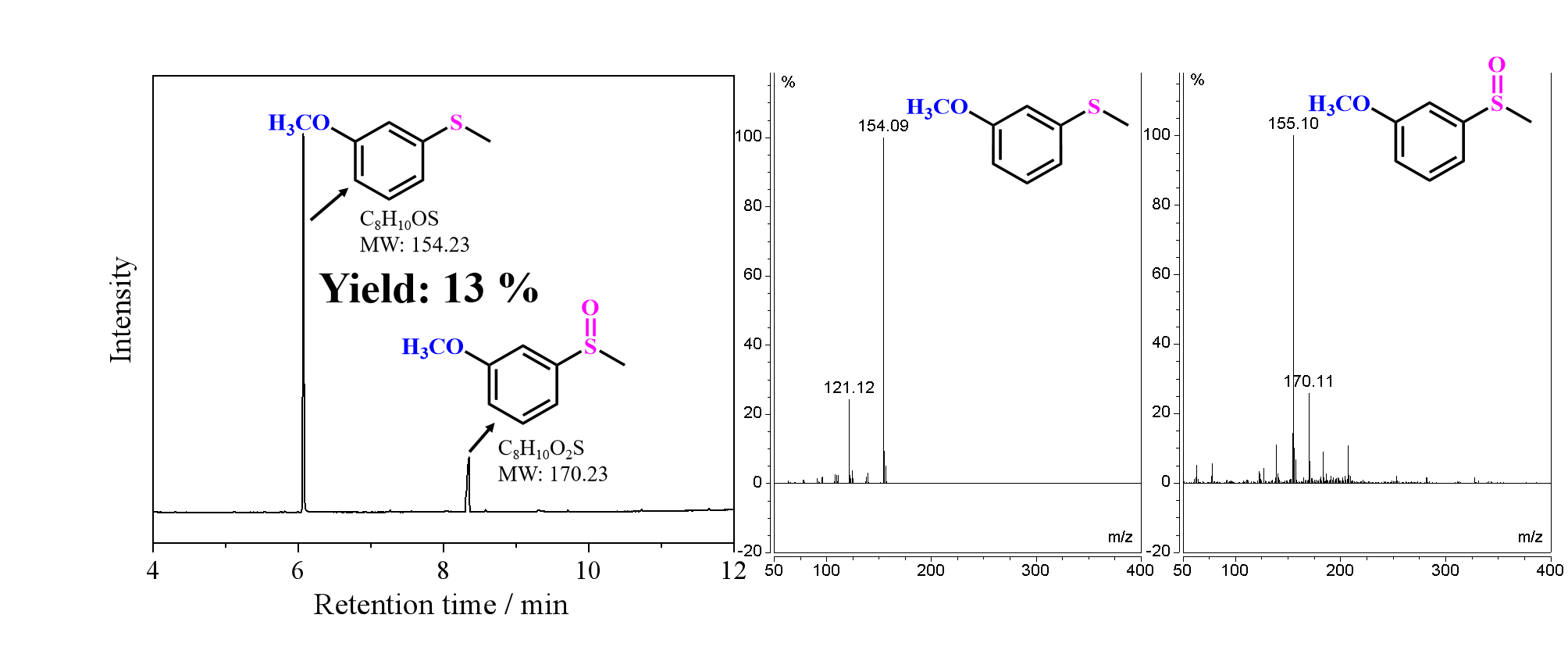


**Figure S64.** GC trace for the sulfoxide **33** formation and the corresponding MS spectra.


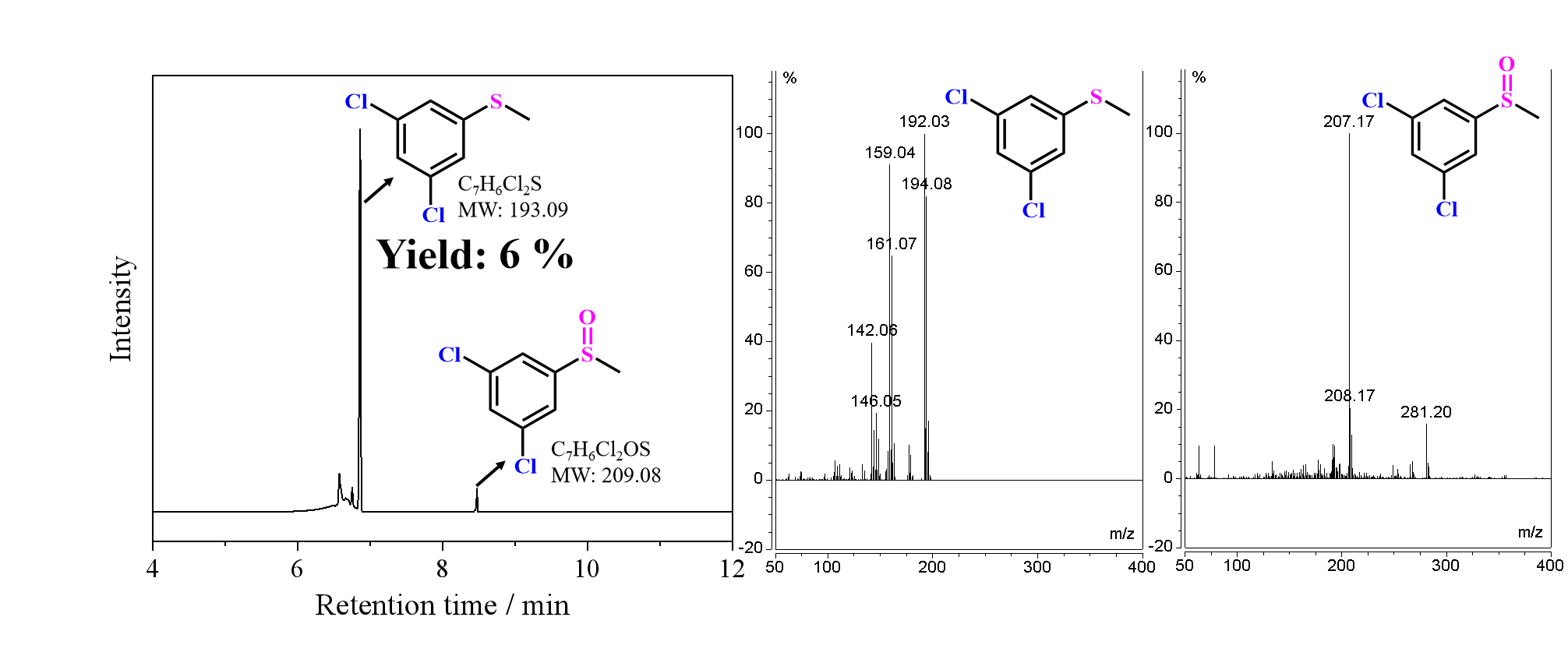


**Figure S65.** GC trace for the sulfoxide **34** formation and the corresponding MS spectra.


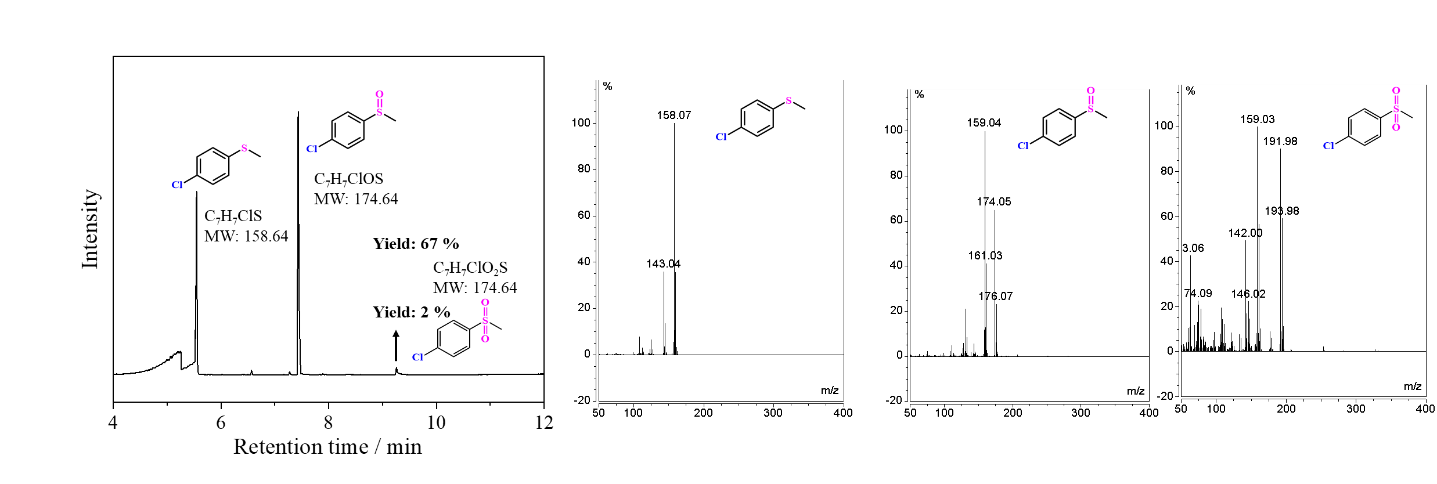


**Figure S66.** GC trace for the sulfoxide **35** formation and the corresponding MS spectra. The yield of identified by-product di-sulfxoide was 2 %.


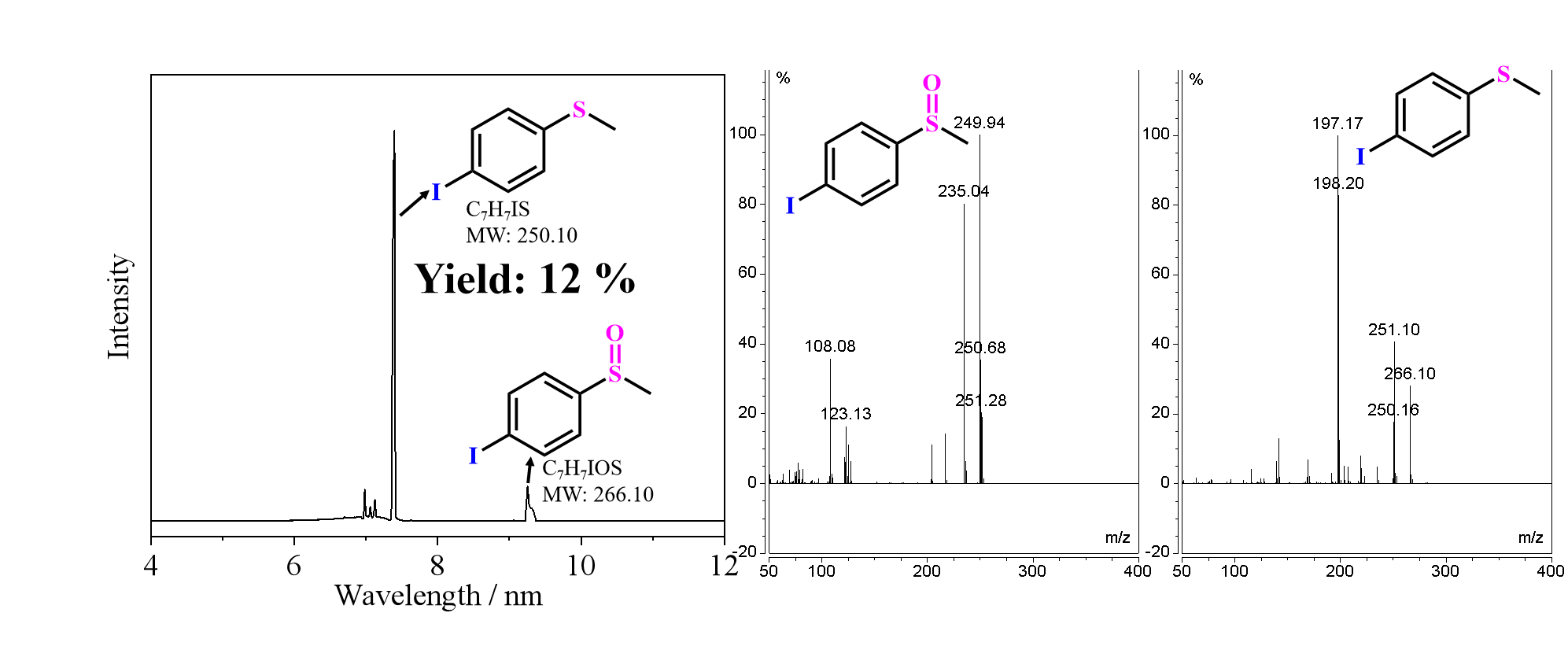


**Figure S67.** GC trace for the sulfoxide **36** formation and the corresponding MS spectra.


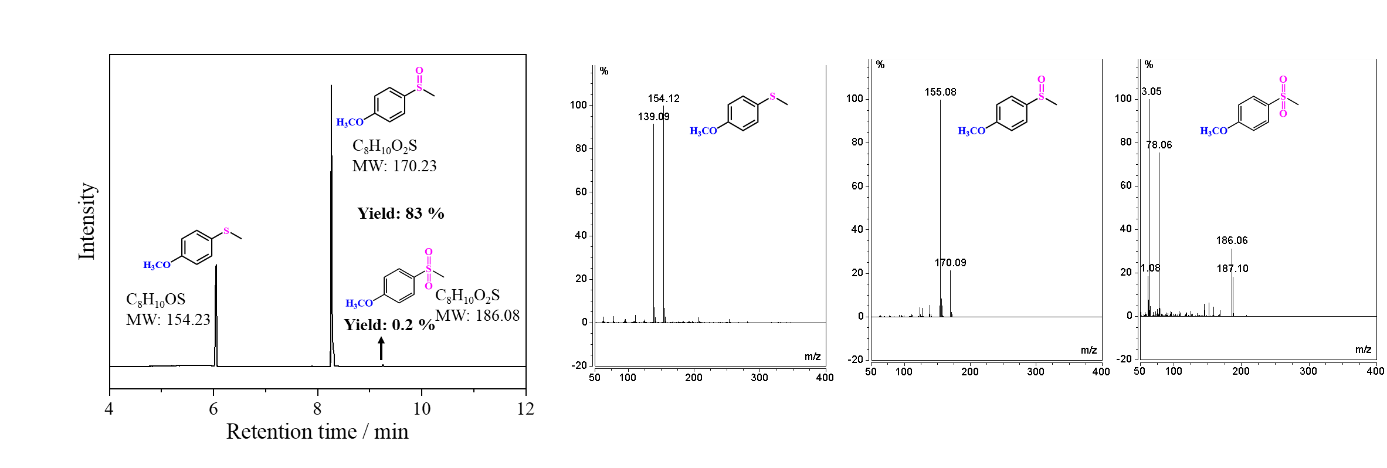


**Figure S68.** GC trace for the sulfoxide **37** formation and the corresponding MS spectra. The yield of identified by-product di-sulfxoide was 0.2 %.


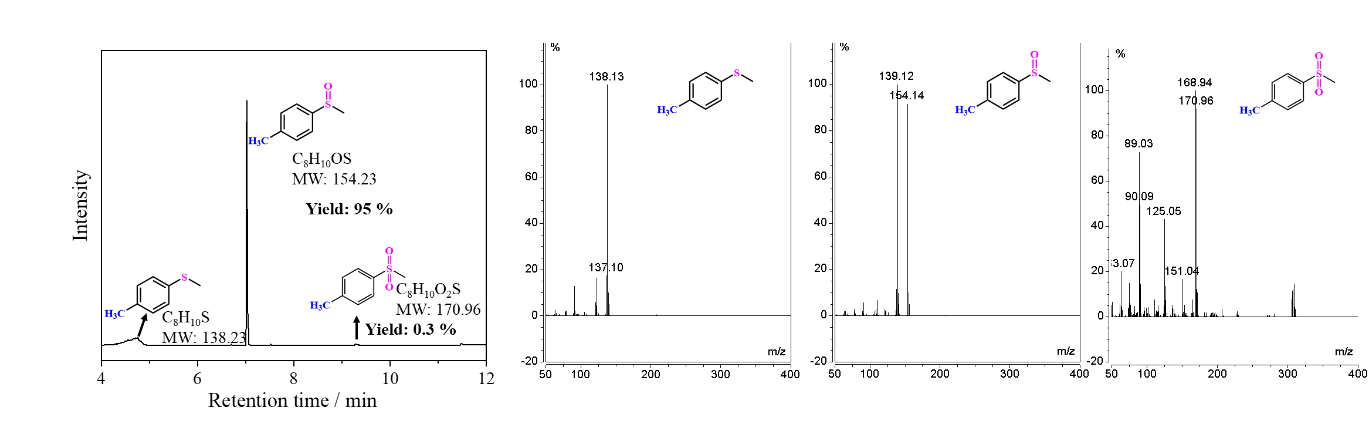


**Figure S69.** GC trace for the sulfoxide **38** formation and the corresponding MS spectra. The yield of identified by-product di-sulfxoide was 0.3 %.


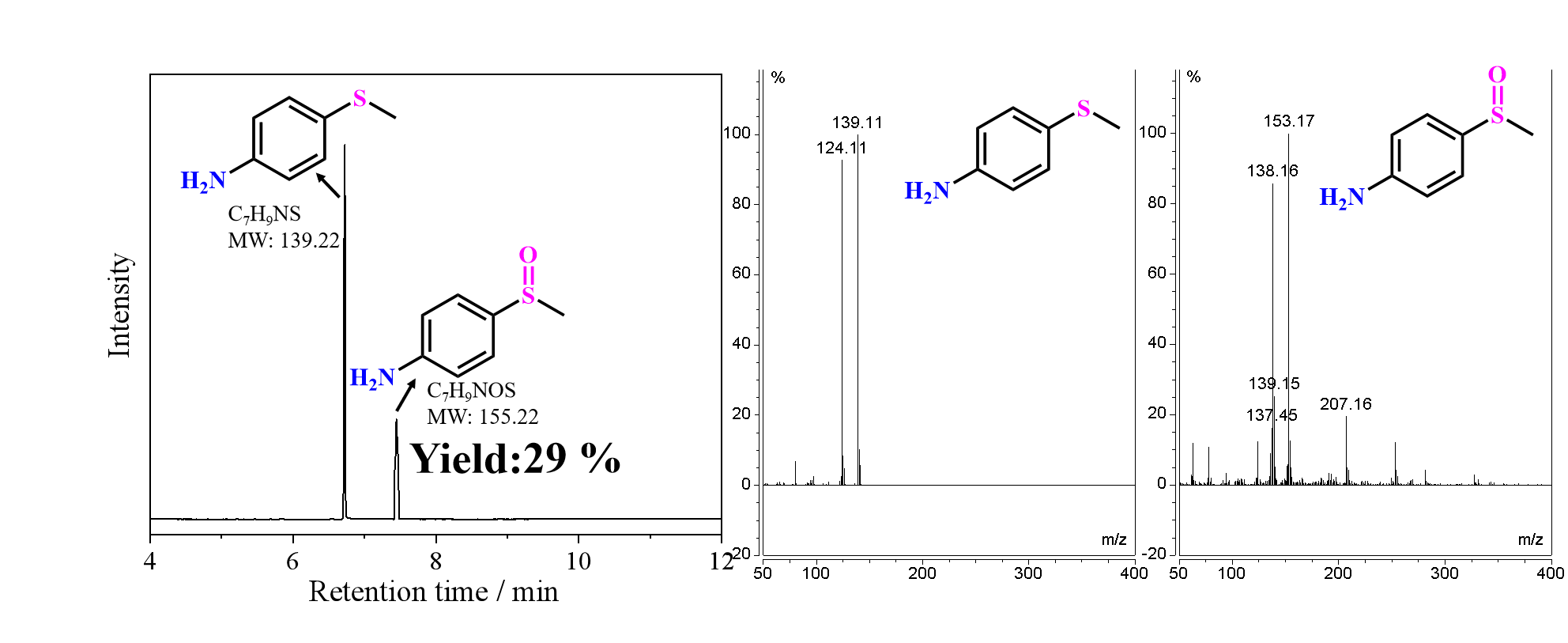


**Figure S70.** GC trace for the sulfoxide **39** formation and the corresponding MS spectra.

**Optimized Cartesian Coordinates**:


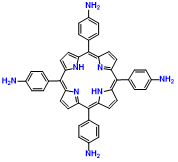


N 2.04146000 0.00448800 0.00001000

N -0.00608000 2.10408600 -0.09519600

N -2.04345800 -0.00450900 -0.00009900

N 0.00336400 -2.10411200 0.09501600

C 2.86717000 -1.08139900 0.01102100

C 4.26560300 -0.66633800 0.01334800

C 4.26259200 0.68523700 -0.01252500

C 2.86230900 1.09405100 -0.01069600

C 2.45559800 2.44403200 -0.02046700

C 1.12363500 2.88575800 -0.03320100

C 0.67323700 4.24192500 0.06721200

C -0.69405700 4.23883200 0.06874800

C -1.13901400 2.88085000 -0.03118300

C -2.46910500 2.43315500 -0.01716800

C -2.86949300 1.08124400 -0.00931800

C -4.26814900 0.66620800 -0.01228000

C -4.26513400 -0.68518000 0.01156400

C -2.86464800 -1.09397900 0.00894600

C -2.45821400 -2.44405200 0.01692300

C -1.12613600 -2.88583900 0.03101000

C -0.67523100 -4.24185200 -0.06888400

C 0.69207200 -4.23894600 -0.06735300

C 1.13650600 -2.88082200 0.03309000

C 2.46649900 -2.43318800 0.02058500

H 5.11815200 -1.32583000 0.03408200

H 5.11221200 1.34850700 -0.03291600

H 1.32547200 5.09498500 0.15269600

H -1.34978400 5.08901800 0.15541100

H -5.12094300 1.32533800 -0.03260000

H -5.11499300 -1.34810000 0.03164300

H -1.32724400 -5.09489000 -0.15554800

H 1.34802200 -5.08914800 -0.15287000

H -0.00428100 1.09254400 -0.15408900

H 0.00084100 -1.09258200 0.15407100

C -3.52906700 3.48981900 0.01200500

C -4.30998400 3.70481800 1.15446800

C -3.77277000 4.30450400 -1.10039400

C -5.29105200 4.68781600 1.18837500

H -4.13921900 3.09516600 2.03467500

C -4.75380800 5.28836200 -1.07907100

H -3.18872000 4.15801800 -2.00225800

C -5.53134400 5.49940600 0.06936200

H -5.87934300 4.83082500 2.08953100

H -4.92825600 5.89477000 -1.96249900

C -3.51343700 -3.50546500 -0.01211800

C -4.29299500 -3.72455000 -1.15472800

C -3.75383500 -4.32069400 1.10060300

C -5.26963300 -4.71195700 -1.18846300

H -4.12466900 -3.11459500 -2.03519700

C -4.73042300 -5.30897200 1.07944600

H -3.17072500 -4.17115700 2.00257400

C -5.50664000 -5.52407100 -0.06912800

H -5.85698800 -4.85803900 -2.08973500

H -4.90240400 -5.91574900 1.96310600

C 3.51035700 3.50618600 0.00440000

C 4.28833200 3.73245600 1.14658000

C 3.75241400 4.31418400 -1.11322300

C 5.26570900 4.71947100 1.17497400

H 4.11978400 3.12706200 2.03016300

C 4.72971400 5.30187100 -1.09744000

H 3.17139000 4.15782300 -2.01538300

C 5.50633400 5.52229400 0.04978200

H 5.84500100 4.87815400 2.07933300

H 4.89598400 5.90975400 -1.98146700

N -6.52742500 -6.47459200 -0.07687500

H -6.79833400 -6.80934300 -0.98970000

H -6.42140800 -7.22752700 0.58684500

N -6.55640200 6.44532400 0.07723200

H -6.82916200 6.77831700 0.99015000

H -6.45345400 7.19914100 -0.58597100

N 6.44632800 6.55239500 0.09214600

H 7.18059900 6.43758100 0.77513600

H 6.80655900 6.84369200 -0.80462800

C 3.52601200 -3.49058800 -0.00429300

C 4.30535300 -3.71288800 -1.14633800

C 3.77144100 -4.29787100 1.11310300

C 5.28720900 -4.69543700 -1.17481300

H 4.13430500 -3.10791600 -2.02972900

C 4.75322600 -5.28110300 1.09723400

H 3.18947500 -4.14446900 2.01516500

C 5.53115500 -5.49757000 -0.04984700

H 5.86749500 -4.85114300 -2.07905300

H 4.92199400 -5.88855200 1.98108400

N 6.47587500 -6.52337100 -0.09230500

H 7.20976500 -6.40488600 -0.77509200

H 6.83738300 -6.81305900 0.80448700


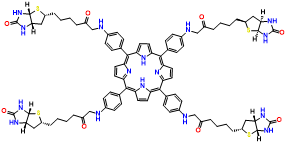


N -2.09536700 2.12367400 -0.63387400

N 0.83400900 1.99852400 -0.79792300

N 0.63720000 -0.89855700 -0.40984900

N -2.29485100 -0.78122800 -0.30744100

C -3.45438700 2.00814300 -0.61067400

C -4.08708800 3.29675000 -0.87088500

C -3.08413000 4.18832600 -1.03202200

C -1.84068600 3.44228700 -0.87053000

C -0.56721000 4.04018000 -0.96083000

C 0.65647000 3.35755700 -0.89729700

C 1.96684000 3.93700600 -0.91770200

C 2.88264200 2.92495000 -0.83821700

C 2.17159300 1.68306000 -0.76774400

C 2.72688100 0.39752700 -0.68217800

C 1.98979200 -0.79784900 -0.55264600

C 2.60840600 -2.11920200 -0.56602600

C 1.60522900 -3.01135500 -0.41122400

C 0.37573200 -2.23322400 -0.30360900

C -0.89246900 -2.82158700 -0.12179700

C -2.11123000 -2.12679600 -0.09638400

C -3.40870900 -2.67748100 0.16041600

C -4.32421200 -1.66435900 0.09006500

C -3.62558900 -0.45098100 -0.21384000

C -4.18553200 0.82511900 -0.37898900

H -5.14784500 3.47596600 -0.94109100

H -3.16568800 5.23934700 -1.25821400

H 2.16761600 4.99393300 -0.97258900

H 3.95568700 3.01660700 -0.81811500

H 3.65870700 -2.32270900 -0.69882200

H 1.67505800 -4.08693100 -0.38986200

H -3.59850100 -3.71376600 0.38487800

H -5.38786300 -1.73440800 0.24505900

H 0.08306100 1.32401700 -0.71306600

H -1.54774100 -0.11614500 -0.46700000

C 4.21985600 0.30734900 -0.72864100

C 4.95180700 -0.09101500 0.39693900

C 4.93279500 0.61806500 -1.89173100

C 6.33667800 -0.17052400 0.35693900

H 4.42954000 -0.33288100 1.31522400

C 6.32114200 0.53961900 -1.95063700

H 4.38924800 0.91751900 -2.78070100

C 7.03880100 0.14337800 -0.81506300

H 6.88085900 -0.47480600 1.24625800

H 6.85212500 0.77579100 -2.85932000

C -0.96398100 -4.30513900 0.06369800

C -0.46980100 -4.91510100 1.22180900

C -1.52782500 -5.13001800 -0.91729200

C -0.52744400 -6.29232600 1.41312100

H -0.03408000 -4.29739100 1.99879200

C -1.58948500 -6.50589800 -0.74474400

H -1.91112300 -4.68955100 -1.83047400

C -1.09239200 -7.10416400 0.42132400

H -0.14513200 -6.74362000 2.31511700

H -2.02368400 -7.12409200 -1.52508000

C -0.49512700 5.52572000 -1.13480900

C -0.06429300 6.09376200 -2.34012900

C -0.86448600 6.39149500 -0.09967000

C 0.01183500 7.47215800 -2.49715300

H 0.21502000 5.44664100 -3.16356700

C -0.81106700 7.77322700 -0.25534100

H -1.21331400 5.97647400 0.83882000

C -0.35378400 8.33158400 -1.45353100

H 0.36157300 7.89226800 -3.43418300

H -1.14539300 8.41374900 0.54967000

N -1.19016100 -8.50716800 0.52536200

H -1.62222300 -8.96233200 -0.26434500

N 8.44433400 0.03516500 -0.78059800

H 8.82468100 -0.27597000 0.10038600

N -0.30241100 9.72682400 -1.67743000

H -0.52170400 10.03782500 -2.61533800

C -5.67660200 0.91902800 -0.29327800

C -6.49954900 0.35341500 -1.27329700

C -6.29596400 1.57690400 0.77669600

C -7.88763100 0.43159900 -1.20790200

H -6.04479800 -0.15165700 -2.11797100

C -7.67872100 1.66040400 0.85991900

H -5.68646700 2.01992900 1.55565700

C -8.49170400 1.08982900 -0.12929800

H -8.50485000 -0.00376400 -1.97793100

H -8.13409900 2.16949700 1.70437000

N -9.88784600 1.21876700 0.02150600

H -10.18101400 1.72821600 0.84126400

C 4.99097100 15.08306100 6.29265800

C 4.45129300 13.31473600 4.90456300

C 4.99463200 12.77282200 6.22419200

C 3.45473300 12.28321100 4.40115400

C 5.67587000 11.46519100 5.86744500

H 2.56796600 12.27288900 5.03962500

H 6.61897900 11.62205000 5.34362600

N 4.06640000 14.65551900 5.32218900

N 5.71045500 13.94637600 6.70337100

O 5.13876500 16.21087700 6.69599100

H 4.13985300 12.54557800 6.87882600

H 5.28344300 13.35724000 4.18382900

H 5.82576900 10.79867700 6.71588600

S 4.41635300 10.68195900 4.72424000

C 3.04967100 12.42143300 2.93663900

C 2.07719800 11.34273700 2.44601900

H 3.94979300 12.42112900 2.31198200

H 2.57544400 13.40492500 2.81353500

C 1.64159000 11.55790500 0.99342600

H 2.54774100 10.35777000 2.54961200

H 1.19257800 11.33260000 3.09574800

C 0.67912500 10.47354100 0.50026700

H 1.16022700 12.53250900 0.88044300

H 2.52307200 11.57889900 0.34319100

H 1.15380900 9.48873000 0.52120300

H -0.18009800 10.41268200 1.17975100

C 0.14732600 10.77660600 -0.89383600

O 0.11984800 11.90703500 -1.34576200

C -10.89930900 0.71657300 -0.76833800

C -12.30178300 0.99778300 -0.22886600

O -10.71307600 0.08373100 -1.79069800

C -13.34199600 1.12283700 -1.34508500

H -12.30325200 1.89495700 0.40015400

H -12.56473900 0.15758800 0.42710600

C -14.77529000 1.22456200 -0.81502600

H -13.24100800 0.25891800 -2.00728600

H -13.11121600 2.00388500 -1.95459800

C -15.81254400 1.35626800 -1.93600600

H -14.86029500 2.08593500 -0.14180500

H -15.00546500 0.33674900 -0.21235400

C -17.25828400 1.40220800 -1.45022000

H -15.60388700 2.25212800 -2.53123900

H -15.71394900 0.49414300 -2.60993100

C -18.30789500 1.57722300 -2.53572900

H -17.48325600 0.51910000 -0.84723300

S -17.59980600 2.93158400 -0.38373600

C -19.59542500 1.98012100 -1.82121800

N -18.80426400 0.47510300 -3.34735400

H -17.99377200 2.41833000 -3.17420500

C -19.27582600 3.29525800 -1.13601000

N -20.54263600 1.85680900 -2.91954100

H -19.80398000 1.23093200 -1.04272100

C -20.12349500 0.78447200 -3.72733000

H -19.20352900 4.12140700 -1.84354200

H -19.96147500 3.55226700 -0.32959700

O -20.77141900 0.23419600 -4.58376300

C -0.80857500 -9.33261400 1.56068800

C -1.15413000 -10.80493600 1.33677600

O -0.26863400 -8.94162500 2.57854000

C -0.16495400 -11.74736300 2.02746400

H -1.21764200 -11.03105100 0.26654400

H -2.15999800 -10.96132300 1.74800100

C -0.60021500 -13.21465500 1.97071700

H -0.05049300 -11.42401000 3.06544000

H 0.82124500 -11.63730800 1.56230600

C 0.39711900 -14.15398500 2.65839600

H -0.72557400 -13.52345300 0.92605300

H -1.58380900 -13.32202400 2.44546200

C -0.03161000 -15.61821000 2.67855900

H 1.37735000 -14.07214800 2.17582100

H 0.52515800 -13.82942200 3.70023900

C 0.95729300 -16.58051800 3.31626100

H -1.01258300 -15.71875800 3.14935300

S -0.15181400 -16.34653700 0.93278900

C 0.52350300 -17.98061900 2.88955800

N 1.06172100 -16.77657600 4.75515800

H 1.94876000 -16.38170600 2.87889900

C 0.63321700 -17.98792200 1.37653900

N 1.37080000 -18.78327800 3.75943200

H -0.53661700 -18.10680500 3.15606200

C 1.51208000 -18.09140900 4.97577900

H 1.66933700 -18.01713600 1.03894500

H 0.06047800 -18.77954100 0.89511100

O 1.93872900 -18.53957400 6.01175900

C 9.36508600 0.33288200 -1.76193300

C 10.81776500 0.16743000 -1.31478600

O 9.06909100 0.71718100 -2.87766500

C 11.75150800 -0.17584600 -2.47794400

H 10.89491300 -0.58707200 -0.52387600

H 11.12394800 1.12109400 -0.86499500

C 13.22971400 -0.17079900 -2.07724500

H 11.57326000 0.53973000 -3.28493300

H 11.48137200 -1.15978000 -2.87763600

C 14.15986300 -0.51478200 -3.24616400

H 13.39453500 -0.88556900 -1.26216400

H 13.49752000 0.81717500 -1.68149800

C 15.64547200 -0.46405400 -2.90174200

H 13.91409900 -1.50880200 -3.63591500

H 13.98127000 0.20120600 -4.06017900

C 16.59163700 -0.83999100 -4.03056400

H 15.90789600 0.51823300 -2.50134100

S 16.11738300 -1.76161200 -1.60315500

C 17.94877500 -1.09648000 -3.38021600

N 16.98654800 0.08851300 -5.08016300

H 16.23614700 -1.78800600 -4.46495300

C 17.72233600 -2.25780400 -2.43085000

N 18.78552900 -1.18255100 -4.56815900

H 18.21431700 -0.21221500 -2.78156200

C 18.27010200 -0.28462000 -5.52016700

H 17.60091900 -3.20372200 -2.95892800

H 18.48600400 -2.35492600 -1.66023200

O 18.82298200 0.09381700 -6.52375700

H 6.06510900 13.98674300 7.64859500

H 3.81368000 15.35746300 4.64081300

H -21.53821900 1.89980700 -2.75272300

H -18.23613500 0.07606200 -4.08144700

H 1.49443000 -16.07781300 5.34284100

H 1.22416000 -19.77875700 3.85101000

H 19.79307300 -1.19039200 -4.49291500

H 16.34364500 0.33955100 -5.81809500

**End**
